# Visible-Light-Activated Carbon Monoxide Release from Porphyrin–Flavonol Hybrids

**DOI:** 10.1021/jacs.3c11426

**Published:** 2023-12-29

**Authors:** Andrea Ramundo, Jiří Janoš, Lucie Muchová, Mária Šranková, Jakub Dostál, Miroslav Kloz, Libor Vítek, Petr Slavíček, Petr Klán

**Affiliations:** † Department of Chemistry, Faculty of Science, Masaryk University, Kamenice 5, 62500 Brno, Czech Republic; ‡ RECETOX, Faculty of Science, Masaryk University, Kamenice 5, 62500 Brno, Czech Republic; § Department of Physical Chemistry, University of Chemistry and Technology, Technická 5, 16628 Prague 6, Czech Republic; ∥ Institute of Medical Biochemistry and Laboratory Diagnostics, and 4th Department of Internal Medicine, General University Hospital in Prague and First Faculty of Medicine, Charles University, Na Bojišti 3, 12108 Prague 2, Czech Republic; ⊥ ELI Beamlines Facility, The Extreme Light Infrastructure ERIC, Za Radnicí 835, 25241 Dolní Břežany, Czech Republic

## Abstract

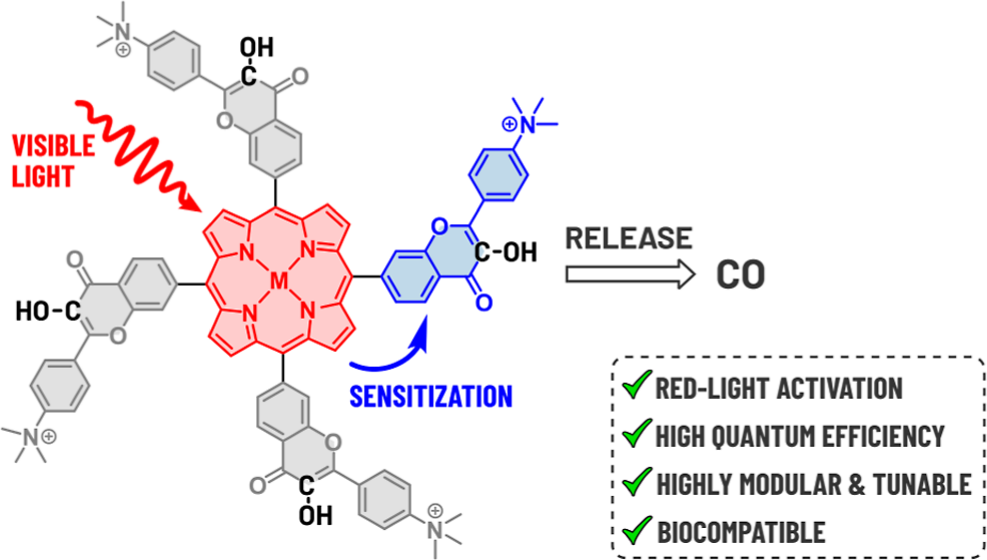

We report on porphyrin–flavonol hybrids consisting of a porphyrin antenna and four covalently bound 3-hydroxyflavone (flavonol) groups, which act as highly efficient photoactivatable carbon monoxide (CO)-releasing molecules (photoCORMs). These bichromophoric systems enable activation of the UV-absorbing flavonol chromophore by visible light up to 650 nm and offer precise spatial and temporal control of CO administration. The physicochemical properties of the porphyrin antenna system can also be tuned by inserting a metal cation. Our computational study revealed that the process occurs via endergonic triplet–triplet energy transfer from porphyrin to flavonol and may become feasible thanks to flavonol energy stabilization upon intramolecular proton transfer. This mechanism was also indirectly supported by steady-state and transient absorption spectroscopy techniques. Additionally, the porphyrin–flavonol hybrids were found to be biologically benign. With four flavonol CO donors attached to a single porphyrin chromophore, high CO release yields, excellent uncaging cross sections, low toxicity, and CO therapeutic properties, these photoCORMs offer exceptional potential for their further development and future biological and medical applications.

## Introduction

Carbon monoxide (CO), a small gaseous signaling molecule with inherent toxicity,^1^ controls various critical cellular processes, with mitochondria being its primary target, and manifests anti-inflammatory, antiproliferative, and cytoprotective properties.^2,3^ It soon gained attention as a promising therapeutic agent in several settings, such as organ transplantation or cancer treatments.^4−6^ Remarkably, CO can stimulate cancer cells to switch to a pure oxidative metabolism in an anti-Warburg fashion,^7^ leading to growth inhibition and cell death.^8,9^ Many studies suggest that CO is a useful gaseous drug, but its acute toxicity poses severe limitations in its administration under a clinical scenario. A controlled, targeted release is therefore essential.

Photoactivatable CO-releasing molecules (photoCORMs) can serve as prodrugs that release CO after the absorption of a photon.^4^ Light as a control stimulus provides the required spatiotemporal control to circumvent CO toxicity.^10^ Activation wavelengths in the so-called “phototherapeutic window” (650–900 nm) allow for a deep light penetration without impairing cellular damage.^11^ Therefore, the development of red- or near-infrared (NIR)-absorbing photoCORMs is desirable. The first examples of photoCORMs included transition metal–carbonyl complexes,^12^ cyclopropenones,^13,14^ or 1,2-dioxolane-3,4-diones,^15^ all absorbing in the UV region. α-Diketones^16^ (λ_max_ = 470 nm) and a xanthene-based cromophore^17^ (λ_max_ = 488 nm) have been developed to deliver CO under visible-light activation. A *meso*-carboxy BODIPY^18^ (λ_max_ = 652 nm) was the first red-light activatable organic photoCORM, yet suffering from a poor uncaging cross-section (Φ_CO_ε_max_).

**Scheme 1:**
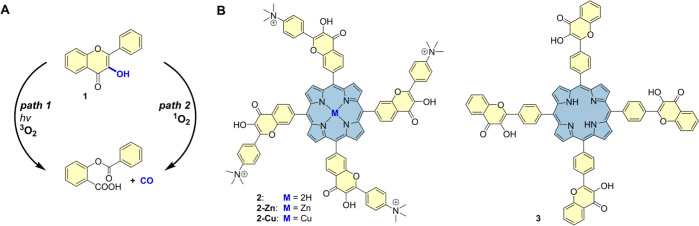
Flavonol Chemistry and Studied Compounds; (A) (Photo)decarbonylation of Flavonol 1; (B) Structures of Porphyrin–Flavonol Hybrids 2 and 3 Composed of a Central Light-Harvesting Porphyrin Sensitizer (Blue) and Four CO-Releasing Flavonol Moieties (Yellow) Studied in This Work

3-Hydroxyflavone **1** (flavonol; 3-hydroxy-2-phenylchromen-4-one, Scheme 1A) belongs to the family of flavonoids, a class of naturally occurring molecules.^19^ It undergoes photooxygenation to give CO and a salicylic acid derivative as the main photoproducts upon irradiation with UV light (λ_max_ = 355 nm).^20,21^ Several attempts have been made to improve flavonol absorption properties. π-Extended flavonols with λ_max_ in the 400–540 nm range have been developed,^22−24^ although still retaining the same mechanism of photodecarbonylation.^25^ Our laboratory used an alternative approach of combining an established cyanine 7 scaffold with the flavonol moiety, pushing the absorption of the resulting cyanine-flavonol hybrids to the NIR region (λ_max_ = 750–790 nm).^26,27^

More recently, 3,4′-dihydroxyflavone derivatives have been embedded in a polymer matrix with 5-(4-hydroxyphenyl)-10,15,20-triphenylporphyrin used as a ^1^O_2_ generator to trigger CO release.^28^ Still, the combined requirements for a successful biological application of a photoCORM, such as water solubility, absorption near or within the phototherapeutic window, high quantum efficiency of CO release, and very low or no toxicity, remain a challenge.

In a recent study, some of us showed that porphyrins could be repurposed as a new class of photoactivatable molecules releasing biologically relevant substances, such as indibulin or methotrexate, attached to the porphyrin *meso*-methyl position upon visible-light activation.^29^

In this work, we used a porphyrin moiety operating as an antenna and a triplet energy donor that transfers triplet energy to four CO-releasing flavonol moieties attached to the *meso* positions (**2** and **3**, Scheme 1B). The introduction of metal ions into the porphyrin core of **2** was aimed to affect the photochemical efficiencies, singlet oxygen production, and other physicochemical properties of the hybrids. Steady-state optical and transient absorption (TA) spectroscopies and quantum-chemical calculations were used to evaluate the reaction mechanism that involves the sensitization of flavonol by triplet-excited porphyrin. We also studied the systems’ applicability in cell culture experiments through fluorescence microscopy, cytotoxicity determination, and high-resolution respirometry to assess mitochondrial metabolism.

## Results and Discussion

In our search for photoCORMs operating at the edge of or within the phototherapeutic window,^30^ we designed structures **2** and **3**, consisting of the central porphyrin moiety as an antenna bearing four UV-absorbing flavonol units (λ_max_ = 355 nm) in the four meso positions, to improve atom economy of the CO production (up to 4 equiv of CO released per one starting compound). The nature of this aromatic macrocycle, absorbing across the entire visible spectrum (400–670 nm),^31,32^ would allow for fine-tuning of its spectroscopic and photochemical properties by incorporating metal ions into the porphyrin core and other structural modifications.^33^

To experimentally determine whether porphyrins can sensitize CO release from flavonols, methanol solutions containing either *meso*-(tetra-4-carboxyphenyl)porphyrin (TcPP) or its zinc complex (ZnTcPP) and a flavonol derivative (**1**, or its more water-soluble 4′-trimethylammonium analog—see the Supporting Information) were irradiated with red light (600 or 650 nm) where flavonol does not absorb. CO was liberated with excellent yields of up to 0.96 equiv, whereas only 0.08–0.14 equiv of CO were generated upon direct irradiation of flavonols at 365 nm in the absence of a porphyrin sensitizer (Table S1).

Two possible mechanisms of CO release from flavonol derivatives have been postulated.^20,25^ The first one involves the reaction between triplet-excited flavonol and ground-state oxygen (^3^O_2_, path 1, Scheme 1A) or ground-state flavonol and singlet oxygen (^1^O_2_) formed in situ by photosensitization (path 2). These two orthogonal mechanisms can operate simultaneously, but their relative efficiencies are driven by the acid/base equilibria related to the OH group (p*K*_a_ = 8.7^34^). For instance, path 2 becomes predominant only when the conjugate base of **1** is considered.^24^ However, only the acid form of **1** was detected by UV–vis spectroscopy in methanol. Indeed, we found the reactivity of ground-state flavonol **1** toward ^1^O_2_ to be very low (*k*_Σ_ = 2.5 × 10^5^ M^–1^ s^–1^). The calculated photodecarbonylation quantum yield due to the reaction with singlet oxygen was found to be 10^–4^, which is lower by more than an order of magnitude than that of the CO production via direct irradiation (Supporting Information). In addition, ZnTcPP was irradiated as a singlet oxygen generator in the presence of **1** at 600 nm, where ZnTcPP absorbs exclusively, in a methanol solution with and without a large excess of NaN_3_ as a singlet oxygen scavenger (Table S2). The CO production was the same, providing experimental evidence that ^1^O_2_ is not involved in the release of CO from flavonol **1** when porphyrin is used as a sensitizer.

**Scheme 2:**
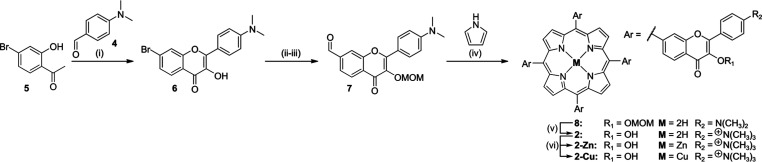
Synthesis of the Porphyrin–Flavonol Hybrids 2, 2-Zn, and 2-Cu Reaction conditions: (i) NaOH, H_2_O_2_, CH_3_OH, 55%; (ii) MOMBr, CH_2_Cl_2_, 0 °C, 89%; (iii) Pd(OAc)_2_, cataCXium, TMEDA, CO/H_2_ 8 atm, toluene, 100 °C, 54%; (iv) pyrrole, TFA, DDQ, CHCl_3_, 23 °C, 18%; (v) CH_3_OTf, HOTf, CH3CN, 23 °C, 54%; (vi) **2-Zn**: Zn(OTf)_2_, DMF, 130 °C, 77%. **2-Cu**: Cu(OTf)_2_, CH_3_OH, 23 °C, 73%.

These encouraging results led to the design, synthesis, and studies of porphyrin–flavonol hybrids **2** and **3** (Scheme 1B). 4-(Dimethylamino)benzaldehyde (**4**) and 4′-bromo-2′-hydroxyacetophenone **5** were converted to substituted flavonol **6** via the Algar–Flynn–Oyamada reaction,^35^ and the bromo substituent in **6** was used for installing an aldehyde group in **7** through a Pd-catalyzed formylation with CO/H_2_ (Scheme 2). The methoxymethyl (MOM) protection of the 3-hydroxyl group was necessary as this functional group strongly interfered with macrocyclization. In the next step, the porphyrin–flavonol hybrid **8** was obtained by condensing pyrrole and the aldehyde **7** using the well-established Lindsay conditions.^36^ Finally, one-pot methylation of the dimethylamino groups in **8** and concomitant MOM deprotection afforded the target molecule **2** on a gram scale. The use of column chromatography was necessary only after the formation of porphyrin **8** step (iv), while conventional purification methods (extraction and precipitation) were used during the other synthetic steps. Hybrid **3** was obtained on a multigram scale using the same strategy, and no chromatographic methods were required throughout this synthetic sequence. Detailed synthetic procedures are provided in the Supporting Information.

**Figure 1:**
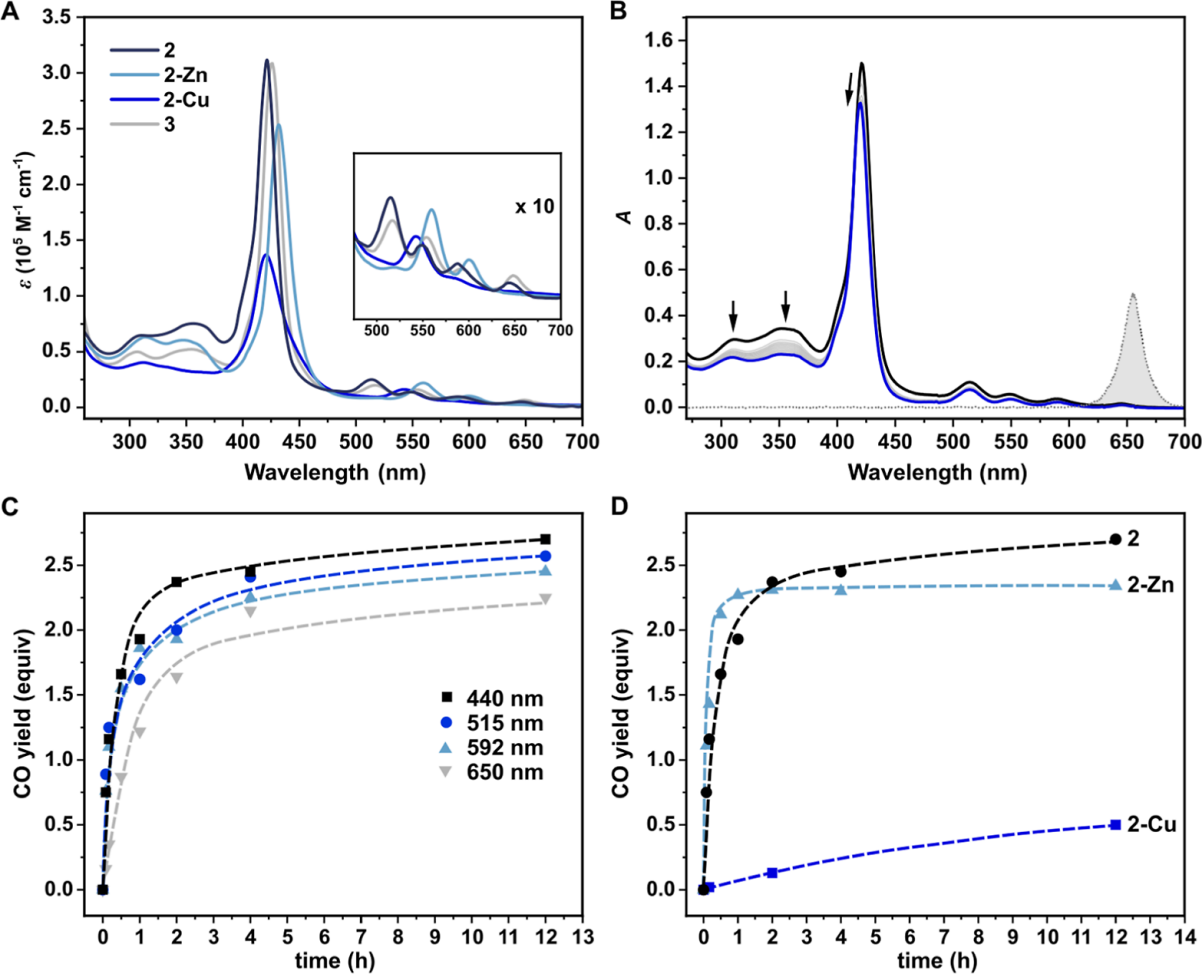
Photochemical properties of studied hybrids. (A) Absorption spectra of **2**, **2-Zn**, and **2-Cu** (methanol), and **3** (methanol/DMSO, 4:1, v/v). Inset: a 10-fold magnification of the Q-band region. (B) Irradiation of **2** in aerated methanol (5 μM) at 650 nm; the spectra were recorded every 60 min, and the spectra prior to (black line) and after (blue line) 12 h irradiation are highlighted. The emission spectrum of the irradiation source is shown as a gray dotted line. (C) Time-dependent CO evolution from **2** in aerated methanol (5 μM) upon irradiation at different wavelengths in the 0–12 h interval. (D) Time-dependent CO evolution in aerated methanol from **2**, **2-Zn**, and **2-Cu** upon irradiation at the Soret band in the 0–12 h interval. The yield of CO released was determined by headspace GC–MS and expressed as equivalents of CO released by 1 equiv of the photoCORM.

The absorption spectra of **2** and **3** are analogous to those of metal-free porphyrins^37^ and exhibit strong and sharp bands in the blue region (Soret bands), along with four less intense bands in the 500–670 nm range (Q bands) (Figure 1A, Table 1). Two additional bands at 310 and 360 nm correspond to the electronic transitions of the flavonol groups in their conjugate acid forms. Hybrids **2** and **3** emit in the red-to-NIR region (λ_em_ = 650, 715 nm; Figure S4B) with a moderate fluorescence quantum yield (Φ_f_) of 0.035. The absorption spectra of the hybrids resemble the sum of the absorption spectra of individual chromophores (Figure S4A).

**Table 1:** Photophysical and Photochemical Properties of the Porphyrin–Flavonol Hybrids^a^

cmpd	λ_abs_/nm (ε/10^4^ M^–^^1^ cm^–^^1^)^b^	λ_em_/nm	Φ_f_^c^	CO yield^d^/equiv	Φ_CO_^e^	Φ_CO_ε_max_^f^/M^–^^1^ cm^–^^1^ at (λ_abs_/nm)	Φ_Δ_^g^
**2**	421 (31.3), 514 (2.4), 549 (1.3), 590 (0.9), 645 (0.4)	649, 715	0.035	2.9 ± 0.1	0.018 ± 0.001	(5.6 ± 0.3) × 10^3^ (421)	0.17
				2.7 ± 0.1^h^			
**2-Zn**	431 (25.6), 560 (2.6), 601 (0.9)	611, 660	0.017	2.3 ± 0.1	0.040 ± 0.002	(1.0 ± 0.1) × 10^4^ (431)	0.1
				2.1 ± 0.1^h^			
**2-Cu**	421 (15.4), 543 (1.8), 582 (0.7)	ND	ND	0.8 ± 0.1	0.002 ± 0.001	(2.9 ± 0.8) × 10^2^ (421)	<10^–3^
**3**	426 (33.9), 517 (2.0), 554 (1.5), 591 (0.8), 650 (0.7)	652, 717	0.036	3.3 ± 0.1	0.003 ± 0.001	(1.0 ± 0.2) × 10^3^ (426)	0.32^i^
				2.1 ± 0.1^h^			

a All measurements were performed in methanol for **2**, **2-Zn**, **2-Cu**, and methanol/DMSO (4:1, v/v) for **3**, unless stated otherwise.

b Molar absorption coefficients ε.

c Fluorescence quantum yields Φ_f_ upon excitation at the Soret band.

d Total chemical yields of released CO monitored by GC headspace, obtained upon exhaustive irradiation at the Soret band.

e Absolute quantum yields of CO release determined using a Si-photodiode.

f CO uncaging cross sections Φ_CO_ε_max_ at (λ_max_).

g Singlet oxygen production quantum yields obtained from the ^1^O_2_ luminescence.

h In PBS.

i In DMF. ND = not detected.

Both compounds were found to be stable in solutions for days at room temperature in the dark (Figure S5). Irradiation of compound **2** or **3** (*c* ∼ 5 μM) at 650 nm in methanol led to a decrease in the intensity of the bands at 310 and 360 nm, accounting for the decomposition of the flavonols units, and a decrease in the porphyrin Soret band, most likely due to self-sensitized oxidation of the macrocycle.^38^ Analogous photochemical behavior was observed upon irradiation at 440, 515, and 600 nm (Figure S6). This consistent wavelength-independent behavior suggests that the entire visible range can be used for photoactivation.

Hybrid **2** irradiated in the range of 440–650 nm, liberated CO with maximum chemical yields of 2.9 ± 0.1 equiv in methanol and 2.7 ± 0.1 equiv in a phosphate buffer saline (PBS) solution. The yields of CO from **3** in methanol/DMSO (4:1, v/v) were higher (3.3 ± 0.1 equiv) but dropped to 2.1 ± 0.1 equiv in PBS. The time-dependent CO evolution in Figure 1C shows that **2** released most of CO in the first 30–60 min. The quantum yield of CO release (Φ_CO_) for **2** in methanol, 0.018 ± 0.001, was found to be wavelength-independent across the entire visible range (Table S6) and was orders of magnitude higher than the photodecomposition of the porphyrin core observed at the Soret band. Accounting for the high molar absorption coefficient of the porphyrin core, an uncaging cross-section (Φ_CO_ε_max_) of 5600 M^–1^ cm^–1^ at λ_max_ = 421 nm is extraordinarily high, one of the highest values reported to date for a visible-light-activatable photoCORM.^10^ An uncaging cross-section of 70 M^–1^ cm^–1^ was found at 645 nm (see all Φ_CO_ and Φ_CO_ε_max_ values in Table S6).

The ability of porphyrins to incorporate metal cations gave us a tool to control and tailor the photochemical properties of the hybrids for a given application. We optimized a general and facile procedure for incorporating metal cations by reacting **2** with the corresponding metal triflates in methanol or DMF. Analytically pure metal complexes **2-Zn** and **2-Cu** were obtained by precipitation from a crude reaction mixture by slow addition of CH_2_Cl_2_. Upon metal insertion, the absorption spectra were only slightly affected, possessing molar absorption coefficients lower by 20–50% compared to that of **2**. The emission properties are more influenced as Φ_f_ decreased for **2-Zn** by a factor of 2, while no emission was detected for **2-Cu**. We also examined whether these hybrids do not form flavonolato complexes among individual flavonol moieties and zinc or copper cations. The interpretation of the absorption, ^1^H NMR, and MS spectra confirmed that the flavonol group is not complexed (e.g., Figures S39 and S68).

The incorporation of metals significantly impacted the system’s ability to release CO (Figure 1D). Irradiation of **2-Zn** in methanol at 440 or 600 nm led to the efficient disappearance of absorption bands of both the flavonol and porphyrin moieties. A higher Φ_CO_ of 0.040 ± 0.002 (∼2.2 times higher than that of **2**) resulted in an extraordinarily high uncaging cross-section of 10 230 M^–1^ cm^–1^ at λ_max_ and 380 M^–1^ cm^–1^ at 601 nm. The yield of released CO was 2.3 ± 0.1 equiv in methanol and 2.1 ± 0.1 equiv in PBS. In contrast, **2-Cu** showed reduced photoreactivity with a much less efficient CO release (Φ_CO_ = 0.002 ± 0.001) and a lower maximum chemical yield of 0.8 ± 0.1 equiv upon exhaustive irradiation at 440 nm. In our recent study, the porphyrin-Cu(II) derivatives bearing a leaving group in the *meso*-methyl position were found to be photochemically inactive due to an ultrafast internal conversion observed as the main deactivation pathway.^29^

The products of the flavonol moiety degradation formed upon irradiation of **3** at 440 nm were identified as the anticipated esters of salicylic acid (MALDI-TOF; Figures S12 and S13). There was an evident sequential photodecarbonylation in all four flavonol units, but we were not able to assign the exact structures of products formed from porphyrin ring opening by MALDI-TOF and ESI-TOF. This degradation pathway, which is not relevant in the case of bimolecular flavonol sensitization mentioned above, is probably responsible for CO yields lower than 4 equiv in the studied hybrids (Table 1).

The essential role of oxygen^20^ in the solution was confirmed by irradiating **3** in a degassed methanol/DMSO solution (4:1, v/v). The photodecomposition of the flavonol groups (detected at 355 nm) was 15 times slower, and only traces of CO were detected, even after prolonged irradiation (Figures S9 and S10).

We examined the role of ^1^O_2_ produced by porphyrin sensitization, but we did not observe any correlation between the Φ_CO_ and Φ_Δ_ values (Table 1). In addition, the CO production from **3** was not affected in the presence of a singlet oxygen trap (NaN_3_ or α-terpinene, 5 mM; Table S7); thus, ^1^O_2_ is not directly involved in the reaction. On the other hand, the presence of a triplet quencher (cyclooctatetraene or 4-nitrobenzyl alcohol, 10 mM) reduced the CO release efficiency by ∼20% (Table S8), indicating a productive triplet-excited state.

To examine the CO release mechanism from the hybrids, we first ruled out possible charge transfer from the triplet porphyrin to the flavonol as the initial state of the process (Supporting Information). We then hypothesized that triplet-excited porphyrin (Por) sensitizes flavonol (Fla) according to
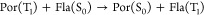


However, according to our calculations, this process is endergonic with a Gibbs free energy of 0.48 eV (calculated at the B3LYP/6-31+g* level; Scheme S2). This conclusion is also supported by the experimentally measured energetics: the triplet energies, *E*_T_, of porphyrin and Zn-porphyrin, 1.44 and 1.60 eV, respectively,^39^ are lower than that of flavonol, 1.79 eV.^40^ The process is thus energetically forbidden. However, it would be exergonic with a Gibbs free energy of −0.40 eV (see Scheme S2) if the acceptor were flavonol in its tautomeric form (experimental *E*_T_ = 0.99 eV,^40^) formed upon intramolecular proton transfer from the 3-OH group to the carbonyl group (tautomerization)
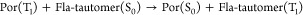


**Scheme 3:**
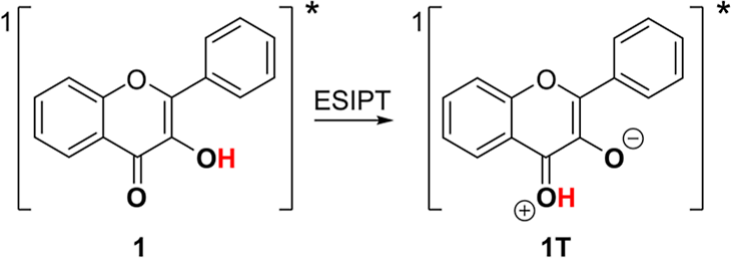
Phototautomerization of Flavonol

Such a tautomer is known to be generated rapidly upon excitation to the singlet excited state (<125 fs) via excited-state intramolecular proton transfer (ESIPT) to give a Stokes-shifted emitting phototautomer **1T** (Scheme 3).^41,42^

Considering the ground-state flavonol **1** as the initial state and tautomer **1T** in the triplet state T_1_ as the final state according to
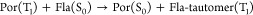
the Gibbs free energy of the TEnT process decreased to 0.06 eV (see Scheme S2) as a consequence of the flavonol energy stabilization upon tautomerization in the triplet state (see Figure S1). This intermolecular energy transfer should thus be governed by the flavonol ESIPT either stepwise or simultaneously.

Electronic transitions in many critical processes are coupled to a nuclear motion, with proton-coupled electron transfer (PCET) being the most prominent example.^43^ PCET plays an important role in many biological and chemical processes, such as proton-coupled redox processes occurring during tyrosine oxidation and reduction in photosystem II,^44^ redox reactions of tyrosine in biological energy transduction, charge transport or enzymatic catalysis,^45^ or in organic synthesis.^46^ At higher energies, the nonlocal Auger-Meitner processes are mediated by proton transfer in the intermediate state.^47^

In 2019, Hammarström, Mayer, Hammes-Schiffer, and co-workers provided experimental evidence of the existence of inverted region behavior for concerted proton–electron transfer.^48^ The same laboratories very recently reported a unique mechanism in anthracene–phenol–pyridine triads, in which energy transfer is coupled to a nuclear motion, termed proton-coupled energy transfer (PCEnT).^49^ In this process, the formation of a locally excited state on anthracene is followed by singlet–singlet energy transfer to the phenol–pyridine chromophore. Despite the lack of spectral overlap of the donor emission and acceptor absorption spectra required for the Förster resonance (dipole–dipole) mechanism,^50,51^ the process was made possible by coupling proton transfer in the phenol–pyridine unit with energy transfer that lowered its excited-state energy.

The question is whether such a mechanism would also be operational in the triplet electronic manifold as most antenna systems absorbing visible/NIR light exhibit a relatively efficient intersystem crossing (ISC) to produce longer-lived triplet states. Triplet energy transfer (TEnT) by electron exchange is a spin-allowed process in which the triplet energy of the donor should exceed that of the acceptor and requires appreciable overlap of their molecular orbitals.^52^

**Scheme 4:**
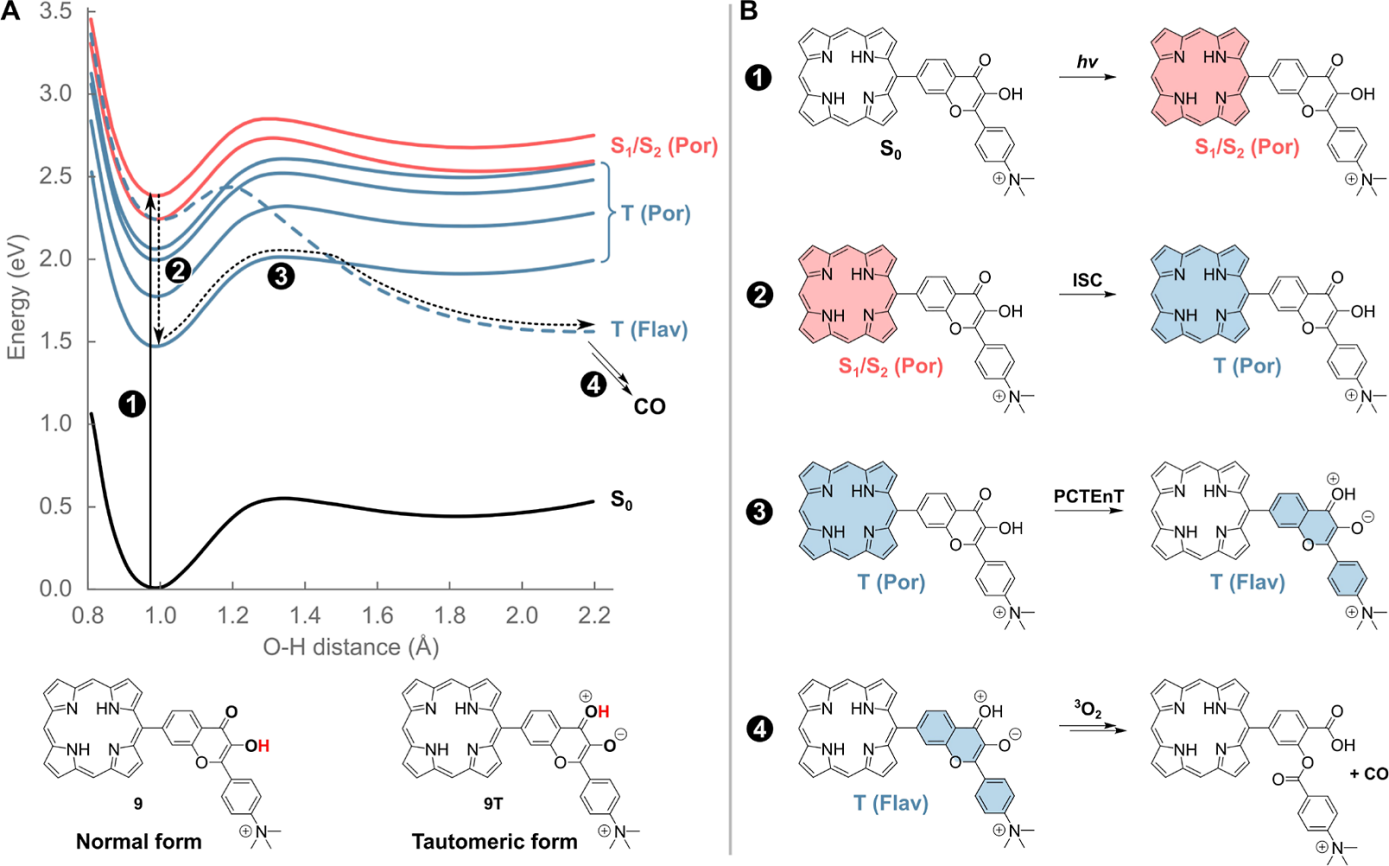
Proton-Coupled Triplet–Triplet Energy Transfer. (A) Potential Energy Curves and (B) the Proposed Mechanism of CO Photorelease from a Porphyrin (Por)-Flavonol (Flav) Model Hybrid 9 via Four Reaction Steps The potential energies are calculated at the TDDFT/B3LYP/6-31g* level. The red and blue colors correspond to the singlet (S) and triplet (T) excited states, respectively

In this work, we investigated the possibility that the mechanism of flavonol sensitization occurs through a simultaneous proton-coupled TEnT (PCTEnT). We conducted quantum-chemical calculations on model compound **9** (Scheme 4). Upon excitation into one of the absorption bands, a locally excited singlet state of porphyrin, corresponding to the S_1_ and S_2_ electronic states, is formed, while the flavonol unit remains in its nonproductive ground state (step 1, Scheme 4B). The singlet excitation localized on the flavonol chromophore and a mixed excitation between the porphyrin and flavonol units were found to be above the S_1_ and S_2_ energy levels (Table S3); thus, they do not interfere with the overall process. Analogous to the isolated porphyrin, efficient ISC to the triplet manifold and subsequent IC to the lowest triplet state^29^ follow (step 2, Scheme 4). The triplet state with the excitation on the flavonol units is located ∼0.8 eV above the lowest triplet state of porphyrin, and this electronic state is still not populated (note: electronic interactions between the two units are limited; the electronic states can be safely associated with excitations localized on the two different units; see Supporting Information and Figure S1C). Similar to the calculations on isolated flavonol, **1** (Figure S1A), tautomerization of flavonol via intramolecular ESIPT (step 3, Scheme 4) is the critical step in the transformation to produce tautomer **9T** with the triplet excitation localized on the flavonol unit, thus via the PCTEnT mechanism. Note that in the ground and first four triplet states of **9**, the potential energy curves follow that of the flavonol singlet state, and the energy increases upon proton transfer, whereas a higher flavonol triplet state (T_5_) possesses a minimum for the excited flavonol-tautomer. Therefore, simultaneous proton and energy transfers should occur in the lowest triplet state–the proton transfer mediates the energy transfer taking place at the crossing of the two diabatic states (with the triplet excitation localized either on the flavonol or porphyrin moieties). The tautomerization barrier in the triplet state (the calculated value of 0.53 eV in protic methanol; Figure S3 and Table S4) allows the process to occur within the triplet-state lifetime. The relative energies of both tautomers in the excited (triplet) state are within the thermal energy, with the Gibbs free energy of the tautomeric form being higher by only 0.16 eV (Table S5). The triplet-excited flavonol unit can then undergo the reaction with triplet oxygen to release CO^24,25^ (step 4, Scheme 4); the triplet state of flavonol is more acidic than that in the ground state, yet it should not deprotonate in methanol (see the Supporting Information). The amount of **9T** is possibly very small because it is formed slowly across the barrier, while it is consumed in the rapid reaction with triplet oxygen. The whole process can thus be entropically driven despite the slightly endothermic nature of the PCTEnT process. The whole system is similar to other endothermic photochemical processes.^53−55^

Directly confirming the PCTEnT process requires spectroscopic characterization of the flavonol tautomer in the triplet-excited porphyrin–flavonol complex. This is, however, very difficult, as the triplet tautomer is a reactive intermediate that undergoes a comparatively rapid decay, and its concentration must be very low. Furthermore, the TA signal of flavonol **1** appears in the 390–430 nm region (Figure S17 and the literature^43^); thus, it is located within the region of porphyrin ground-state bleaching, making its direct detection difficult due to the strong signal overlap.

Instead, we aimed to study triplet–triplet energy transfer by comparing the triplet lifetimes of hybrid **3** and model porphyrins under different conditions. Femtosecond (fs) TA spectra of **3** in DMSO (Figure S14) showed four main species upon excitation at 387 nm (at this wavelength, both flavonol moieties and the porphyrin core absorb). The first three components are assigned^39,40^ to an ultrafast internal conversion from S_2_ to S_1_ (0.2 ps) and vibrational relaxation within the Q bands (0.8 and 9.9 ps). The final component is associated^41,42^ with singlet–triplet ISC (*k*_ISC_ = 4.1 × 10^8^ s^–1^). We performed TA experiments with **3** and tetra-4-ethynylphenylporphyrin (TEPP) in THF to selectively excite the porphyrin core at 520 nm. TA spectra in the 300–1050 nm region highlight a fast and a slow component in both compounds (Figure S15). The faster component, similar for both **3** and TEPP (8.6 and 11.8 ns, respectively, and not affected by the presence of oxygen), is assigned to singlet–triplet ISC. The slower component was sensitive to the oxygen content, with lifetimes 3 orders of magnitude longer in degassed conditions. We assigned it to the triplet state. The triplet lifetime of hybrid **3** in degassed solutions (168 μs) was found to be shorter than that of TEPP by a factor of 2 (381 μs). This may suggest that a quenching process is operative. However, these data cannot be directly compared because the model porphyrin is not an exact model of the porphyrin core in **3**.

Nanosecond (ns) TA spectra in degassed THF were compared for **3** and tetraphenylporphyrin (TPP) upon excitation at 532 nm (Figure S16). The spectra of **3** revealed an intense band in the 300–600 nm region, split into two bands by the ground state bleach at 425 nm. Both bands decayed with the same lifetime of 90 μs and are assigned to the triplet state^43^ (τ = 292 ns in an aerated solution). nsTA spectra of TPP under the same conditions lacked the prominent TA band in the UV region (flavonol) and showed a longer triplet lifetime (120 μs) again.

Finally, another indirect evidence of the proposed TEnT mechanism was provided by Stern Volmer analysis of the ZnTcPP triplet decay at different triplet quencher **1** concentrations. A bimolecular quenching rate *k*_q_ of ∼2 × 10^5^ M^–1^ s^–1^ (Figure S18) was found, and it correlates well with the predicted, slightly endergonic Gibbs energy of the TEnT process (0.06 eV), calculated for the flavonol energy stabilization upon tautomerization in the triplet state.

Porphyrins are well-known for their ability to produce singlet oxygen (^1^O_2_) by ground-state oxygen (^3^O_2_) sensitization. A simultaneous presence of CO and ^1^O_2_ can be responsible for the synergistic enhancement of cytotoxicity of some PDT agents against cancer cells.^56^ We measured the efficiency of ^1^O_2_ production (Φ_Δ_) for all of the derivatives using the luminescence signal of ^1^O_2_. Φ_Δ_ of the metal-free hybrid **2** was found to be 0.17 in methanol. Metal insertion drastically reduced Φ_Δ_ to 0.10 for **2-Zn** and <10^–3^ for **2-Cu**.

**Figure 2:**
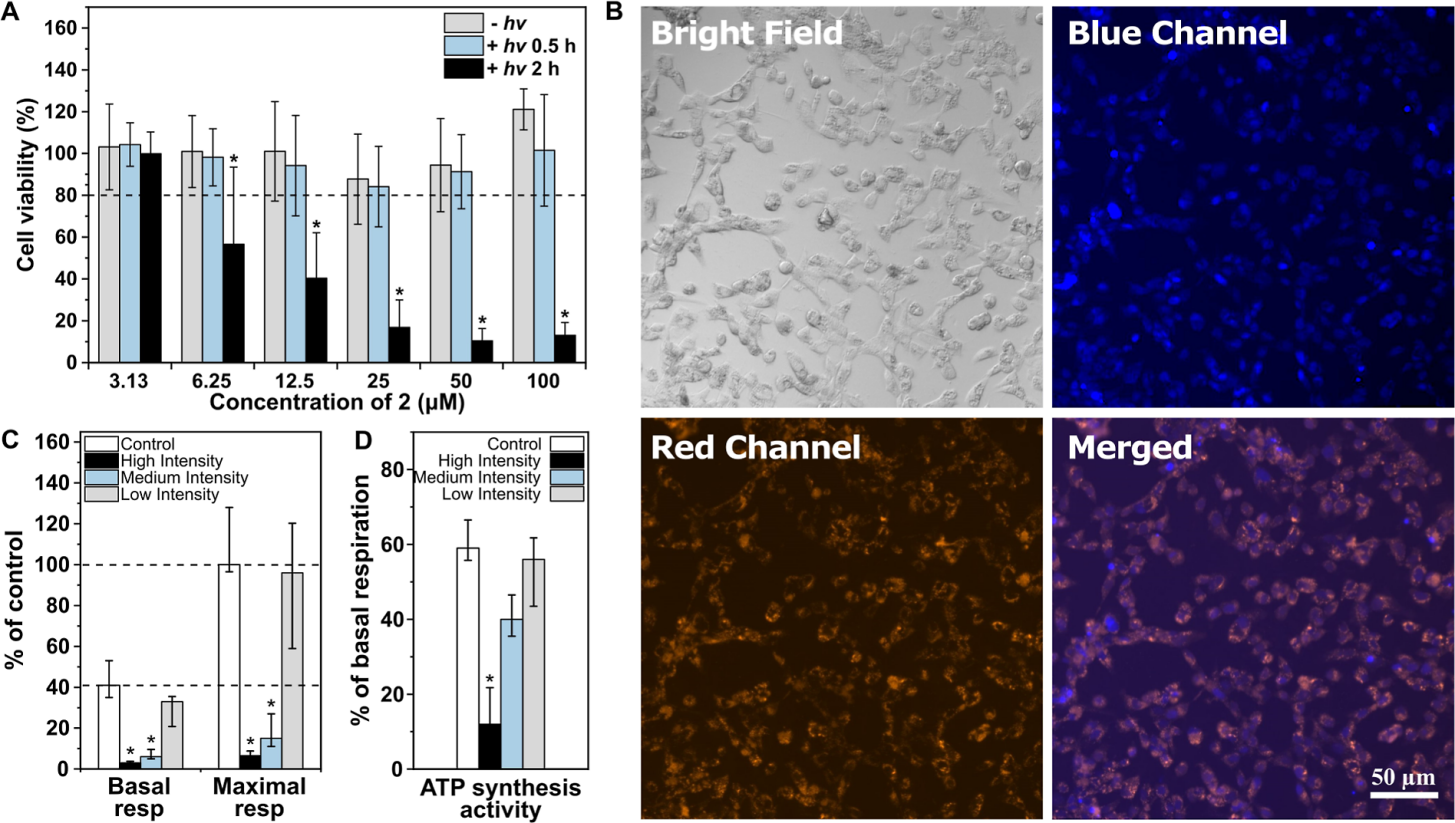
Biological experiments. (A) Effect of **2** on the viability of HepG2 cells after 24 h. Cytotoxicity was assessed by an MTT test after treating HepG2 cells with solutions of **2** in colorless MEM (1% DMSO) for 24 h without (-*hv*) or with (+*hv*) for 0.5 or 2 h of irradiation using white light (120 mW cm^–2^). The values are expressed as % of untreated controls. * *P*-value ≤ 0.05 vs untreated control; *n* ≥ 8. (B) Fluorescence microscopy of HepaG cells incubated with **2** (100 μM, 24 h). After incubation, the cells were washed with PBS and visualized by using white light (bright field) and a WIG filter (red channel). Nuclei were counterstained with DAPI and observed under UV light (blue channel). (C,D) Effect of irradiation intensity on the respiration of HepG2 cells. HepG2 cells were treated with **2** (25 μM) and irradiated with high-, medium-, or low-intensity white light (990, 120, or 8 mW cm^–2^, respectively) for 30 min; basal and maximum respiration (the values are expressed as % of the maximum respiration level of untreated controls) and ATP synthesis activities were analyzed immediately after incubation; ATP synthesis activity was calculated as the ratio of basal respiration to respiration after oligomycin inhibition and expressed as % of the basal respiration level of the untreated controls. * *P*-value ≤ 0.05; *n* ≥ 4.

We also evaluated the effect of compound **2** on cell viability. The human hepatoblastoma HepG2 cells were incubated with **2** within the concentration range of 1.5–100 μM. No significant toxicity was observed after 2 (Figure S20) and 24 h (Figure 2A) incubation in the dark. Stable photoproducts obtained upon prolonged irradiation of a solution of **2** did not affect cell viability up to the concentration of 200 μM following 24 h incubation (Figure S21). Similarly, no toxicity of **2** was observed upon irradiation of cells for 30 min, but after 2 h, it resulted in a significant decrease in cell viability at the concentration of 6.25 μM and higher (Figure 2A). We attribute the observed delayed cytotoxicity to ^1^O_2_ produced, thanks to oxygen photosensitization by primary photoproducts formed upon prolonged irradiation, as discussed above.

Mitochondria are an important target for CO as this gaseous signaling molecule can alter mitochondrial respiration and cytochrome c oxygenase activity or ROS production.^57^ Whether these effects are cytotoxic or cytoprotective depends mainly on the CO concentration.^58^ In our experimental system, HepG2 cells were incubated with **2** at a concentration of 25 μM and irradiated at different intensities of white light. It resulted in the CO release into the medium (Figures S22 and S23), which was manifested by differential effects on mitochondrial functions. The highest CO concentrations formed at high irradiation intensities strongly inhibited mitochondrial basal and maximum respiratory activities and ATP synthesis (Figure 2C,D). On the contrary, almost no effect on mitochondrial function was observed using low-intensity irradiation related to low CO concentrations. This demonstrates the high tuneability and adjustability of our light-triggered CO release systems, allowing for better CO release control with a predictable biological outcome. No effect on cellular respiration was observed in cells exposed to **2** in the dark or upon irradiation of solutions without the active substances (Figure S24). Even though CO can freely pass cellular membranes, some of its biological effects may depend on whether it is released intra- or extracellularly.^59^ Nevertheless, our fluorescence microscopy studies clearly showed the cytoplasmic localization of **2** (Figures 2B and S25) in human hepatic progenitor HeparG cells 24 h after incubation.

## Conclusions

In this work, we developed efficient photoactivatable carbon monoxide donors (photoCORMs) consisting of a porphyrin antenna and a covalently bound flavonol moiety as an energy acceptor. They absorb throughout the whole visible part of the spectrum, including the tissue-transparent window. Incorporating a metal cation into the porphyrin moiety can modulate the photochemical properties. With four CO flavonol donors attached to a single porphyrin chromophore, excellent CO release yields and uncaging cross sections, and in vitro biocompatibility, these photoCORMs have high potential for their further development and future biological and medical applications. Their advantages include high CO yields (atom economy), intracellular CO delivery, tuneability of CO release, and combining biological effects of CO with those of the porphyrin moiety. In this regard, it should be noted that photodynamic therapy using porphyrins^60^ has been established as an effective anticancer treatment for specific human tumors, and the same applies to the anticancer effects of CO.^61^

We hypothesized that the mechanism of CO release is based on proton-coupled triplet energy transfer (PCEnT) between the excited porphyrin and flavonol tautomer, analogous to the recently discovered process of proton-coupled singlet energy transfer.^49^ The viability of this mechanism was supported by our computational studies, but despite our efforts, none of our spectroscopic investigations provided direct evidence. Because the key intermediate (triplet-excited flavonol tautomer) formed during PCEnT is predicted to be energetically above the locally excited state, it may appear only at very low concentrations that time-resolved methods cannot detect. We will try to solve this problem in our future projects for which we will design new systems with longer-lived transients.

## Supporting Information Available

Materials and methods; synthesis; NMR, absorption, emission, and time-resolved spectroscopy; quantum-chemical calculations; and biological experiments (PDF)
