# Supplementary material for: Visible-Light-Activated Carbon Monoxide Release from Porphyrin–Flavonol Hybrids
Source: J Am Chem Soc. 2023 Dec 29;146(1):920–9. doi: 10.1021/jacs.3c11426 (PMC10785818; doi:10.1021/jacs.3c11426)
Supplement: Supplementary file 1 — ja3c11426_si_001.pdf [file ja3c11426_si_001.pdf]

SUPPORTING INFORMATION

Visible-Light-Activated Carbon Monoxide Release from Porphyrin-Flavonol Hybrids

Andrea Ramundo,[†, ‡] Jiří Janoš,[¶] Lucie Muchová,[§] Mária Šranková,[§] Jakub Dostál,[$] Miroslav Kloz,[$] Libor Vítek,[§] Petr Slavíček,[¶,]* Petr Klán[†,‡,]*

[†] Department of Chemistry, Faculty of Science, Masaryk University, Kamenice 5, 62500, Brno, Czech Republic.

[‡] RECETOX, Faculty of Science, Masaryk University, Kamenice 5, 62500, Brno, Czech Republic.

[¶] Department of Physical Chemistry, University of Chemistry and Technology, Technická 5, 16628 Prague 6, Czech Republic.

[§] Institute of Medical Biochemistry and Laboratory Diagnostics, and 4th Department of Internal Medicine, General University Hospital in Prague and 1st Faculty of Medicine, Charles University, Na Bojišti 3, 12108 Prague 2, Czech Republic.

[$] ELI Beamlines Facility, The Extreme Light Infrastructure ERIC, Za Radnicí 835, 25241 Dolní Břežany, Czech Republic.

* Petr.Slavicek@vscht.cz; klan@sci.muni.cz

Contents

Methods S3
- Materials and General Methods S3
- General Procedure for Irradiation in UV Cuvettes S3
- Determination of CO Yields S3
- Quantum Yield of CO Release (Φ_{CO}) S3
- Quantum Yield of $^1O_2$ Production (Φ_Δ) S4
- Transient Absorption Spectroscopy S4
- Cell Cultures S4
- Cytotoxicity Determination S5
- Determination of CO Concentration in Cell Medium S5
- High-Resolution Respirometry S5
- Fluorescence Microscopy S5
- Statistical Analysis S5

Bimolecular Sensitization Experiments S7
Quantum Chemical Calculations S8
Synthetic Procedures S28
Emission and Absorption Spectra S36
Stability in the Dark S37
Photoirradiation Experiments in Aerated Solutions S38
Photoirradiation Experiments in a Degassed Solution S39
Kinetic of the CO Evolution from **3** in a Degassed Solution S40
Photochemical Properties of **2**, **2-Zn**, **and 2-Cu** at Different Wavelengths S40
CO Production of **3** in Presence of a $^1O_2$ Trap S41
CO Production of **3** in Presence of a Triplet Quencher S41
Emission Spectra of the Irradiation Sources S42
Photoproducts Analyses S42
Transient Absorption Spectroscopy S46
$^1O_2$ Production of **2** After Irradiation S47
In Vitro Biological Studies S48
NMR Spectra S52
HRMS Spectra S71
References S77

Materials and General Methods

Reagents and solvents purchased from Sigma-Aldrich, Fluorochem, Fluka, and PorphyChem were used as received unless stated otherwise. Anhydrous solvents were obtained from ACROS Organics or dried using standard methods. Synthetic procedures were performed under an ambient atmosphere unless stated otherwise. Flash column chromatography was performed using silica gel (230−400 mesh). HPLC-UV/vis analyses were performed on an Agilent 1260 Infinity II HPLC system with a ZORBAX SB-Aq RR column (150 × 4.6 mm, 3.5 μm) using water (0.1% TFA)/CH_3CN gradient at a flow rate of 1.000 mL min^{-1}. NMR spectra were obtained on Bruker Avance II 300 MHz or Bruker Avance III 500 MHz spectrometers. ^1H and ^{13}C chemical shifts are reported in ppm relative to tetramethylsilane (δ = 0.00 ppm) using the residual solvent signals as internal references. ^{19}F chemical shifts are reported unreferenced. UV-vis spectra were obtained on an Agilent Cary 8454 UV-Vis spectrometer using matched 1.0 cm quartz cuvettes. Fluorescence was measured on an automated luminescence spectrometer in 1.0 cm quartz fluorescence cuvettes at 23 ± 1 °C; sample concentrations with absorbance below 0.1 at the excitation wavelength were used. Fluorescence quantum yields were determined using an integration sphere as the absolute value. The fluorescence quantum yield was measured for each sample five times, and the results were averaged. Photon fluxes were measured using a Thorlabs PM100D console equipped with a calibrated Si-photodiode. The exact masses were obtained using an Agilent 6224 Accurate-Mass TOF LC-MS (electrospray or atmospheric pressure chemical ionization) or a Bruker Daltonics UltrafleXtreme (MALDI-TOF/TOF) mass spectrometer in a positive or negative mode.

General Procedure for Irradiation in UV Cuvettes. A solution of the sample in the given solvent (3 mL) in a matched 1.0 cm quartz PTFE screw-cap cuvette equipped with a stirring bar was stirred and irradiated with a LED array. The progress of the photoreaction was monitored at the given time intervals by UV−vis spectroscopy using a diode-array spectrophotometer.

Determination of CO Yields. A solution of the sample in the given solvent (800 μL) in closed GC vials fitted with PTFE septa was irradiated with a LED array for the given times. The amount of released CO was quantified by a GC–headspace instrument (MXT-Msieve 5Å PLOT, 30 m, 0.53 mmID, 50 μm df, Restek column) equipped with a TIC/MS detector in a SIM mode, which was calibrated using the quantitative photoreaction of cyclopropenone photoCORM (20–400 μL, *c* ~ 50 μM, in methanol).[1] The CO yields are expressed as the total chemical yields (equivalents of CO released from 1 equiv of the photoCORM).

Quantum Yield of CO Release (Φ_{CO}). A solution of the sample in methanol or methanol/DMSO (4 : 1, *v/v*; 1000 μL, *c* = 5 μM) in a closed GC vial fitted with a PTFE septum was irradiated using a xenon lamp through a monochromator set to the corresponding wavelength. The light beam (~7 mm^2) was collimated through the bottom of the vial to a calibrated Si-photodiode. The total irradiation time was chosen to reach a <5% conversion. The photorelease quantum yield (Φ_{CO}) was obtained as an absolute number from the amount of CO released against the number of photons absorbed by the sample in the given period of time. The total amount of photons absorbed by the sample $\boldsymbol{n}_{\mathbf{p}}^{\mathrm{abs}}$ was determined according to:

$$n_{\mathrm{p}}^{\mathrm{abs}} = tq_0 \int_0^{\infty} (1 - 10^{-A(\lambda)})\, I_{\mathrm{norm}}^{\mathrm{em}}(\lambda) d\lambda$$

where t is the time, $A(\lambda)$ is the sample absorbance at the wavelength λ, $I_{\text{norm}}^{\text{em}}(\lambda)$ is the emission spectrum of the light source at the wavelength λ normalized to a unit area (m^2), and q_0 is the absolute photon flux calculated by:

$$q_0 = \frac{I}{N_A hc} \int_0^{\infty} \frac{\lambda I_{\text{norm}}^{\text{em}}(\lambda)}{R(\lambda)} d\lambda$$

where I (in A) is the current measured by a calibrated Si-photodiode when the light beam goes through a blank solution of the solvent, N_A is the Avogadro number, h is the Planck constant, c is the speed of light, λ is the wavelength, $I_{\text{norm}}^{\text{em}}(\lambda)$ is the emission spectrum of the light source at the wavelength λ normalized to a unit area (m^2), and R is a wavelength-dependent instrument responsivity (in A W^{-1}). The amount of CO released was determined by headspace GC-MS. 5 independent measurements were performed for each sample.

Quantum Yield of $^1O_2$ Production (Φ_Δ). A solution of the sample in methanol or dimethylformamide (DMF, 3000 μL) in a 1.0 cm quartz fluorescence cuvette was irradiated using a xenon lamp through a monochromator. The $^1O_2$ luminescence at 1276 nm was recorded, and the quantum yield of $^1O_2$ production ($\boldsymbol{\Phi_\Delta}$) was obtained using the equation.[2]

$$\Phi_\Delta = \Phi_\Delta^{\text{Ref}} \frac{S}{S^{\text{Ref}}} \times \frac{\alpha^{\text{Ref}}}{\alpha}$$

where Φ_Δ^{Ref} is the quantum yield of $^1O_2$ production from rose bengal used as a reference ($\Phi_\Delta^{\text{Ref}} = 0.79$,[3]) S is the integrated signal (1240–1310 nm) of the $^1O_2$ luminescence of the sample, and α is the number of photons absorbed at the irradiation wavelength.

Transient Absorption Spectroscopy. The nanosecond laser flash photolysis setup was operated in a 90° arrangement of the pump and probe beams. Laser pulses of ≤700 ps duration at 355 nm (160 mJ) and 532 nm (240 mJ) were obtained from an Nd:YAG laser. The laser beam was dispersed into a 40-mm long and 10-mm wide modified fluorescence cuvette held in a laying arrangement. An over-pulsed Xe arc lamp was used as the probe light source. Kinetic decay traces were recorded using a photomultiplier. Transient absorption spectra were obtained using an ICCD camera equipped with a spectrograph. Samples were degassed by three freeze-pump-thaw cycles. The measurements were performed at ambient temperature (20 ± 2 °C) in methanol, methanol/DMSO (4:1, v/v), or THF using the 355 or 532 nm excitation.

Femtosecond transient absorption was measured with the pump−super continuum probe technique using a Ti/Sa laser system (775 nm, pulse energy of 0.9 mJ, full width at half-maximum of 150 fs, operating frequency of 426 Hz). One part of the beam was fed into a non-collinear optical parametric amplifier (NOPA). The output at 387 nm was frequency-doubled by a β-barium borate (BBO) crystal to λ = 266 nm and, upon compression, elicited pump pulses of 1 μJ energy and <150 fs pulse width. The probe beam was generated by focusing the 775 nm beam in front of a CaF_2 plate with a 2 mm path length that produced a supercontinuum spanning a wavelength range of 270−690 nm. The pump and probe beams were focused on a 0.2 mm spot on the sample flowing in an optical cell with a thickness of 0.4 mm. The probe beam and a reference signal obtained by passing the solution next to the pump beam were spectrally dispersed and registered with two photodiode arrays (512 pixels). Transient absorption spectra were calculated from the ratio of the two beams. The pump−probe cross-correlation was <100 fs over the entire spectrum. The measurements were performed at ambient temperature (20 ± 2 °C). The data of the fs TA spectrum was collected until 3.1 ns. The global analysis was performed to get evolution-associated spectra (EAS).

Transient Absorption Spectroscopy 2. All experiments were conducted on a homebuilt set-up constructed around a femtosecond titanium sapphire amplifier femtopower (Spectra Physics), generating 4.2 mJ pulses of ~20 fs duration at a repetition rate of 1 kHz and Solstice amplifier (Spectra Physics) sharing the fs oscillator. Amplifiers were synchronized both by means of electronic triggering and optical delay of the seed prior to amplification. Transient absorption experiments were performed using two laser beams: pump and probe. The white light supercontinuum generated in an Argon-filled hollow core fiber (Savannah, Ultrafast Innovations) driven by the femtopower amplifier served as the probe. The pump beam was generated by NOPA (TOPAS, Light Conversion) driven by the Solstice amplifier. The central wavelength was set to 520 nm for the hybrid **3** samples and 290 nm for flavonol **1** excitation. The power was set up to be 10 nJ per pulse for exciting the hybrid **3**, 20 nJ per pulse for TEPP and TPP, and 30 nJ per pulse for flavonol **1**. The pump and probe beams were focused at the same spot in the experimental sample, and their relative polarization was set at the magic angle. Their respective delay was adjusted by combining an optomechanical delay line and electronic synchronization between the two amplifiers, which allowed to scan delays up to 0.5 ms with a femtosecond precision. Each beam was interrupted on a shot-to-shot basis by an optomechanical chopper to acquire all four possible pulse combinations (pumped, not-pumped, dark background, pump scattering). The spectrum of the probe beam transmitted through the sample was acquired by a home-built prism spectrometer utilizing a 1 kHz CCD camera (Entwicklungsbuero Stresing). To reduce the noise due to white light fluctuations, we used a second identical detector to acquire the reference spectrum of the probe replica, avoiding the sample, and we performed the correction as described in the literature.[4] The degassed sample was contained in an air-tight 1-mm-thick optical cell during the experiment, the position of which was randomly scanned to refresh the sample in the laser focus.

Cell Cultures. The human hepatoblastoma HepG2 cell line (ATCC, Manassas, VA, USA) or terminally differentiated hepatic cells derived from a human hepatic progenitor cell line HepaRG (Gibco, Waltham, MA, USA) were grown in supplemented minimum essential medium (MEM) or William's media in a humidified atmosphere containing 5% CO_2 at 37 °C according to the manufacturer's instructions.

Cytotoxicity Determination. HepG2 cells were seeded into 96-well plates and treated with compound **2** in the concentration range of 1.5−100 μM dissolved in MEM with 1% DMSO and irradiated with medium intensity light (LED; 120 mW cm^{-2}) for 0.5 or 2 h or kept in the dark for up to 24 h. After incubation, MTT (3-(4,5-dimethylthiazol-2-yl)-2,5-diphenyltetrazolium bromide) reduction assay was performed as described previously.[5-6]

Determination of the CO Concentration in Cell Medium. HepG2 cells were seeded in a Petri dish (d = 6 cm), treated with a solution of **2** (c = 25 μM), and irradiated with high-, medium-, or low-intensity white light (LED; 990, 120, or 8 mW cm^{-2}, respectively) or kept in the dark for 30 min. Control samples contained a medium without compound **2**. At 0, 10, and 30 min, 180 μL of the medium aliquot was injected into a septum-sealed glass vial purged with a CO-free nitrogen atmosphere containing 20 μL of 30% sulfosalicylic acid. CO released into the vial's headspace was measured by gas chromatography with a reducing gas analyzer (GC/RGA Peak Performer 1, Peak Laboratories, Mountain View, CA, USA) as previously described[7] and expressed as nmol of CO per L of the medium.

High-Resolution Respirometry. HepG2 cells were seeded in a Petri dish (d = 6 cm) and treated with a solution of **2** (c = 25 μM) for 30 min. The working solution of **2** was prepared by diluting a DMSO

stock solution (c = 20 mM) with a MEM medium. Samples were irradiated (at high, medium, or low intensities, as described above) or kept in the dark for the entire incubation time (30 min). After this treatment, cells were washed with Hank's solution, trypsinized, and resuspended in the original treatment solutions, and the final suspension was used for respiration measurement. To determine the cell respiration level, the oxygen consumption of living cells was measured using an Oxygraph-2k (Oroboros Instruments GmbH, Austria). Respiration was measured at 37 °C in 2 mL chambers and expressed as O_2 flux per 10^6 cells (pmol s^{-1} 10^{-6} cells); the cell count was measured on Countess automated cell counter, Invitrogen, USA, Massachusetts. After calibration of the instrument (air calibration, equilibration), we proceeded with the coupling-control protocol for living cells (SUIT-003) using oligomycin (c = 2.75 μM), rotenone (c = 0.2 μM), and antimycin A (c = 5 μM) as inhibitors, and FCCP (titrated by 1 μM steps up to the final concentration of 4−6 μM) as an uncoupler.[8-9] The obtained data were analyzed with the Datlab software (version 7.4.0.4, Oroboros Instruments).

Fluorescence Microscopy. HepaRG cells were seeded in a 48-well plate and incubated with **2** (c = 100 μM) for 24 h. Nuclei were counterstained with DAPI for 30 min (4′,6-diamidino-2-phenylindole, a blue-fluorescent nucleic acid stain, Sigma). After incubation, cells were washed with PBS and visualized using a fluorescence microscope (Olympus IX 51 with a mercury burner U-RFL-T, Olympus, Japan) with WIG and UV emission filters (red and blue channels).

Statistical Analysis. The data are expressed as the mean ± SD (cytotoxicity) or median ± interquartile range (respiration). Differences between variables were evaluated by Student's t-test or Mann-Whitney Rank Sum test depending on data normality. Differences were considered statistically significant at $p \leq 0.05$.

Bimolecular Sensitization Experiments

Table S1. Bimolecular Sensitization Experiments

flavonol[a]	porphyrin[b]	λ_{IRR} / nm[c]	CO yield / equiv[d]
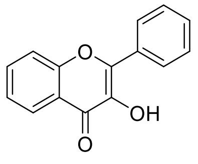	-	375	0.14 ± 0.01
	TcPP[e]	650	0.76 ± 0.01
**1**	ZnTcPP[f]	600	0.96 ± 0.01
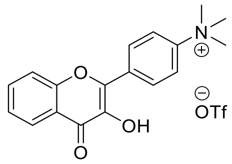	-	365	0.08 ± 0.01
**10**	ZnTcPP	600	0.54 ± 0.01
-	TcPP	515	N.D.
	ZnTcPP	600	N.D.

All measurements were performed in methanol. [a] c = 50 μM. [b] c = 10 μM. [c] The wavelength of irradiation. [d] The total CO yield, monitored by GC headspace, obtained upon exhaustive irradiation and expressed as equivalents of CO released by 1 equiv of the photoCORM. [e] TcPP = *meso*-(tetra-4-carboxyphenyl)porphyrin. [f] ZnTcPP = Zinc(II) *meso*-(tetra-4-carboxyphenyl)porphyrin. N.D. = not detected.

Table S2. Bimolecular Sensitization Experiments in Presence of a $^1O_2$ Trap.

flavonol[a]	porphyrin[b]	$^1O_2$ trap[c]	CO yield / equiv[d]
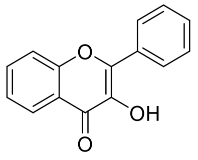	ZnTcPP[e]	-	0.37 ± 0.01
**1**		NaN_3	0.37 ± 0.03

All measurements were performed in methanol. [a] c = 50 μM. [b] c = 10 μM. [c] c = 10 mM. Irradiation at 600 nm (~2.5 mW cm^{-2}) for 2 h in the absence or presence of NaN_3 as a $^1O_2$ trap. [d] The total CO yield, monitored by GC headspace, obtained upon exhaustive irradiation and expressed as equivalents of CO released by 1 equiv of the photoCORM. [e] ZnTcPP = Zinc(II) *meso*-(tetra-4-carboxyphenyl)porphyrin.

Calculation of the Quantum Yield of Reaction between 1 and $^1O_2$

The reaction rate between flavonol **1** and $^1O_2$ produced by a sensitizer (Rose bengal; Φ_Δ = 0.79 in methanol[3]) can be expressed as:

$$k_r = -\frac{d[\mathbf{1}]}{dt} = \frac{dn(\mathbf{1})}{V\,dt} = k_\Sigma\,[^1O_2]\,[\mathbf{1}] \qquad \text{Eq. S1}$$

where k_r is the rate of decomposition, [**1**] is the concentration of **1**, n(**1**) is the amount of flavonol **1** in moles, V is the sample volume, k_Σ is the bimolecular reaction rate of **1** and singlet oxygen, and $[^1O_2]$ is the concentration of singlet oxygen.

The quantum yield of flavonol photooxygenation is defined by:

$$\Phi_{dec} = \frac{dn(\mathbf{1})}{q^0_{n,p}[1 - 10^{-A(\lambda)}]dt} \qquad \text{Eq. S2}$$

where $q^0_{n,p}$ is the incident photon flux, and $A(\lambda)$ is the absorbance of the sensitizer at the given wavelength. Combining Eq. S1 and Eq. S2 gives:

$$\Phi_{dec} = \frac{k_r[^1O_2]\,[\mathbf{1}]\,V}{q^0_{n,p}[1 - 10^{-A(\lambda)}]} \qquad \text{Eq. S3}$$

The production and deactivation of singlet oxygen is given by the following rate law:

$$\frac{d[^1O_2]}{dt} = \frac{\Phi_\Delta\, q^0_{n,p}[1 - 10^{-A(\lambda)}]}{V} - k_d\,[^1O_2] - k_\Sigma\,[^1O_2]\,[\mathbf{1}] \qquad \text{Eq. S4}$$

where Φ_Δ is the known quantum yield of singlet oxygen formation of rose bengal and k_d is the first-order rate constant of the quenching by the solvent. Applying steady-state approximation for $[^1O_2]$, we get:

$$[^1O_2] = \frac{q^0_{n,p}[1 - 10^{-A(\lambda)}]}{V} \times \frac{\Phi_\Delta}{(k_d + k_\Sigma[\mathbf{1}])} \qquad \text{Eq. S5}$$

Finally, plugging Eq. S5 to Eq. S3 gives:

$$\Phi_{dec} = \frac{k_\Sigma\,[\mathbf{1}]\,\Phi_\Delta}{k_d + k_\Sigma[\mathbf{1}]} \qquad \text{Eq. S6}$$

Employing the values used in the experiments ([**1**] = 50 μM; $k_\Sigma = 2.5 \times 10^5$ M^{-1} s^{-1}; $k_d = 9 \times 10^4$ s^{-1} and $\Phi_\Delta = 0.79$), the calculated values of $\Phi_{dec} = 1.0 \times 10^{-4}$ is obtained for **1**. Hence, the ratio of Φ_{CO} and Φ_{dec} is calculated (Eq. S7):

$$\frac{\Phi_{CO}}{\Phi_{dec}} = \frac{1.3 \times 10^{-3}}{1.0 \times 10^{-4}} = 13 \qquad \text{Eq. S7}$$

Quantum Chemical Calculations

Theoretical investigations of compounds **2** and **3** were performed on simplified models **9** and **11** with only one flavonol unit attached to the porphyrin core to lower the cost of calculations (Scheme S1). All computations were performed in methanol solvent, included via the polarizable continuum model. Excited states were acquired through the time-dependent density functional (TDDFT) formalism. All calculations were performed in the Gaussian 09, Revision D.01 package.[10]

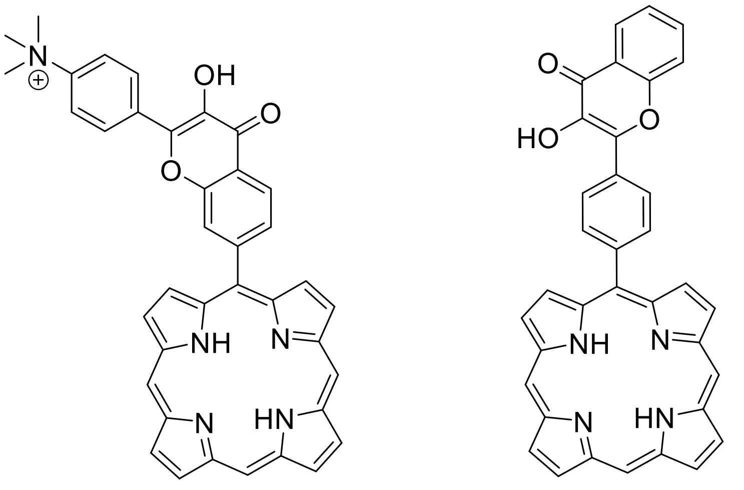

9 **11**

Scheme S1. Simplified models of structures **2** and **3** used in calculations. Structure **9** represents a model for compound **2**, and **11** represents a model for compound **3**.

Energy Transfer between Separate Triplet Excited Porphyrin and Singlet Flavonol. The energy transfer between separate porphyrin and flavonol chromophores was studied using the techniques of computational chemistry. Three possible processes were considered (Scheme S2). The first process is the triplet-triplet energy transfer (TEnT), i.e., the transfer of the excitation from porphyrin in its triplet state to the flavonol unit in the ground (singlet) state, resulting in the ground (singlet) state of porphyrin and triplet state of flavonol (*reaction 1*). This transfer is highly endergonic and unlikely to proceed in the solution. In the second process, we assume TEnT between porphyrin in the triplet state and flavonol tautomer. This process is highly exergonic (*reaction 2*). However, flavonol tautomer has a negligible population in solutions in its ground state (see the main text). Finally, the third proposed process assumes that flavonol undergoes tautomerization during TEnT, providing a slightly endergonic process (*reaction 3*). The triplet flavonol tautomer would then be in equilibrium with the population of about 9%, given by the Boltzmann distribution law, and we expect that the process can take place in the solution. Therefore, if we assume these two groups are connected as in compound **11**, the lowest triplet state would be localized either on porphyrin or flavonol in its tautomer form (**11T**).

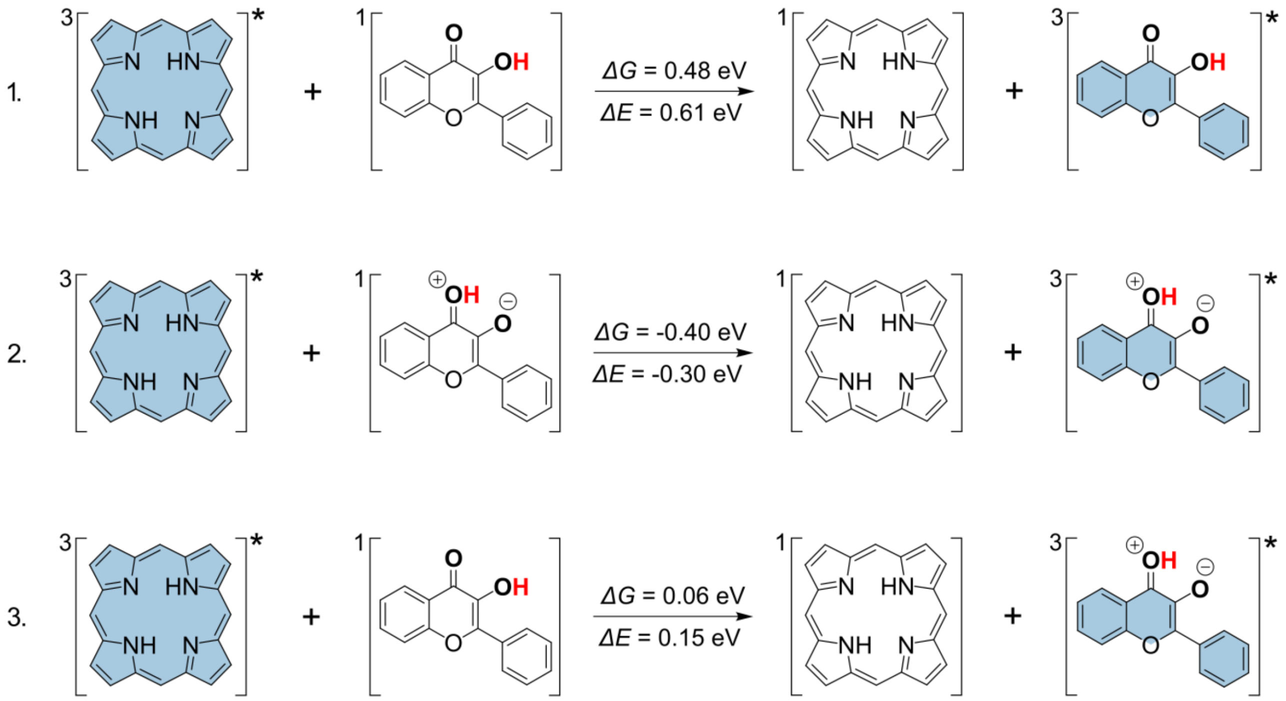

Scheme S2. Three possible processes of TEnT between triplet excited porphyrin and ground-state singlet flavonol or its tautomer form. The reaction energies and Gibbs free energies were calculated at the B3LYP/6-31+g* in the methanol solution.

To investigate the effect of the intramolecular proton transfer on the stabilization of the flavonol triplet state, we scanned the potential energy surface along the reaction coordinate (Figure S1). Figure S1A shows that the energy of the T_1 state decreases while that of the S_0 state increases. Thus, the gap between these states is reduced by about 1 eV. These findings explain the origin of proton-coupled TEnT (PCTEnT), a mechanism assumed to occur in porphyrin-flavonol hybrids. In the porphyrin-flavonol complex, the lowest triplet excitation is localized on the porphyrin unit. Therefore, tautomerization on the flavonol unit leads to a similar energy increase as observed for the isolated flavonol in its ground (singlet) state. The T_5 triplet state of the porphyrin-flavonol complex has the triplet excitation localized on the flavonol unit being stabilized by tautomerization. Once tautomerization is complete, the lowest triplet state of the complex has the excitation localized on the flavonol unit.

To demonstrate that the porphyrin and flavonol units in the hybrid behave as independent chromophores, we constructed the curves from Figure S1B with the properties of the independent units, see Figure S1C. We took the excitation energies of the isolated porphyrin and potential energy curves for the proton transfer in isolated flavonol and combined them. For all singlet excitation and triplet excitation located on porphyrin, we used the S0 proton transfer curves of flavonol. For the triplet excitation on flavonol, we used the T1 proton transfer potential energy curve. The minima of the curves were shifted by the porphyrin excitation energies. The resulting curves in Figure S1B show almost a quantitative correspondence to the complete calculation of the hybrid molecule. It demonstrates that the flavonol and porphyrin moieties in the hybrid behave as independent units. This is all in agreement with the spectral calculations and also with no conjugation between the π orbitals.

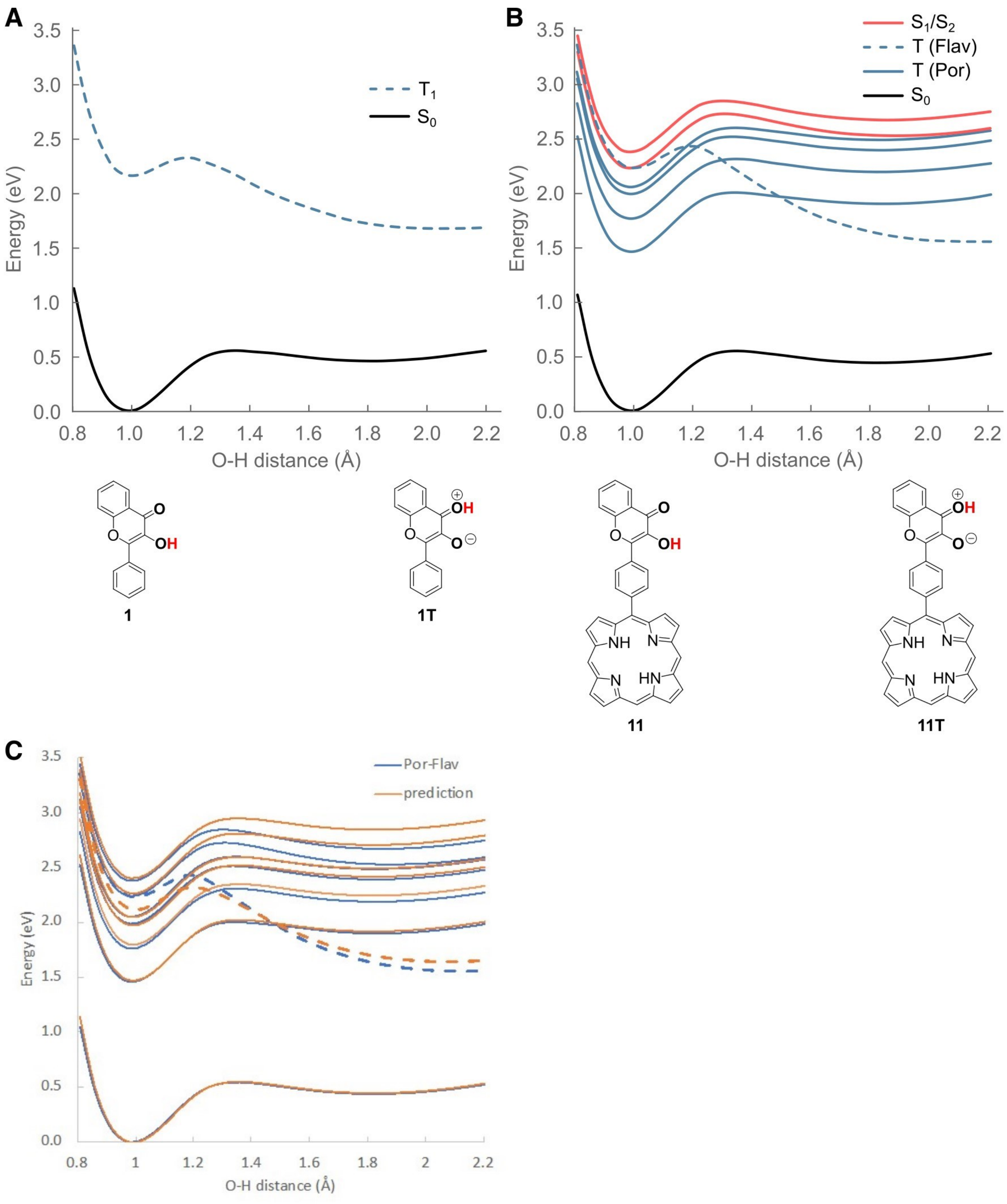

Figure S1. (A) Potential energy curves along tautomerization on flavonol **1** calculated at the B3LYP/6-31g* level. (B) Potential energy curves along the tautomerization coordinate in compound **11** calculated at the TDDFT/B3LYP/6-31g* level. (C) Potential energy curves of different electronic states in the porphyrin-flavonol complex predicted from the potential curves of isolated molecules (orange curves) compared with the calculations for the hybrid molecules (blue curves). A limited electronic interaction between the porphyrin and flavonol moieties is in good concordance.

Exploring the Acidity of the Flavonol Unit. We calculated the Gibbs free energy of the proton transfer into the methanol solution. We used the B3LYP/6-31+g* level of theory combined with PCM for methanol as a solvent, as in the previous calculations. The proton solvation energy was taken from the literature.[11] For pure flavonol, we calculated the singlet and triplet deprotonation Gibbs free energies to be 1.09 and 0.39 eV, respectively.

$FlavOH(S_0) \rightarrow FlavO^-(S_0) + H^+$ $\Delta G = 1.09$ eV

$FlavOH(T_1) \rightarrow FlavO^-(T_1) + H^+$ $\Delta G = 0.39$ eV

For the flavonol tautomer, the results are 0.63 and 0.81 eV.

FlavOH tautomer $(S_0) \rightarrow FlavO^-$ tautomer$(S_0) + H^+$ $\Delta G = 0.63$ eV

FlavOH tautomer $(T_1) \rightarrow FlavO^-$ tautomer$(T_1) + H^+$ $\Delta G = 0.81$ eV

All calculations suggest that the proton release to methanol is not favorable, yet we note that these calculations are based on implicit solvent modeling.

Charge Transfer Between Porphyrin and Flavonol. To rule out charge transfer as the first step of the mechanism, we optimized the porphyrin cation and flavonol anion and calculated the Gibbs free energy for the charge transfer between the separate units considering both the triplet and singlet porphyrin. Once again, as in the previous calculations, we used the B3LYP/6-31+g* level of theory combined with PCM for methanol as a solvent. Both Gibbs free energies are large and do not favor the charge transfer.

$Por(S_0) + Flav(S_0) \rightarrow Por^+(D_0) + Flav^-(D_0)$ $\Delta E = 2.7$ eV; $\Delta G = 2.6$ eV

$Por(T_1) + Flav(S_0) \rightarrow Por^+(D_0) + Flav^-(D_0)$ $\Delta E = 1.2$ eV; $\Delta G = 1.1$ eV

Nature of the Excitation in Compounds 9 and 11. The excited states were calculated in the ground state minimum at the TDDFT/CAM-B3LYP/6-31+g* level, and the results for structures **9** and **11** are shown in Table S3. For both compounds, the lowest four triplet states have the excitation localized on the porphyrin unit, and the excitation is localized on the flavonol unit only in the T_5 state. The T_5 energy approximately matches the energies of the S_1 and S_2 states (Q-bands; ≈2.2 eV) with the excitation localized on porphyrin. About 0.7 eV above the Q-bands, a dense manifold of triplet states appears. The Soret band (≈3.3 eV) correlates to the S_3 and S_4 states. We observe no mixed transition between porphyrin and flavonol moieties within the calculated energy range.

Table S3. Excited states of **9** and **11** calculated at the TDDFT/CAM-B3LYP/6-31+g* level in a methanol solution. Blue marks the transitions to the singlet states, while red the transitions to the triplet states. "Por" denotes the porphyrin moiety, "Flav" denotes the flavonol moiety. Oscillator strengths are presented only for the transitions with the same spin multiplicity.

compound 9				compound 11			
excitation	ΔE / eV	osc. strength	character of excitation	excitation	ΔE / eV	osc. strength	character of excitation
$S_0 \to T_1$	0.66	-	Por → Por	$S_0 \to T_1$	0.56	-	Por → Por
$S_0 \to T_2$	1.42	-	Por → Por	$S_0 \to T_2$	1.41	-	Por → Por
$S_0 \to T_3$	2.04	-	Por → Por	$S_0 \to T_3$	2.05	-	Por → Por
$S_0 \to T_4$	2.06	-	Por → Por	$S_0 \to T_4$	2.06	-	Por → Por
$S_0 \to S_1$	2.16	0.006	Por → Por	$S_0 \to S_1$	2.17	0.006	Por → Por
$S_0 \to T_5$	2.22	-	Flav → Flav	$S_0 \to T_5$	2.18	-	Flav → Flav
$S_0 \to S_2$	2.35	0.011	Por → Por	$S_0 \to S_2$	2.37	0.015	Por → Por
$S_0 \to T_6$	3.09	-	Por → Por	$S_0 \to T_6$	3.08	-	Por → Por
$S_0 \to T_7$	3.14	-	Por → Por	$S_0 \to T_7$	3.13	-	Por → Por
$S_0 \to T_8$	3.21	-	Flav → Flav	$S_0 \to T_8$	3.27	-	Flav → Flav
$S_0 \to T_9$	3.29	-	Flav → Flav	$S_0 \to S_3$	3.33	2.274	Por → Por
$S_0 \to S_3$	3.31	1.920	Por → Por	$S_0 \to T_9$	3.39	-	Flav → Flav
$S_0 \to S_4$	3.37	1.280	Por → Por	$S_0 \to S_4$	3.43	1.319	Por → Por
$S_0 \to T_{10}$	3.46	-	Por → Por	$S_0 \to T_{10}$	3.45	-	Por → Por
$S_0 \to T_{11}$	3.53	-	Por → Por	$S_0 \to S_5$	3.82	0.141	Flav → Flav

Proton-Coupled TEnT (PCTEnT). The excited state intramolecular proton transfer (ESIPT) is the key step for the triplet excitation transfer between porphyrin and flavonol subunits. For both models **9** and **11**, the proton transfer (tautomerization) coordinate was optimized in the ground state. The excited states were calculated along this coordinate (see Figure S2). Contrary to the main text, we present the picture in the adiabatic representation, i.e., we do not assign the calculated curves based on their character (diabatic representation) but rather according to the energy. We see that the T_1 state curve exhibits a two-minimum character. The minimum along the O–H distance of 1 Å has a character of porphyrin-localized excitation, whereas the minimum corresponding to the tautomer (around 2 Å) has a character of flavonol excitation. The barrier between these two minima is the barrier to energy transfer between porphyrin and flavonol.

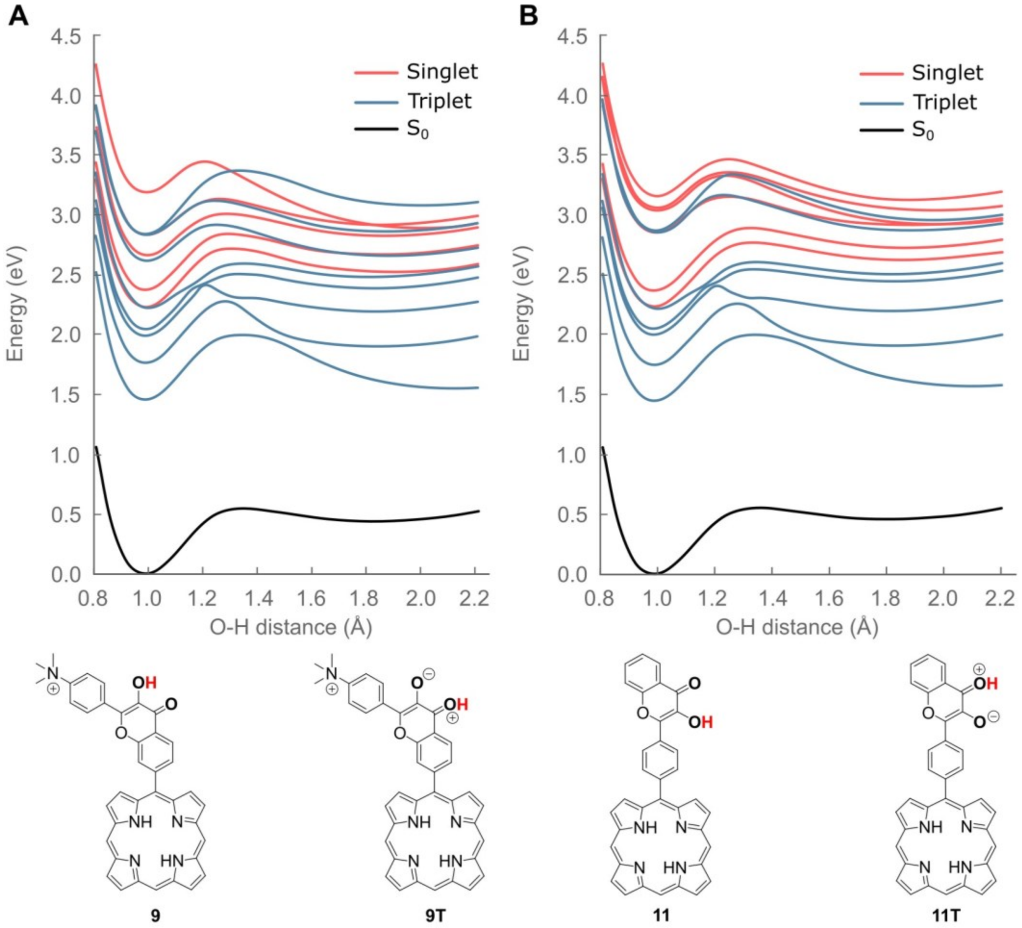

Figure S2. The potential energy curves along the proton transfer (tautomerization) coordinate for model compounds **9** and **11**. The coordinate was optimized in the ground state at the B3LYP/6-31g* level in the methanol solvent. The excited states were calculated with the TDDFT formalism.

To get a better estimate of the barrier, the T_1 state was optimized along the proton transfer coordinate (Figure S3), and the activation barrier was estimated from these scans (Table S4). The activation energies range from 0.53 to 0.58 eV and the activation Gibbs free energies are about 0.45–0.48 eV.

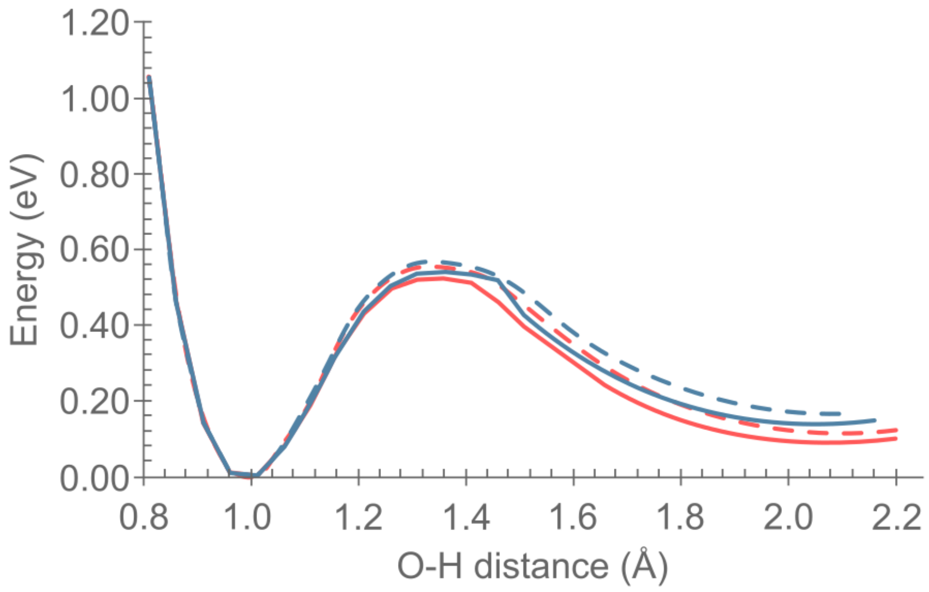

Figure S3. The T_1 potential energy curves along the proton transfer coordinate in model compounds **9** (red) and **11** (blue) calculated at the B3LYP level with 6-31g* (solid) and 6-31+g* (dashed) basis sets.

Table S4. Activation barrier derived from optimized scans in Figure S3 at the B3LYP level. The Gibbs free energy stems from the frequency analysis.

structure	basis set	ΔE / eV	ΔG / eV
**9**	6-31g*	0.53	0.45
	6-31+g*	0.56	0.48
**11**	6-31g*	0.55	0.46
	6-31+g*	0.58	0.48

Δ*G* and Δ*E* for Tautomerization in the T_1 State. The energy of tautomerization in T_1 was computed with different density functionals (Table S5). The more HF exchange is included in the hybrid functional, the higher the energy. The tautomerization energies range in the interval of 0.04–0.26 eV for **9** and 0.15–0.31 eV for **11**. The Gibbs free energies range in the interval of 0.07–0.32 eV for **9** and 0.18–0.30 eV for **11**.

Table S5. Tautomerization energy of **9** and **11** in methanol calculated with the 6-31+g* basis set with different functionals.

**method**	**basis set**	**9**		**11**	
		ΔE / eV	ΔG / eV	ΔE / eV	ΔG / eV
PBE	6-31+g*	0.043	0.072	-	-
B3LYP	6-31g*	0.095	0.139	0.147	0.175
B3LYP	6-31+g*	0.123	0.157	0.176	0.200
PBE0	6-31+g*	0.129	0.160	0.185	0.213
BMK	6-31+g*	0.224	0.318	0.278	0.265
BHandHLYP	6-31+g*	0.261	0.268	0.310	0.304

XYZ Coordinates of Optimized Structures

9 S_0 minimum, MeOH, B3LYP/6-31g*, E = −1965.374365 a.u.

C	-5.101216	1.059689	0.530567
C	-5.513257	-0.189487	0.035473
C	-6.895115	-0.458548	-0.011361
C	-7.819271	0.485933	0.418861
C	-7.385747	1.721322	0.905480
C	-6.024006	2.009785	0.961955
C	-4.512371	-1.165405	-0.413020
O	-3.229593	-0.710739	-0.241031
C	-2.147795	-1.455357	-0.593687
C	-2.278955	-2.736383	-1.152160
C	-3.612066	-3.270761	-1.361548
C	-4.720081	-2.404489	-0.955612
C	-1.108727	-3.446798	-1.485865
C	0.135602	-2.889532	-1.264279
C	0.260812	-1.595415	-0.697869
C	-0.890819	-0.884120	-0.365023
C	1.612495	-0.999279	-0.467719
C	1.929575	0.165565	-1.190333
N	3.116013	0.856393	-1.099916
C	3.120855	1.932825	-1.956574
C	1.855553	1.924450	-2.629373
C	1.134591	0.851690	-2.172332
C	4.154088	2.849128	-2.146367
C	5.414018	2.870592	-1.536867
C	6.421068	3.889747	-1.818556
C	7.499341	3.578268	-1.054557
C	7.139181	2.372053	-0.316783
N	5.871338	1.965573	-0.625224
C	7.994388	1.725148	0.583513
C	7.700964	0.571071	1.308271
N	6.512121	-0.114607	1.241699
C	6.528984	-1.198056	2.083582
C	7.815165	-1.201476	2.721926
C	8.527571	-0.127232	2.250239
C	5.485319	-2.101211	2.275662
C	4.223081	-2.110571	1.669273
N	3.759589	-1.229754	0.731277
C	2.486286	-1.637635	0.444705
C	2.126450	-2.812316	1.237929
C	3.212959	-3.107269	1.994917
O	-3.883922	-4.383314	-1.847899
O	-5.942669	-2.939473	-1.167617
H	8.978536	2.156554	0.738377
H	3.879310	0.574974	-0.491850
H	0.146485	0.549722	-2.485975
H	1.557587	2.650695	-3.373826
H	8.448601	4.095087	-0.983871
H	6.298236	4.717053	-2.506818
H	9.531982	0.171008	2.519812
H	8.132368	-1.939603	3.446543

H	5.740242	0.162824	0.642945
H	3.333512	-3.909134	2.712968
H	1.174628	-3.324050	1.219664
H	5.680586	-2.892772	2.993262
H	3.946830	3.637277	-2.864450
H	-0.832868	0.102733	0.081161
H	-1.212491	-4.433741	-1.924480
H	1.032712	-3.436789	-1.534015
H	-5.741050	-3.821613	-1.563257
H	-7.253647	-1.407527	-0.384041
H	-8.871658	0.233056	0.363912
N	-8.422018	2.717325	1.358938
H	-5.648012	2.953825	1.331292
H	-4.047290	1.301031	0.581174
C	-7.816294	4.007688	1.857499
C	-9.337412	3.060111	0.198653
C	-9.234887	2.122592	2.493915
H	-8.635757	4.661929	2.151542
H	-7.183178	3.799838	2.718354
H	-7.245686	4.470235	1.054149
H	-9.962574	2.865919	2.819807
H	-9.747091	1.230158	2.140999
H	-8.552524	1.871697	3.305506
H	-10.072317	3.786399	0.545573
H	-8.729036	3.481564	-0.601116
H	-9.838241	2.157420	-0.143599

9 T_1 minimum, MeOH, B3LYP/6-31g*, E = −1965.318599 a.u.

C	-5.095310	1.045006	0.525729
C	-5.511863	-0.209154	0.047059
C	-6.895287	-0.468976	-0.007957
C	-7.816268	0.488701	0.399137
C	-7.378202	1.728202	0.871028
C	-6.014948	2.007970	0.934567
C	-4.514011	-1.198490	-0.377412
O	-3.230056	-0.737697	-0.230232
C	-2.150069	-1.494542	-0.563882
C	-2.285901	-2.794935	-1.076229
C	-3.619264	-3.335560	-1.259173
C	-4.724719	-2.455550	-0.875828
C	-1.116726	-3.516366	-1.391258
C	0.128497	-2.955355	-1.191232
C	0.260758	-1.640180	-0.670470
C	-0.893693	-0.915186	-0.364777
C	1.604659	-1.042519	-0.462848
C	1.869998	0.206275	-1.132714
N	3.034178	0.927681	-0.999875
C	2.991059	2.052778	-1.790064
C	1.750616	2.052344	-2.448564
C	1.055930	0.904388	-2.051052
C	4.024940	3.032111	-1.926707
C	5.289715	3.055136	-1.373484

C	6.262396	4.121628	-1.604320
C	7.376884	3.781943	-0.913707
C	7.085055	2.505077	-0.260594
N	5.818723	2.083273	-0.546556
C	7.992018	1.808768	0.544098
C	7.762171	0.566341	1.198333
N	6.585095	-0.134749	1.116202
C	6.662378	-1.281346	1.867498
C	7.945211	-1.314618	2.449182
C	8.630984	-0.168191	2.033881
C	5.610213	-2.228903	2.030394
C	4.338657	-2.238516	1.491113
N	3.805345	-1.305773	0.612440
C	2.533042	-1.723913	0.368411
C	2.231288	-2.947149	1.119848
C	3.356000	-3.265535	1.803375
O	-3.894884	-4.464835	-1.704392
O	-5.948205	-2.998412	-1.059875
H	8.971926	2.251314	0.694053
H	3.814532	0.628912	-0.423579
H	0.080483	0.589600	-2.389186
H	1.419262	2.811509	-3.144207
H	8.313812	4.320194	-0.839584
H	6.090819	4.998540	-2.216443
H	9.638501	0.127564	2.293025
H	8.307435	-2.098710	3.100510
H	5.787202	0.173607	0.569542
H	3.519885	-4.102173	2.471338
H	1.285926	-3.470020	1.128010
H	5.853694	-3.058164	2.689295
H	3.764366	3.864602	-2.574808
H	-0.835500	0.087717	0.043038
H	-1.222442	-4.516165	-1.799175
H	1.021941	-3.512754	-1.451263
H	-5.748156	-3.893819	-1.425762
H	-7.257454	-1.420782	-0.369765
H	-8.870023	0.243191	0.337444
N	-8.411188	2.739104	1.298152
H	-5.635301	2.955061	1.292099
H	-4.040206	1.279921	0.581361
C	-7.799695	4.029153	1.790336
C	-9.307241	3.076102	0.121006
C	-9.246333	2.166538	2.428400
H	-8.616403	4.692942	2.070163
H	-7.176670	3.823895	2.659198
H	-7.217218	4.479439	0.988580
H	-9.970981	2.921311	2.733982
H	-9.762523	1.274966	2.079241
H	-8.578622	1.919216	3.253069
H	-10.040154	3.813242	0.448822
H	-8.684265	3.482238	-0.675481
H	-9.811427	2.173549	-0.216810

9T T_1 minimum, MeOH, B3LYP/6-31g*, E = −1965.318599 a.u.

C	-5.095310	1.045006	0.525729
C	-5.511863	-0.209154	0.047059
C	-6.895287	-0.468976	-0.007957
C	-7.816268	0.488701	0.399137
C	-7.378202	1.728202	0.871028
C	-6.014948	2.007970	0.934567
C	-4.514011	-1.198490	-0.377412
O	-3.230056	-0.737697	-0.230232
C	-2.150069	-1.494542	-0.563882
C	-2.285901	-2.794935	-1.076229
C	-3.619264	-3.335560	-1.259173
C	-4.724719	-2.455550	-0.875828
C	-1.116726	-3.516366	-1.391258
C	0.128497	-2.955355	-1.191232
C	0.260758	-1.640180	-0.670470
C	-0.893693	-0.915186	-0.364777
C	1.604659	-1.042519	-0.462848
C	1.869998	0.206275	-1.132714
N	3.034178	0.927681	-0.999875
C	2.991059	2.052778	-1.790064
C	1.750616	2.052344	-2.448564
C	1.055930	0.904388	-2.051052
C	4.024940	3.032111	-1.926707
C	5.289715	3.055136	-1.373484
C	6.262396	4.121628	-1.604320
C	7.376884	3.781943	-0.913707
C	7.085055	2.505077	-0.260594
N	5.818723	2.083273	-0.546556
C	7.992018	1.808768	0.544098
C	7.762171	0.566341	1.198333
N	6.585095	-0.134749	1.116202
C	6.662378	-1.281346	1.867498
C	7.945211	-1.314618	2.449182
C	8.630984	-0.168191	2.033881
C	5.610213	-2.228903	2.030394
C	4.338657	-2.238516	1.491113
N	3.805345	-1.305773	0.612440
C	2.533042	-1.723913	0.368411
C	2.231288	-2.947149	1.119848
C	3.356000	-3.265535	1.803375
O	-3.894884	-4.464835	-1.704392
O	-5.948205	-2.998412	-1.059875
H	8.971926	2.251314	0.694053
H	3.814532	0.628912	-0.423579
H	0.080483	0.589600	-2.389186
H	1.419262	2.811509	-3.144207
H	8.313812	4.320194	-0.839584
H	6.090819	4.998540	-2.216443
H	9.638501	0.127564	2.293025
H	8.307435	-2.098710	3.100510
H	5.787202	0.173607	0.569542
H	3.519885	-4.102173	2.471338

H	1.285926	-3.470020	1.128010
H	5.853694	-3.058164	2.689295
H	3.764366	3.864602	-2.574808
H	-0.835500	0.087717	0.043038
H	-1.222442	-4.516165	-1.799175
H	1.021941	-3.512754	-1.451263
H	-5.748156	-3.893819	-1.425762
H	-7.257454	-1.420782	-0.369765
H	-8.870023	0.243191	0.337444
N	-8.411188	2.739104	1.298152
H	-5.635301	2.955061	1.292099
H	-4.040206	1.279921	0.581361
C	-7.799695	4.029153	1.790336
C	-9.307241	3.076102	0.121006
C	-9.246333	2.166538	2.428400
H	-8.616403	4.692942	2.070163
H	-7.176670	3.823895	2.659198
H	-7.217218	4.479439	0.988580
H	-9.970981	2.921311	2.733982
H	-9.762523	1.274966	2.079241
H	-8.578622	1.919216	3.253069
H	-10.040154	3.813242	0.448822
H	-8.684265	3.482238	-0.675481
H	-9.811427	2.173549	-0.216810

11 S_0 minimum, MeOH, B3LYP/6-31g*, E = −1791.660207 a.u.

C	-1.665793	3.377321	0.669129
C	-2.935520	2.700450	0.447615
N	-2.746495	1.367370	0.210386
C	-1.393287	1.173111	0.269528
C	-0.702643	2.427025	0.566296
C	-4.161004	3.377886	0.495380
C	-5.443790	2.856560	0.343189
N	-5.757922	1.539986	0.114467
C	-7.119325	1.371578	0.032909
C	-7.706443	2.667799	0.216463
C	-6.688977	3.569337	0.405744
C	-7.757287	0.150921	-0.181166
C	-7.127265	-1.089637	-0.341141
C	-7.842390	-2.340743	-0.570029
C	-6.892278	-3.304724	-0.678286
C	-5.607364	-2.631127	-0.512723
N	-5.776505	-1.293077	-0.308936
C	-4.379281	-3.298980	-0.569953
C	-3.098760	-2.764402	-0.429279
N	-2.772209	-1.452476	-0.176074
C	-1.406483	-1.290949	-0.118972
C	-0.835109	-2.588208	-0.355220
C	-1.861588	-3.479445	-0.535735
C	-0.738927	-0.071146	0.100965
C	0.753211	-0.113452	0.154594
C	1.532931	0.581514	-0.785256
C	2.921483	0.539675	-0.740535

C	3.592695	-0.202205	0.252824
C	2.813269	-0.899818	1.197520
C	1.423602	-0.852683	1.142653
C	5.059743	-0.228493	0.278953
O	5.613289	0.534627	-0.718463
C	6.958212	0.654060	-0.879779
C	7.867714	0.000930	-0.032009
C	7.341590	-0.823267	1.042432
C	5.887601	-0.895776	1.143300
C	7.400487	1.462669	-1.934981
C	8.764777	1.612223	-2.134485
C	9.694823	0.966045	-1.295624
C	9.249173	0.169150	-0.256039
O	5.446096	-1.676396	2.159089
O	8.023335	-1.461327	1.868470
H	-8.841818	0.172816	-0.225316
H	-3.427223	-0.683965	-0.066630
H	0.223719	-2.797113	-0.388405
H	-1.786816	-4.539900	-0.736744
H	-8.918637	-2.443571	-0.636623
H	-7.024646	-4.365698	-0.852061
H	-8.770450	2.863287	0.204951
H	-6.772045	4.634451	0.576646
H	-5.095445	0.776410	0.016906
H	-1.548184	4.432367	0.884446
H	0.364179	2.548790	0.687846
H	-4.112368	4.446509	0.683390
H	-4.415362	-4.368800	-0.753976
H	1.042994	1.152617	-1.568197
H	3.492267	1.081216	-1.484985
H	3.293684	-1.473654	1.977978
H	0.847131	-1.389338	1.890390
H	9.117381	2.236934	-2.949669
H	10.758606	1.095722	-1.468118
H	6.671502	1.953112	-2.571399
H	9.942160	-0.340536	0.405448
H	6.282766	-1.998694	2.572761

11 T_1 minimum, MeOH, B3LYP/6-31g*, E = −1791.605008 a.u.

C	-1.845870	3.465068	-0.590353
C	-3.080454	2.708740	-0.430464
N	-2.804474	1.381061	-0.148919
C	-1.442900	1.288994	-0.123124
C	-0.828835	2.589684	-0.413023
C	-4.331214	3.282877	-0.565100
C	-5.623222	2.682925	-0.495111
N	-5.865078	1.347119	-0.290542
C	-7.215552	1.100193	-0.312272
C	-7.864093	2.332506	-0.532654
C	-6.872812	3.314869	-0.647382
C	-7.786594	-0.194930	-0.149843
C	-7.093000	-1.389242	0.044982

C	-7.730394	-2.695927	0.209467
C	-6.728741	-3.590305	0.386839
C	-5.479714	-2.831648	0.330020
N	-5.730244	-1.494443	0.116849
C	-4.227110	-3.397525	0.489580
C	-2.940882	-2.775977	0.469276
N	-2.678391	-1.451009	0.204621
C	-1.328096	-1.204543	0.293985
C	-0.708169	-2.424789	0.634906
C	-1.709070	-3.398435	0.732303
C	-0.718010	0.092127	0.101320
C	0.762622	0.142444	0.144660
C	1.544893	-0.679681	-0.690353
C	2.931586	-0.626123	-0.660988
C	3.606673	0.245732	0.219055
C	2.828727	1.061349	1.066547
C	1.440576	1.008731	1.023087
C	5.071523	0.279786	0.233837
O	5.625374	-0.685779	-0.568943
C	6.970495	-0.826675	-0.711549
C	7.879203	0.009018	-0.041784
C	7.352680	1.050753	0.823278
C	5.898779	1.132002	0.918655
C	7.413650	-1.849775	-1.559814
C	8.778006	-2.029198	-1.732876
C	9.707195	-1.201615	-1.071257
C	9.260681	-0.193721	-0.235014
O	5.455287	2.121784	1.729917
O	8.033559	1.866731	1.475126
H	-8.870601	-0.243992	-0.186692
H	-3.364625	-0.727835	0.013354
H	0.348939	-2.564526	0.801708
H	-1.584106	-4.444776	0.976712
H	-8.798348	-2.875505	0.190800
H	-6.800635	-4.659646	0.543744
H	-8.935239	2.465975	-0.600957
H	-7.012811	4.372842	-0.824535
H	-5.168158	0.623518	-0.143928
H	-1.788096	4.521062	-0.824356
H	0.230656	2.787639	-0.484282
H	-4.344660	4.350320	-0.768177
H	-4.200763	-4.467977	0.674518
H	1.055680	-1.351387	-1.388531
H	3.500039	-1.259352	-1.331316
H	3.311083	1.729981	1.766293
H	0.868445	1.635687	1.699872
H	9.131233	-2.819549	-2.388381
H	10.771020	-1.357778	-1.219868
H	6.685246	-2.477831	-2.061748
H	9.953056	0.458542	0.287227
H	6.290843	2.537475	2.052677

11T T_1 minimum, MeOH, B3LYP/6-31g*, E = −1791.605008 a.u.

C	-1.845840	3.464977	-0.590523
C	-3.080430	2.708686	-0.430477
N	-2.804459	1.381014	-0.148900
C	-1.442885	1.288905	-0.123237
C	-0.828813	2.589587	-0.413172
C	-4.331186	3.282860	-0.565035
C	-5.623212	2.682953	-0.494953
N	-5.865096	1.347142	-0.290449
C	-7.215582	1.100268	-0.312020
C	-7.864104	2.332611	-0.532283
C	-6.872794	3.314946	-0.647066
C	-7.786644	-0.194847	-0.149582
C	-7.093052	-1.389181	0.045130
C	-7.730464	-2.695862	0.209612
C	-6.728817	-3.590268	0.386865
C	-5.479774	-2.831640	0.329949
N	-5.730295	-1.494416	0.116869
C	-4.227176	-3.397560	0.489397
C	-2.940928	-2.776049	0.469050
N	-2.678398	-1.451090	0.204373
C	-1.328098	-1.204651	0.293768
C	-0.708200	-2.424929	0.634618
C	-1.709136	-3.398539	0.732072
C	-0.717993	0.092024	0.101144
C	0.762642	0.142348	0.144489
C	1.544942	-0.679798	-0.690480
C	2.931635	-0.626213	-0.661100
C	3.606689	0.245713	0.218896
C	2.828718	1.061347	1.066346
C	1.440570	1.008693	1.022878
C	5.071531	0.279829	0.233711
O	5.625470	-0.686046	-0.568637
C	6.970607	-0.826918	-0.711121
C	7.879233	0.009109	-0.041655
C	7.352605	1.051182	0.822938
C	5.898698	1.132366	0.918230
C	7.413866	-1.850336	-1.558949
C	8.778242	-2.029740	-1.731875
C	9.707348	-1.201824	-1.070555
C	9.260732	-0.193617	-0.234744
O	5.455092	2.122434	1.729080
O	8.033396	1.867485	1.474470
H	-8.870656	-0.243882	-0.186325
H	-3.364616	-0.727886	0.013165
H	0.348906	-2.564709	0.801384
H	-1.584201	-4.444884	0.976481
H	-8.798424	-2.875410	0.191034
H	-6.800719	-4.659614	0.543739
H	-8.935252	2.466118	-0.600477
H	-7.012772	4.372931	-0.824161
H	-5.168185	0.623511	-0.143940
H	-1.788066	4.520954	-0.824601

H	0.230678	2.787510	-0.484508
H	-4.344615	4.350302	-0.768120
H	-4.200849	-4.468018	0.674299
H	1.055758	-1.351530	-1.388653
H	3.500108	-1.259458	-1.331396
H	3.311057	1.730011	1.766077
H	0.868423	1.635663	1.699635
H	9.131549	-2.820332	-2.387046
H	10.771189	-1.357978	-1.219056
H	6.685527	-2.478642	-2.060664
H	9.953041	0.458909	0.287255
H	6.290598	2.538309	2.051732

flavonol 1 S_0 minimum, MeOH, B3LYP/6-31+g*, E = −803.331856 a.u.

C	0.044316	-0.029017	-0.058537
C	-0.114379	0.027947	1.343522
C	0.995960	0.045847	2.185725
C	2.289145	0.007510	1.653300
C	2.458840	-0.048994	0.267045
C	1.353538	-0.067329	-0.584911
H	-1.108984	0.058187	1.772407
H	0.849332	0.089909	3.261590
H	3.153575	0.021543	2.311622
H	3.457938	-0.079212	-0.159369
H	1.506992	-0.111237	-1.654379
C	-1.144434	-0.046764	-0.923455
C	-1.210828	-0.098872	-2.293404
C	-2.476871	-0.110001	-3.011858
C	-3.679172	-0.062435	-2.196454
C	-3.534038	-0.010348	-0.799998
O	-2.310202	-0.003614	-0.206118
O	-0.109776	-0.144168	-3.089001
O	-2.452666	-0.158237	-4.262093
C	-4.648307	0.037336	0.049596
C	-5.917012	0.032470	-0.512996
H	-4.499512	0.076916	1.123976
H	-6.787518	0.069146	0.135630
C	-6.087340	-0.019377	-1.912704
C	-4.980227	-0.066249	-2.743757
H	-7.087545	-0.022400	-2.335324
H	-5.088275	-0.106547	-3.823046
H	-0.472194	-0.174852	-4.004341

flavonol 1 T_1 minimum, MeOH, B3LYP/6-31+g*, E = −803.251759 a.u.

C	0.041513	-0.023977	-0.053128
C	-0.098782	0.037699	1.374768
C	1.013537	0.059086	2.200326
C	2.312021	0.020936	1.662146
C	2.472184	-0.039625	0.266627
C	1.375142	-0.062100	-0.580545
H	-1.091400	0.067773	1.807968

H	0.875150	0.106004	3.277517
H	3.179502	0.038009	2.315432
H	3.470600	-0.069588	-0.162054
H	1.532538	-0.108900	-1.649322
C	-1.115803	-0.044257	-0.865452
C	-1.231414	-0.104129	-2.323437
C	-2.481728	-0.118817	-3.023670
C	-3.691929	-0.070336	-2.208761
C	-3.550800	-0.013135	-0.810279
O	-2.312739	-0.001559	-0.190989
O	-0.147686	-0.148656	-3.080018
O	-2.442693	-0.172678	-4.295294
C	-4.656163	0.035238	0.030841
C	-5.940842	0.026665	-0.530985
H	-4.509329	0.078750	1.105479
H	-6.806982	0.064370	0.123318
C	-6.106977	-0.029754	-1.917676
C	-4.985969	-0.077987	-2.751123
H	-7.103595	-0.036214	-2.349001
H	-5.095753	-0.122122	-3.830296
H	-0.501244	-0.183537	-4.009727

flavonol-tautomer 1T T_1 minimum, MeOH, B3LYP/6-31+g*, E = −803.268757 a.u.

C	-4.917946	-0.079478	-2.766833
C	-3.620706	-0.070889	-2.205037
C	-3.501802	-0.013032	-0.795220
C	-4.616576	0.034327	0.024312
C	-5.897612	0.024802	-0.555366
C	-6.044260	-0.031912	-1.945311
O	-2.269042	-0.000673	-0.167952
C	-1.070974	-0.044057	-0.847823
C	-1.117438	-0.104804	-2.306032
C	-2.415482	-0.116282	-2.952618
C	0.083752	-0.022251	0.000460
C	-0.081510	0.039553	1.415219
C	1.020261	0.061949	2.262440
C	2.321960	0.024143	1.743073
C	2.503608	-0.036816	0.353209
C	1.414002	-0.060009	-0.509460
O	-0.095519	-0.147076	-3.040264
O	-2.439243	-0.172210	-4.297628
H	-1.080393	0.069417	1.833980
H	0.864985	0.109274	3.337240
H	3.180111	0.041846	2.409149
H	3.508029	-0.066583	-0.061312
H	1.571100	-0.107113	-1.578564
H	-4.485942	0.078105	1.101027
H	-6.772209	0.061954	0.087165
H	-7.035412	-0.039073	-2.389051
H	-5.021346	-0.123651	-3.846113
H	-1.500953	-0.195660	-4.588218

porphin S_0 minimum, MeOH, B3LYP/6-31+g*, E = −989.595788 a.u.

C	-3.816149	0.772444	0.816042
C	-3.987507	0.316897	-0.560247
N	-5.174503	-0.344529	-0.712380
C	-5.766010	-0.325580	0.520237
C	-4.927915	0.371693	1.491031
C	-3.044020	0.540500	-1.573642
C	-3.129994	0.140194	-2.908180
N	-4.175036	-0.555666	-3.469047
C	-3.945333	-0.791106	-4.804239
C	-2.669700	-0.205155	-5.109367
C	-2.174663	0.360123	-3.958248
C	-4.805113	-1.470946	-5.668664
C	-6.048799	-2.037417	-5.353224
N	-6.640262	-2.018553	-4.120585
C	-7.827296	-2.679907	-4.272737
C	-7.998430	-3.135883	-5.648912
C	-6.887076	-2.734344	-6.324110
C	-8.770781	-2.903512	-3.259342
C	-8.684823	-2.503175	-1.924812
C	-9.640126	-2.723160	-0.874729
C	-9.145175	-2.157716	0.276347
C	-7.869500	-1.571847	-0.028761
N	-7.639797	-1.807286	-1.363953
C	-7.009731	-0.891986	0.835659
H	-7.348998	-0.795643	1.863213
H	-5.004240	-0.855190	-2.965418
H	-2.207714	-0.224104	-6.088030
H	-1.236647	0.884776	-3.829986
H	-5.172196	0.522558	2.536039
H	-2.960321	1.320058	1.193059
H	-9.607198	-2.138697	1.254990
H	-10.578111	-3.247872	-1.002975
H	-6.810600	-1.507750	-1.867584
H	-8.854097	-3.683803	-6.025847
H	-6.642949	-2.884914	-7.369198
H	-4.465915	-1.567154	-6.696254
H	-9.670254	-3.447176	-3.534360
H	-2.144489	1.084053	-1.298594

porphin T_1 minimum, MeOH, B3LYP/6-31+g*, E = −989.538215 a.u.

C	-3.808257	0.783523	0.839483
C	-3.974707	0.328400	-0.542010
N	-5.168088	-0.336562	-0.695567
C	-5.761464	-0.316780	0.544184
C	-4.916045	0.383252	1.513247
C	-3.042174	0.547473	-1.550800
C	-3.115041	0.144439	-2.921234
N	-4.162535	-0.551368	-3.476919
C	-3.927528	-0.783789	-4.811388
C	-2.677383	-0.212409	-5.120769

C	-2.171495	0.365581	-3.944046
C	-4.818985	-1.482535	-5.684674
C	-6.053358	-2.046194	-5.377176
N	-6.646743	-2.026394	-4.137430
C	-7.840112	-2.691381	-4.290982
C	-8.006646	-3.146341	-5.672518
C	-6.898716	-2.746340	-6.346210
C	-8.772644	-2.910455	-3.282192
C	-8.699770	-2.507434	-1.911754
C	-9.643322	-2.728563	-0.888945
C	-9.137396	-2.150644	0.287796
C	-7.887278	-1.579214	-0.021599
N	-7.652270	-1.811638	-1.356067
C	-6.995825	-0.880461	0.851687
H	-7.337419	-0.785618	1.878916
H	-4.991730	-0.851156	-2.974133
H	-2.212288	-0.229367	-6.097810
H	-1.232820	0.889656	-3.819629
H	-5.158928	0.534436	2.558468
H	-2.952784	1.331488	1.216653
H	-9.602472	-2.133720	1.264847
H	-10.582007	-3.252619	-1.013365
H	-6.823069	-1.511863	-1.858851
H	-8.862177	-3.694194	-6.049718
H	-6.655782	-2.897620	-7.391405
H	-4.477373	-1.577416	-6.711894
H	-9.672929	-3.454113	-3.555677
H	-2.141909	1.091168	-1.277324

Synthesis of Hybrids 2

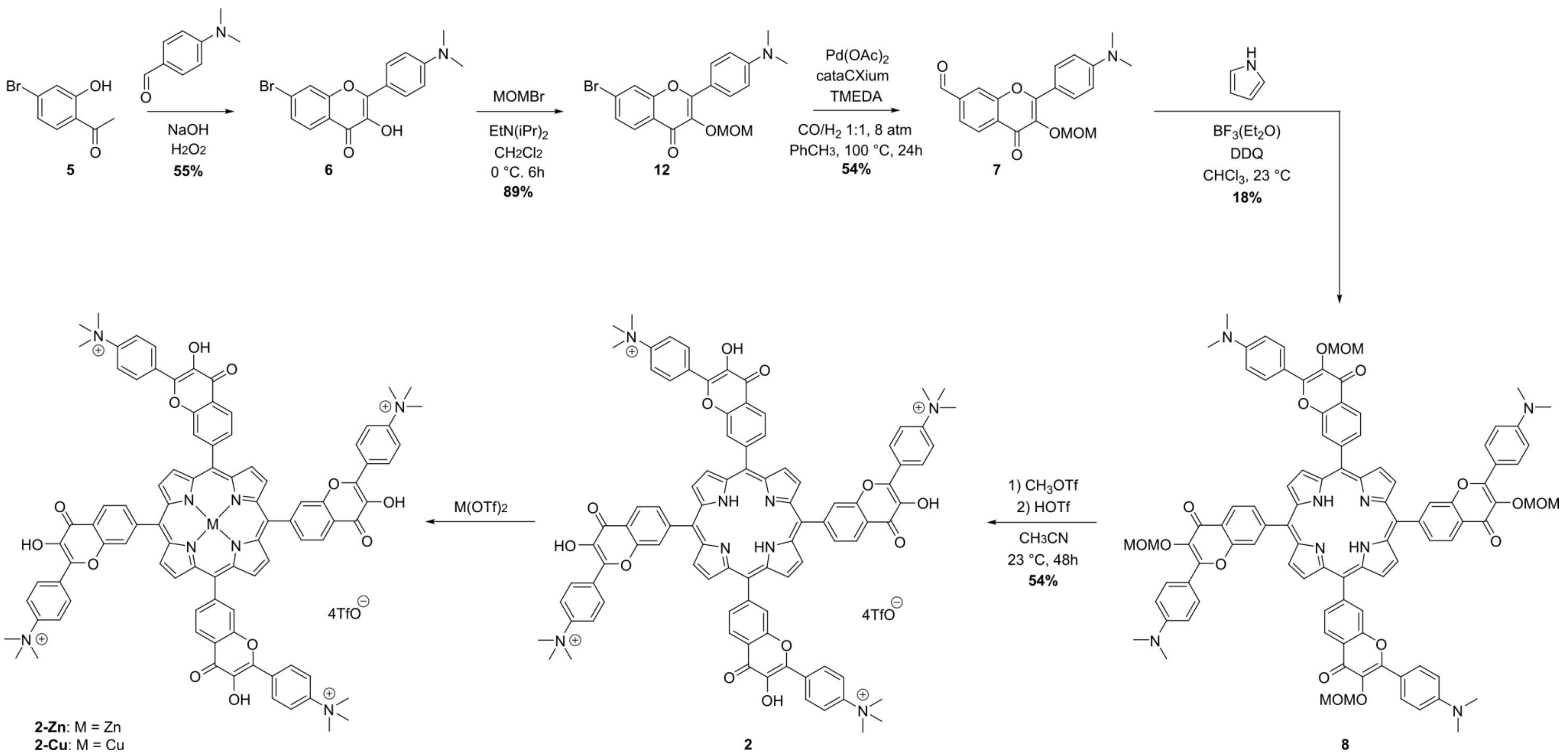

Scheme S3. Synthesis of porphyrin-flavonol hybrid **2** and its metal complexes **2-Zn**, and **2-Cu**.

7-Bromo-2-(4-(dimethylamino)phenyl)-3-hydroxy-4*H*-chromen-4-one (6). 1-(4-Bromo-2-hydroxyphenyl)ethan-1-one (17.8 g, 82.8 mmol), 4-(dimethylamino)benzaldehyde (14.8 g, 99.3 mmol), and NaOH (16.6 g, 413.9 mmol) were dissolved in methanol (500 mL) and refluxed for 5 h. The solvent

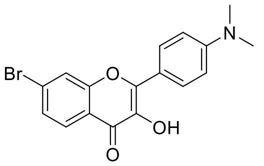

was removed under reduced pressure, and the solid residue was redissolved in methanol (300 mL). The cycle was repeated 3 times to push the equilibrium towards the product of the condensation. The solid was then dissolved in methanol (400 mL), kept under stirring at 0 °C, and H_2O_2 (30%; 60 mL, 580 mmol) was added dropwise, while an orange precipitate was formed. The reaction mixture was stirred for 16 h, and aq. HCl was added until the pH was adjusted to 6. The precipitate changed color to yellow, was collected through vacuum filtration, washed with water (3 ×100 mL), methanol (3 × 50 mL), and vacuum dried to afford a bright yellow solid. Yield: 16.4 g (55%). ^{1}H NMR (300 MHz, d_6-DMSO): δ (ppm) 9.28 (s, 1H), 8.19–7.95 (m, 4H), 7.65−7.55 (m, 1H), 6.90−6.79 (m, 2H), 3.03 (s, 6H). ^{13}C NMR (125 MHz, d_6-DMSO): δ (ppm) 171.9, 154.9, 151.6, 147, 6, 137.9, 129.5, 128.0, 127.0, 126.5, 121.5, 121.1, 118.1, 111.9, 40.1. HRMS (APCI+): calcd. for $C_{17}H_{15}BrNO_3^+$ [M + H^+] 360.0230, found 360.0233. The spectroscopic data are consistent with those reported in the literature.[12]

7-Bromo-2-(4-(dimethylamino)phenyl)-3-(methoxymethoxy)-4*H*-chromen-4-one (12). Flavonol **6** (10.5 g, 29.2 mmol) was partially dissolved in a CH_2Cl_2/THF mixture (3:2, v/v; 150 mL), and the solution was kept at 0 °C. *N*,*N*-diisopropylethylamine (DIPEA, 5.2 mL, 87.5 mmol) and bromomethyl methyl ether (MOMBr, 7.14 mL, 87.5 mmol) were added dropwise. The solution

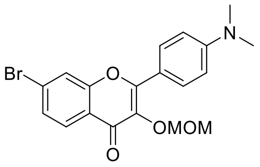

became homogeneous within 2 h and was left under stirring for 16 h. The reaction mixture was washed with sat. aq. $NaHCO_3$ (2 × 50 mL), and water (2 × 50 mL), sat. aq. NaCl (50 mL), and dried over $MgSO_4$. The solvent was evaporated under reduced pressure to afford a pale orange solid. Yield: 10.5 g (89%). ^{1}H NMR (500 MHz, d_6-DMSO): δ (ppm) 8.10−8.03 (m, 2H), 7.99−7.95 (m, 1H), 7.65− 7.61 (m, 1H), 6.87−6.83 (m, 2H), 5.18 (s, 2H), 3.21 (s, 3H), 3.04 (s, 6H). ^{13}C NMR (125 MHz, d_6-DMSO): δ (ppm) 173.0, 157.0, 155.1, 152.3, 136.3, 130.5, 128.6, 127.2, 127.0, 123.0, 121.5, 116.8, 111.6, 97.2, 57.6. HRMS (APCI+): calcd. for $C_{19}H_{19}BrNO_4^+$ [M + H^+] 404.0492, found 404.0495.

2-(4-(Dimethylamino)phenyl)-3-(methoxymethoxy)-4-oxo-4*H*-chromene-7-carbaldehyde (7). Flavonol **12** (10.5 g, 26.0 mmol) was partially dissolved in toluene (50 mL), and the solution was purged with argon for 30 min. Under an Ar atmosphere, tetramethylethylenediamine (TMEDA, 2.92 mL, 19.5 mmol), $Pd(OAc)_2$ (64 mg, 0.29 mmol), and di(1-adamantyl)-n-butylphosphine

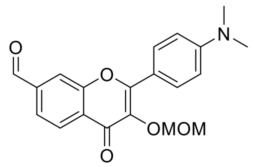

(cataCXium®, 307 mg, 0.86 mmol) were added, and the reaction mixture was transferred to a pressurized autoclave. After 24 h at a constant pressure of 10 bar of CO/H_2 (1:1) at 100 °C, an aliquot was taken from the reaction mixture for a TLC analysis (pentane/ethyl acetate 4:1). After the starting material was consumed, the reaction mixture was diluted with CH_2Cl_2, filtered through celite, and the solvents were evaporated under reduced pressure. The crude solid residue was recrystallized through dropwise addition of pentane into CH_2Cl_2, and the precipitate was washed with pentane (3×20 mL), cold methanol (10 mL), and vacuum dried to afford an orange solid. Yield: 4.96 g (54%). ^{1}H NMR (300 MHz,): δ (ppm) 10.17 (s, 1H), 8.44−8.8.38 (m, 1H), 8.17−8.10 (m, 2H), 8.04 (s, 1H), 7.91−7.85 (m, 1H), 6.93−6.86 (m, 2H), 5.29 (s, 2H), 3.32 (s, 3H), 3.12 (s, 6H). ^{13}C NMR (125 MHz, d_6-DMSO): δ (ppm) 190.9, 173.6, 158.2, 155.0, 151.5, 139.4, 137.3, 130.5, 128.0, 127.0, 124.1, 119.6, 111.8, 97.4, 57.8, 43.3, 40.4. HRMS (APCI+): calcd. for $C_{20}H_{20}NO_5^+$ [M + H^+] 354.1336, found 354.1339.

7,7',7'',7'''-(Porphyrin-5,10,15,20-tetrayl)tetrakis(2-(4-(dimethylamino)phenyl)-3-(methoxymethoxy)-4H-chromen-4-one) (8). Aldehyde **7** (1.80 g, 5.09 mmol) was dissolved in CH_2Cl_2 (610 mL), and the solution was purged with N_2 for 30 min. Freshly distilled pyrrole (353 μL, 5.09 mmol) was added, followed by the addition of trifluoroacetic acid (TFA, 1.95 mL, 25.47 mmol). The solution was stirred at 23 °C for 1 h under N_2 atmosphere in the complete dark. Then, 2,3-dichloro-5,6-dicyano-1,4-benzoquinone (DDQ, 867 mg, 3.82 mmol) was added, and the mixture was stirred for 1 h at 45 °C. The solution was left to cool down to 23 °C, then triethylamine (TEA, 3.55 mL, 25.47 mmol) and an amberlyst A-21 ion-exchange resin (1.0 g) were added, and after 30 min of stirring, the reaction mixture was filtered through a filter paper. The solvent was evaporated at reduced pressure, and methanol (10 mL) was added. The resulting precipitate was vacuum filtered, washed with methanol (3×10 mL), redissolved in CH_2Cl_2, and purified by column chromatography (silica gel, CH_2Cl_2/methanol, 1 to 4% of methanol) to afford a dark purple solid. Yield: 360 mg (18%). ^{1}H NMR (300 MHz, CD_3Cl): δ (ppm) 8.97 (s, 8H), 8.67−8.64 (d, 4H), 8.44 (s, 4H), 8.32−8.30 (d, 4H), 8.21−8.18 (d, 8H), 6.81−6.78 (d, 8H), 5.44 (s, 8H), 3.43 (s, 12H), 3.08 (s, 24H), –2.70 (s, 2H). ^{13}C NMR (125 MHz, CD_3Cl): δ (ppm) 174.5, 158.1, 153.8, 151.8, 146.8, 137.0, 131.4, 131.0, 130.7, 130.5, 123.8, 123.7, 118.9, 117.8, 111.2, 97.6, 57.9, 43.2, 40.0. HRMS (MALDI+): calcd. for $C_{96}H_{83}N_8O_{16}^+$ [M + H$^+$] 1603.588, found 1603.547.

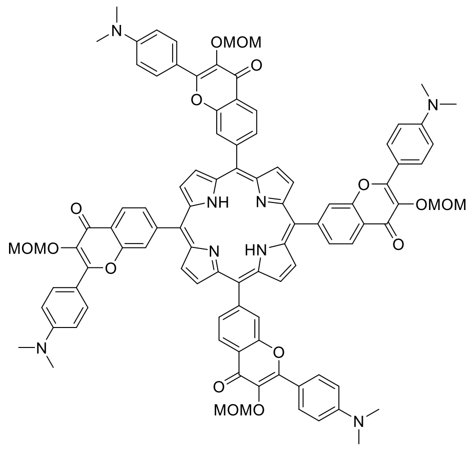

4,4',4'',4'''-(Porphyrin-5,10,15,20-tetrayltetrakis(3-hydroxy-4-oxo-4H-chromene-7,2-diyl))tetrakis(N,N,N-trimethylbenzenaminium trifluoromethanesulfonate) (2). **8** (197 mg, 0.1228 mmol) was dissolved in dry CH_3CN (8 mL), and the solution was purged with N_2 for 10 min. Under N_2 atmosphere, CH_3OTf (80 μL, 0.737 mmol) was added dropwise, and the mixture was stirred at 23 °C for 24 h. HOTf (80 μL, 0.737 mmol) was added dropwise, and the mixture was stirred at 23 °C for 24 h. The reaction was monitored by reverse phase HPLC, and after completion, a cross-linked poly(4-vinylpyridine) ion-exchange resin (600 mg) was added, and the mixture was stirred for 1 h. Then, the resin was removed by filtration, the solvent was evaporated under reduced pressure, and the solid residue dissolved in DMF (1.6 mL). CH_2Cl_2 was slowly added (10 mL, 12 μL min^{-1}) to the stirred solution, and the resulting precipitate was vacuum filtered, washed with CH_2Cl_2 (5×5 mL), and vacuum dried to afford a dark purple solid. Yield: 137 mg (54%). ^{1}H NMR (500 MHz, d_6-DMSO): δ (ppm) 10.30 (s, 4H), 9.03 (s, 8H), 8.74 (s, 4H), 8.54 (m, 12H), 8.35 (s, 4H), 8.17-8.16 (d, 8H), 3.66 (s, 36H), –2.82 (s, 2H). ^{13}C NMR (125 MHz, d_6-DMSO): δ (ppm) 173.9, 154.0, 148.0, 147.0, 144.3, 141.1, 133.5, 129.6, 125.0, 124.5, 123.8, 122.5, 121.6, 121.4, 119.9, 119.1, 117.3, 56.9. ^{19}F NMR (476 MHz, d_6-DMSO): –77.7 (s). HRMS (MALDI+): calcd. for $C_{95}H_{78}F_9N_8O_{21}S_3^+$ [M – TfO$^-$] 1933.429, found 1933.426.

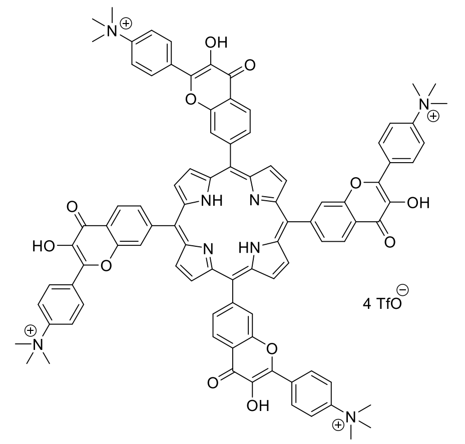

(2-Zn). **2** (20 mg, 0.0096 mmol) and $Zn(OTf)_2$ (17.5 mg, 0.048 mmol) were dissolved in DMF (3 mL), and the solution was stirred at 130 °C for 2 h. The progress of the reaction was monitored by UV-vis spectroscopy and RP-HPLC. The solvent was partially evaporated under reduced pressure to obtain a volume of approximately 500 µL, and CH_2Cl_2 was slowly added (1.5 mL, 12 µL min^{-1}) to the stirred solution. The resulting precipitate was vacuum filtered, washed with CH_2Cl_2 (5 × 1 mL), and vacuum dried until no traces of solvents were present to afford a dark purple solid. Yield: 15.9 mg (77%). ^{1}H NMR (500 MHz, d_6-DMSO): δ (ppm) 10.27 (s, 4H), 8.95 (s, 8H), 8.67 (m, 4H), 8.54 (m, 12H), 8.33 (d, 4H), 8.16 (m, 8H), 3.66 (s, 36H). ^{13}C NMR (125 MHz, d_6-DMSO): δ (ppm) 174.0, 153.8, 149.6, 148.7, 148.0, 144.3, 141.0, 133.5, 132.7, 129.6, 125.0, 123.3, 122.4, 121.3, 119.9, 119.4, 117.3, 56.9. ^{19}F NMR (476 MHz, d_6-DMSO): –77.7 (s). HRMS (MALDI+): calcd. for $C_{95}H_{76}F_9N_8O_{21}S_3Zn^+$ [M – TfO$^-$] 1995.343, found 1995.348.

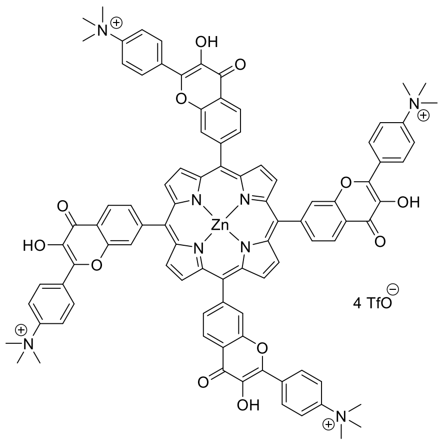

(2-Cu). **2** (12.0 mg, 0.0096 mmol) and $Cu(OTf)_2$ (15 mg, 0.042 mmol) were dissolved in CH_3OH (1.8 mL), and the solution was stirred at 23 °C for 2 h. The progress of the reaction was monitored by UV-vis spectroscopy and RP-HPLC. The solvent was partially evaporated under reduced pressure to obtain a volume of approximately 300 µL, and CH_2Cl_2 was slowly added (2 mL, 4 µL min^{-1}) to the stirred solution. The resulting precipitate was vacuum filtered, washed with CH_2Cl_2 (5×1 mL), and vacuum dried to afford a dark purple solid. Yield: 9.1 mg (73%). The NMR spectra are not available due to the paramagnetic nature of Cu(II) complexes. HRMS (MALDI+): calcd. for $C_{95}H_{76}CuF_9N_8O_{21}S_3^+$ [M – TfO$^-$] 1994.343, found 1994.32.

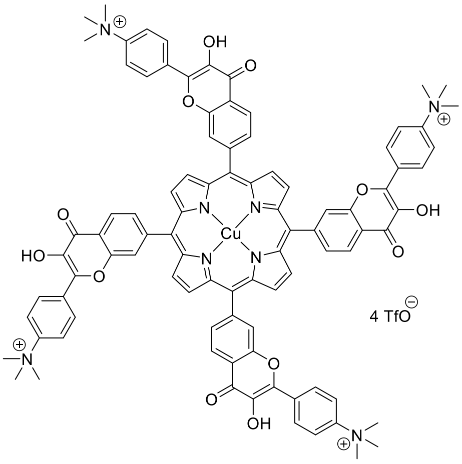

Synthesis of Hybrid 3

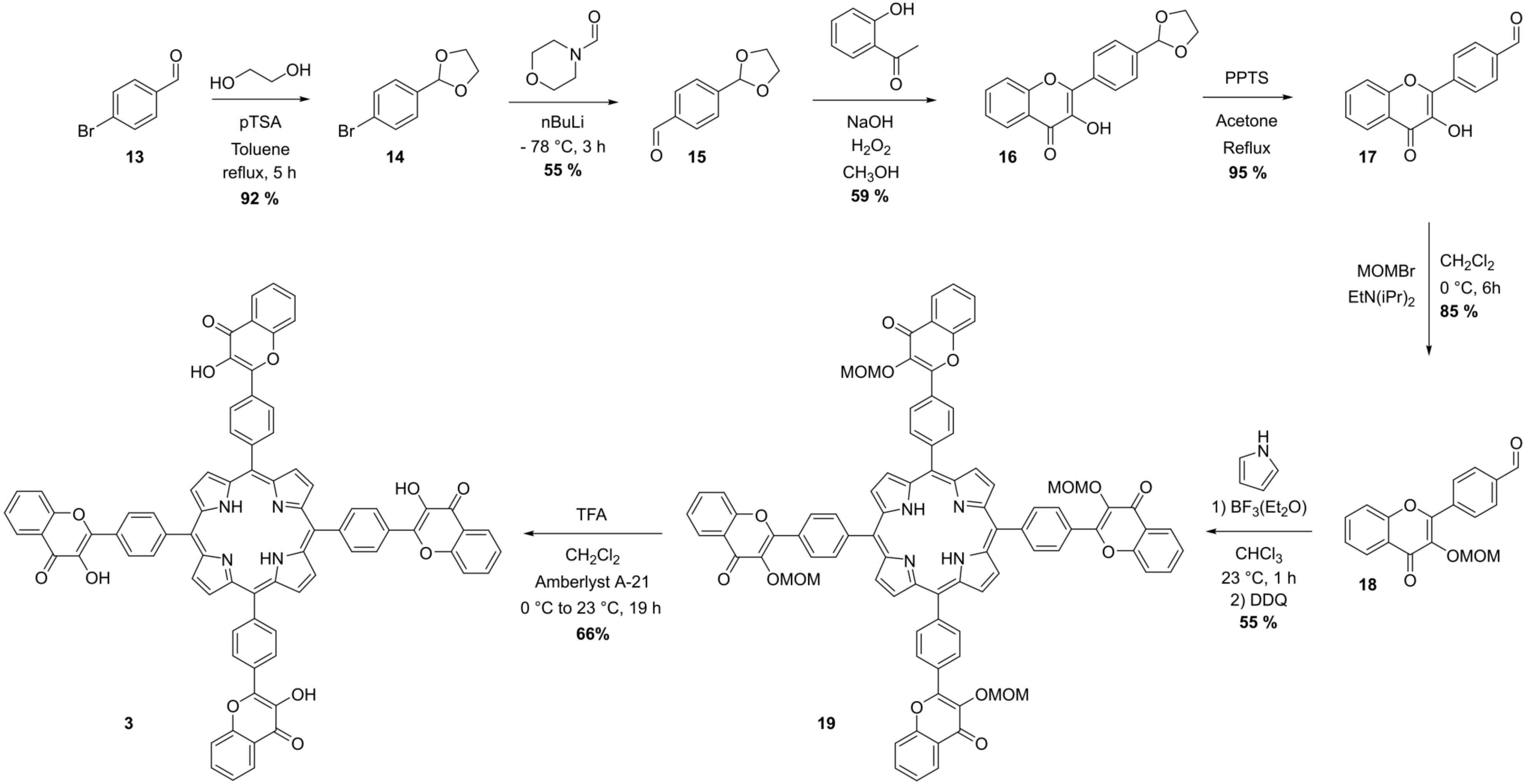

Scheme S4. Synthesis of the porphyrin-flavonol hybrid **3**.

2-(4-Bromophenyl)-1,3-dioxolane (14). 4-Bromobenzaldehyde (50 g, 270.2 mmol), ethylene glycol (76 g, 1.22 mol), and *p*-toluenesulfonic acid (2.5 g, 14.5 mmol) were dissolved in toluene (200 mL) and refluxed with a Dean-Stark trap for 18 h, until no more starting material was detected on TLC (hexane/ethyl acetate 19 : 1). The reaction mixture was washed with sat. aq. $NaHCO_3$ (2×100 mL) and sat. aq. NaCl (2×100 mL). The organic phase was dried over $MgSO_4$, and the solvent was evaporated at reduced pressure. The solid residue was redissolved in methanol (700 mL) and washed with sat. aq. $NaHSO_3$ (150 mL). Water (1000 mL) was added, and the mixture was extracted with hexane/ethyl acetate (1 : 1; 3 × 200 mL). The combined organic phase was dried over $MgSO_4$, and the solvents were evaporated at reduced pressure to afford a yellow oil. Yield: 56.7 g (92%). ^{1}H NMR (300 MHz, $CDCl_3$): δ (ppm) 7.57−7.35 (m, 4H), 5.80 (s, 1H), 4.20−4.00 (m, 4H). The spectroscopic data are consistent with those reported in the literature.[13]

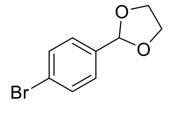

4-(1,3-Dioxolan-2-yl)benzaldehyde (15). *n*-Butyl lithium (200 mL, 2.5 M in hexane, 500.0 mmol) was added dropwise to a stirred solution of **14** (68.2 g, 297.7 mmol) in dry THF (800 mL) at –78 °C under N_2 atmosphere. The reaction mixture was stirred at –78 °C for 1 h, and 4-formylmorpholine (33 mL, 327.4 mmol) was added dropwise. After 100 min, no more starting material was detected on TLC (hexane/ethyl acetate, 2:1). The solution was warmed up to 23 °C, diluted with water (500 mL), and extracted with diethyl ether (500 mL). The organic layer was separated, washed with sat. aq. NaCl (2×200 mL), dried over $MgSO_4$, and filtered. The solvent was evaporated at reduced pressure. The crude product was purified by vacuum distillation to afford a colorless oil. Yield: 29.1 g (55%). ^{1}H NMR (300 MHz, $CDCl_3$): δ (ppm) 10.06 (s, 1H), 7.95−7.65 (m, 4H), 5.90 (s, 1H), 4.20−4.05 (m, 4H). The spectroscopic data are consistent with those reported in the literature.[14]

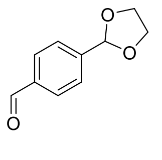

2-(4-(1,3-Dioxolan-2-yl)phenyl)-3-hydroxy-4*H*-chromen-4-one (16). 4-(1,3-dioxolan-2-yl)benzaldehyde **15** (7.17 g, 40.2 mmol), 2-hydroxyacetophenone (4.4 mL, 36.6 mmol), and NaOH (7.32 g, 182.3 mmol) were dissolved in methanol (100 mL), and the resulting mixture was stirred at 23 °C for 3 h. The solvent was evaporated at reduced pressure, and the solid residue was redissolved in methanol (150 mL). This process was repeated 3 times to shift the equilibrium of the aldol condensation towards the products. Hydrogen peroxide (30%, 14.2 mL, 164.6 mmol) was added dropwise at 0 °C, and the reaction mixture was stirred at 23 °C for 16 h. Afterward, an ice-water mixture was added, followed by aq. HCl (1 M) to adjust the pH to ~6, and the mixture was stirred for 2 h. The precipitate was filtered, washed with water (3×100 mL), and vacuum dried to afford a pale-yellow solid. Yield: 4.82 g (59%). ^{1}H NMR (300 MHz, d_6-DMSO): δ (ppm) 9.72 (brs, 1H), 8.29−8.24 (m, 2H), 8.16−8.11 (m, 1H), 7.86−7.76 (m, 2H), 7.66−7.61 (m, 2H), 7.52−7.46 (m, 1H), 5.83 (s, 1H), 4.15−3.95 (m, 4H). ^{13}C NMR (125 MHz, d_6-DMSO): δ (ppm) 173.5, 155.1, 145.2, 140.1, 139.8, 134.3, 132.4, 128.0, 127.1, 125.3, 125.1, 121.8, 118.9, 102.8, 65.4. HRMS (APCI+): calcd. for $C_{18}H_{15}O_5^+$ [M + H$^+$] 311.0914, found 311.0913.

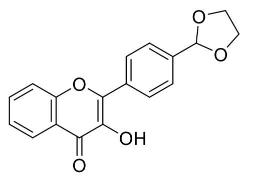

4-(3-Hydroxy-4-oxo-4*H*-chromen-2-yl)benzaldehyde (17). Flavonol **16** (8.0 g, 25.8 mmol) and pyridinium *p*-toluenesulfonate (3.0 g, 11.9 mmol) were dissolved in wet acetone (480 mL) and refluxed for 3 h until no starting material was observed on TLC (CH_2Cl_2). The solvent was removed under reduced pressure, and the residue was redissolved in CH_2Cl_2 (300 mL), washed with sat. aq. $NaHCO_3$ (2×100 mL), sat. aq. NaCl (2×100 mL) and dried over $MgSO_4$. The solvent was evaporated at reduced pressure to afford a pale yellow solid. Yield: 4.83 g (70%). ^{1}H NMR (300 MHz, d_6-DMSO): δ (ppm) 10.10 (s, 1H), 10.05

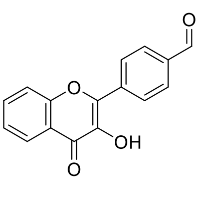

(brs, 1H), 8.48−8.44 (m, 2H), 8.16−8.12 (m, 1H), 8.11−8.07 (m, 2H), 7.87−7.78 (m, 2H), 7.50 (m, 1H). ^{13}C NMR (125 MHz, d_6-DMSO): δ (ppm) 193.2, 173.4, 155.2, 144.0, 140.9, 137.3, 136.7, 134.6, 130.0, 128.5, 125.4, 125.2, 121.8, 119.0. HRMS (APCI+): calcd. for $C_{16}H_{11}O_4^+$ [M + H$^+$] 267.0652, found 267.0650.

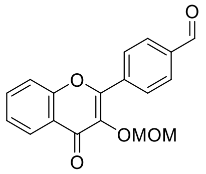

4-(3-(Methoxymethoxy)-4-oxo-4*H*-chromen-2-yl)benzaldehyde (18). 17 (4.83 g, 18.1 mmol) was dissolved in CH_2Cl_2 (100 mL) and cooled to 0 °C. DIPEA (9.48 mL, 54.4 mmol) and then MOMBr (4.44 mL, 54.4 mmol) were added dropwise, the solution was stirred at 0 °C for 30 min, and then the stirred mixture was allowed to warm up to 23 °C under stirring in the complete dark for additional 16 h. The reaction mixture was washed with sat. aq. $NaHCO_3$ (2×50 mL), water (2×50 mL), and sat. aq. NaCl (50 mL), and dried over $MgSO_4$. The solvent was evaporated under reduced pressure to afford a pale-yellow solid. Yield: 4.78 g (85%). ^{1}H NMR (300 MHz, d_6-DMSO): δ (ppm) 10.12 (s, 1H), 8.31−8.26 (m, 2H), 8.14−8.10 (m, 3H), 7.89−7.83 (m, 1H), 7.80−7.75 (m, 1H), 7.55−7.50 (m, 1H), 5.21 (s, 2H), 3.06 (s, 3H). ^{13}C NMR (125 MHz, d_6-DMSO): δ (ppm) 193.3, 174.3, 155.3, 154.9, 138.5, 137.6, 136.2, 134.9, 130.0, 129.8, 125.8, 125.5, 123.9, 119.0, 97.5, 57.4. HRMS (APCI+): calcd. for $C_{18}H_{15}O_5^+$ [M + H$^+$] 311.0914, found 311.0912.

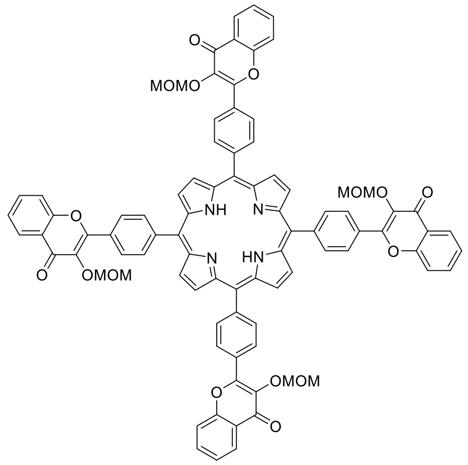

2,2',2'',2'''-(Porphyrin-5,10,15,20-tetrayltetrakis(benzene-4,1-diyl))tetrakis(3-(methoxymethoxy)-4H-chromen-4-one) (19). Aldehyde **18** (2.1 g, 6.8 mmol) was dissolved in dry $CHCl_3$ mixed with 0.75% v/v of ethanol (650 mL). N_2 was purged for 30 min, and the solution was kept under N_2 atmosphere in the complete dark. Freshly distilled pyrrole (470 µL, 6.8 mmol) was added, followed by the addition of $BF_3{\cdot}Et_2O$ (305 µL, 2.1 mmol). The solution was stirred at 23 °C for 1 h, then DDQ (1.15 g, 5.1 mmol) was added, and the mixture was stirred for an additional 1 h. TEA (450 µL, 2.1 mmol) was added, and after 30 min of stirring, the reaction mixture was filtered through a filter paper, the solvents were evaporated under reduced pressure, and the crude solid dissolved in $CHCl_3$ (150 mL) and filtered again. The solid was then dissolved in the least amount of CH_2Cl_2 (36 mL), and methanol (60 mL) was slowly added (100 µL min^{-1}) to a gently stirred solution. The resulting precipitate was vacuum filtered, washed with methanol (5×5 mL), and vacuum dried to afford a purple solid. Yield: 1.27 g (52%). ^{1}H NMR (300 MHz, CD_2Cl_2): δ (ppm) 8.99 (s, 8H), 8.62−8.45 (m, 16H), 8.37−8.32 (m, 4H), 7.88−7.73 (m, 12H), 7.58−7.50 (m, 4H), 5.46 (s, 8H), 3.51 (s, 12H), −2.70 (s, 2H). ^{13}C NMR (125 MHz, CD_2Cl_2): δ (ppm) 174.6, 156.3, 155.6, 144.2, 138.4, 134.6, 133.7, 130.9, 127.5, 125.7, 124.9, 124.3, 119.7, 118.2, 97.8, 57.6. HRMS (MALDI–): calcd. for $C_{88}H_{61}N_4O_{16}^-$ [M – H$^+$] 1429.408, found 1429.411.

2,2',2'',2'''-(Porphyrin-5,10,15,20-tetrayltetrakis(benzene-4,1-diyl))tetrakis(3-hydroxy-4H-chromen-4-one) (3). Porphyrin **19** (700 mg, 0.56 mmol) was dissolved in dry CH_2Cl_2 (15 mL), purged with N_2 for 30 min, and TFA (600 μL, 7.8 mmol) was added dropwise at 0 °C. After 24 h at 23 °C, stabilizer-free THF (25 mL) and an Amberlyst A-21 ion-exchange resin (4.3 g) were added, and the mixture was stirred for 50 min. Then, CH_2Cl_2 (5 mL) was added, and the resulting mixture was filtered through a nylon mesh to remove the resin and through filter paper to collect the dark-purple precipitate as the pure target compound. Yield: 460 mg (66%). ^{1}H NMR (300 MHz, d_7-DMF): δ (ppm) 10.15 (s, 4H), 9.13 (s, 8H), 8.92−8.57 (dd, 16H), 8.31−8.23 (m, 4H), 8.02−7.88 (m, 8H), 7.64−7.54 (m, 4H), −2.62 (s, 2H). ^{13}C NMR (125 MHz, d_7-DMF): δ (ppm) 173.4, 155.4, 145.0, 143.2, 140.3, 134.9, 134.0, 131.6, 126.4, 125.0, 124.7, 121.8, 120.0, 118.7. HRMS (MALDI–): calcd. for $C_{80}H_{45}N_4O_{12}^-$ [M – H$^+$] 1253.303, found 1253.331.

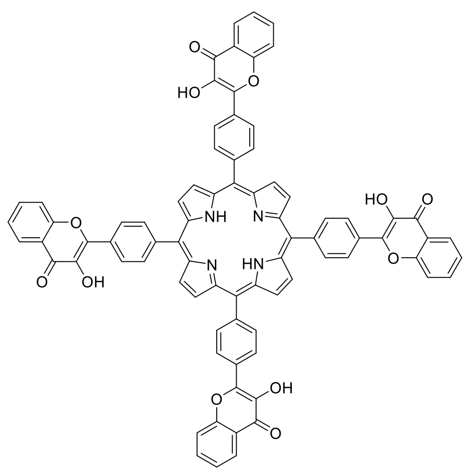

Synthesis of 10

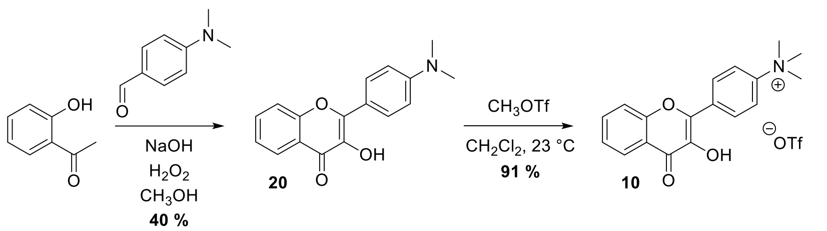

Scheme S5. Synthesis of the water-soluble flavonol **10**.

2-(4-(Dimethylamino)phenyl)-3-hydroxy-4*H*-chromen-4-one (20). 1-(2-hydroxyphenyl)ethan-1-one (2.00 g, 14.7 mmol), 4-(dimethylamino)benzaldehyde (2.19 g, 14.7 mmol), and NaOH (1.76 g, 44.1 mmol) were dissolved in methanol (100 mL) and refluxed for 3 h. The solvent was evaporated under reduced pressure, and the solid residue was redissolved in methanol (100 mL). The cycle was repeated 3 times to push the equilibrium towards the product of the condensation. The solid was then dissolved in methanol (100 mL), kept under stirring at 0 °C, and H_2O_2 (30%; 7 mL, 68 mmol) was added dropwise while an orange precipitate was formed. The reaction mixture was stirred for 3 h, and aq. HCl was added until the pH was adjusted to 6. The precipitate changed color to yellow, then it was collected through vacuum filtration, washed with water (3×10 mL), methanol (3×5 mL), and vacuum dried to afford a bright yellow solid. Yield: 2.07 g (40%). ^{1}H NMR (300 MHz, d_6-DMSO): δ (ppm) 9.12 (bs, 1H), 8.14–8.07 (m, 3H), 7.75−7.73 (m, 2H), 7.46−7.41 (t, 1H), 6.86−6.83 (d, 2H), 3.02 (s, 6H). ^{13}C NMR (125 MHz, d_6-DMSO): δ (ppm) 172.4, 154.8, 151.5, 147.3, 137.7, 133.5, 129.4, 125.1, 124.7, 121.9, 118.6, 118.4, 111.9, 40.1. HRMS (APCI+): calcd. for $C_{17}H_{16}NO_3^+$ [M + H$^+$] 282.1125, found 282.1128. The spectroscopic data are consistent with those reported in the literature.[15]

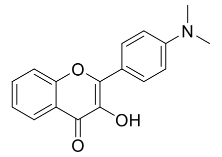

4-(3-Hydroxy-4-oxo-4*H*-chromen-2-yl)-*N*,*N*,*N*-trimethylbenzenaminium trifluoromethanesulfonate (10). Flavonol **20** (512 mg, 1.82 mmol) was dissolved in dry CH_2Cl_2 (10 mL), and the solution was purged with N_2 for 10 min. Under N_2 atmosphere, CH_3OTf (300 μL, 2.73 mmol) was added dropwise, and the mixture was stirred at 23 °C for 18 h. A pale yellow precipitate was formed. This precipitate was vacuum

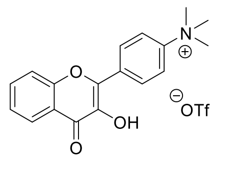

filtered, washed with CH_2Cl_2 (4×8 mL), and vacuum dried to afford an off-white solid. Yield: 737 mg (91%). ^{1}H NMR (500 MHz, d_6-DMSO): δ (ppm) 10.00 (bs, 1H), 8.42−8.39 (m, 2H), 8.15−8.13 (m, 3H), 7.85−7.78 (m, 2H), 7.52−7.48 (t, 1H), 3.67 (s, 9H). ^{13}C NMR (125 MHz, d_6-DMSO): δ (ppm) 173.7, 155.1, 148.0, 143.7, 140.4, 134.5, 133.4, 129.5, 125.4, 125.3, 121.8, 121.3, 119.0, 56.9. ^{19}F NMR (476 MHz, d_6-DMSO): –77.7 (s). HRMS (ESI+): calcd. for $C_{18}H_{18}NO_3^+$ [M$^+$] 296.1281, found 296.1283.

Emission and Absorption Spectra

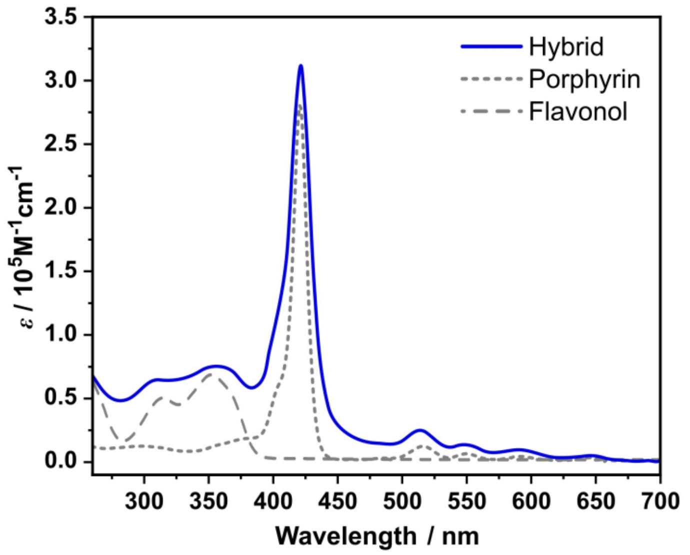

Figure S4A. UV-Vis absorption spectra of a methanol solution of the porphyrin-flavonol hybrid **3** (blue line), a free-base porphyrin (tetra(4-carboxyphenyl)porphyrin; gray dotted line), and flavonol (**1**, 3-hydroxyflavone; gray dashed line).

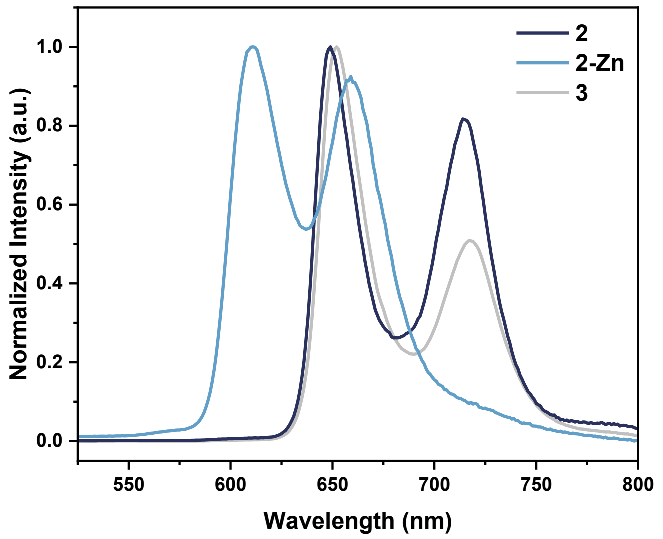

Figure S4B. Normalized emission spectra of **2**, **2-Zn** (methanol), and **3** (methanol/DMSO, 4:1, v/v) solution (T = 23 °C).

Stabilities of Compounds 2 and 3 in the Dark

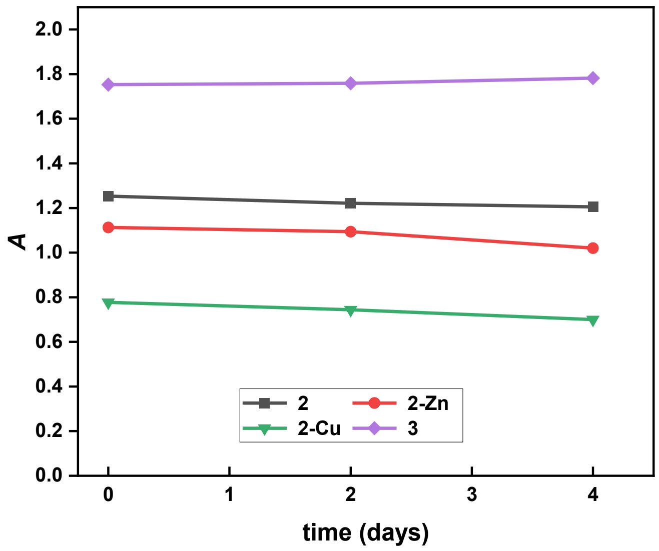

Figure S5. Dark stability of hybrids **2**, **2-Zn**, **2-Cu** (methanol), and **3** (DMSO) at room temperature (c = 5 μM) monitored by UV-vis spectroscopy. The data are expressed as the absorbance of the solution at the maximum of absorption over time.

Photoirradiation Experiments in Aerated Solutions

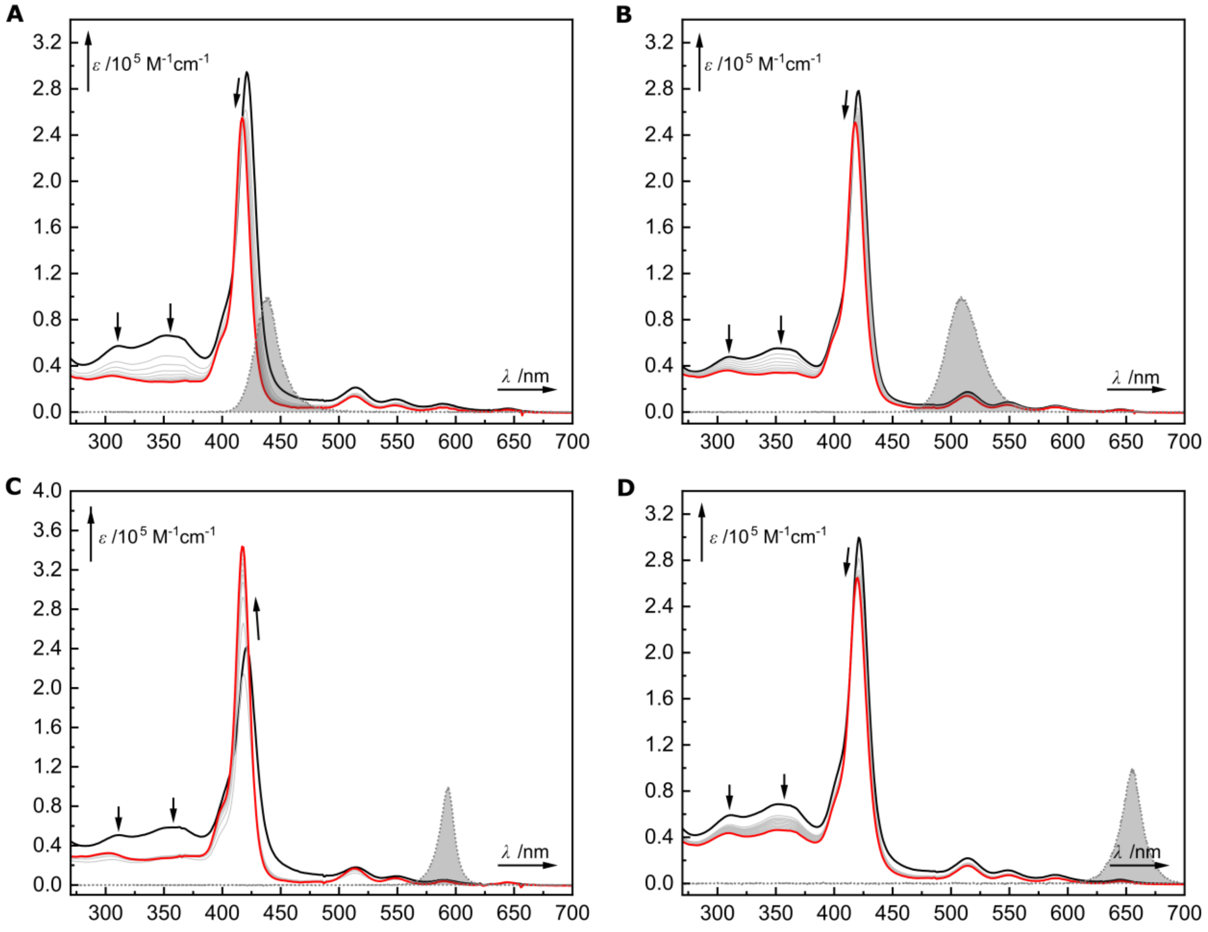

Figure S6. Irradiation of **2** in aerated methanol (c = 5 µM) at (A) 440 (~1 mW cm^{-2}), (B) 515 (~4 mW cm^{-2}), (C) 600 (~2.5 mW cm^{-2}), or (D) 650 nm (~2 mW cm^{-2}). UV-vis spectra were recorded every 60 min, and the spectra prior to (black line) and after (red line) 12 h irradiation are highlighted. The emission spectra of the irradiation sources are shown as a grey dotted line.

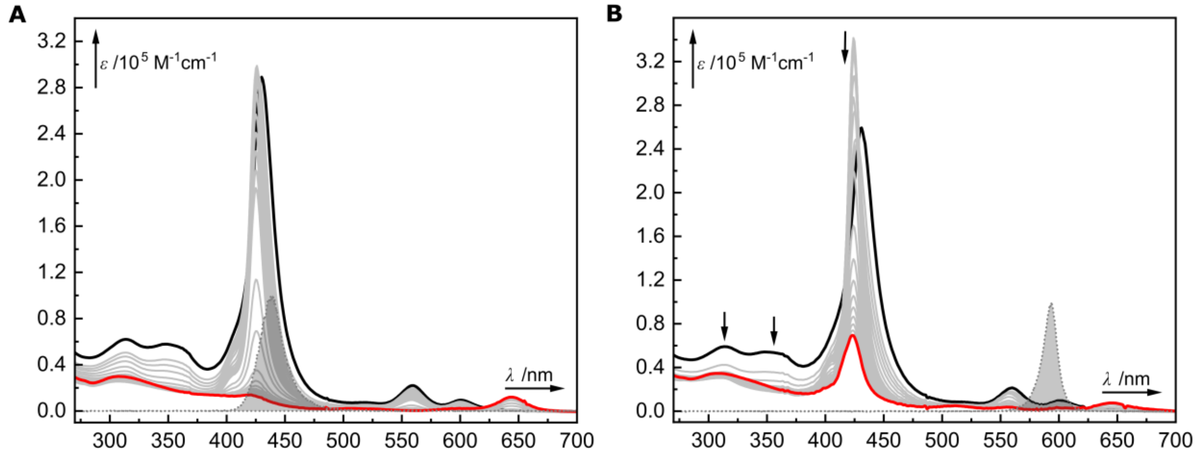

Figure S7. Irradiation of **2-Zn** in aerated methanol (c = 5 µM) at (A) 440 (~1 mW cm^{-2}), or (B) 600 (~2.5 mW cm^{-2}) nm. UV-vis spectra were recorded every 60 min, and the spectra prior to (black line)

and after (red line) 12 h irradiation are highlighted. The emission spectra of the irradiation sources are shown as a grey dotted line.

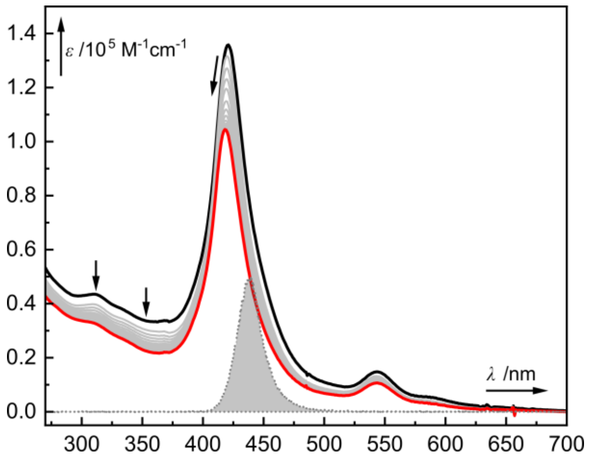

Figure S8. Irradiation of **2-Cu** in aerated methanol (c = 5 µM) at 440 nm (~1 mW cm^{-2}). UV-vis spectra were recorded every 60 min, and the spectra prior to (black line) and after (red line) 12 h irradiation are highlighted. The emission spectrum of the irradiation source is shown as a grey dotted line.

Photoirradiation Experiments in a Degassed Solution

The essential role of oxygen in the solution was confirmed by irradiating **3** in methanol/DMSO (4:1, v/v) after 4 cycles of freeze-pump-thaw degassing. The photodecomposition of the flavonol groups (detected at 355 nm) was 15 times slower (Figure S9), and the CO release efficiency was lower than that in an aerated solution by a factor of 6 (Figure S10).

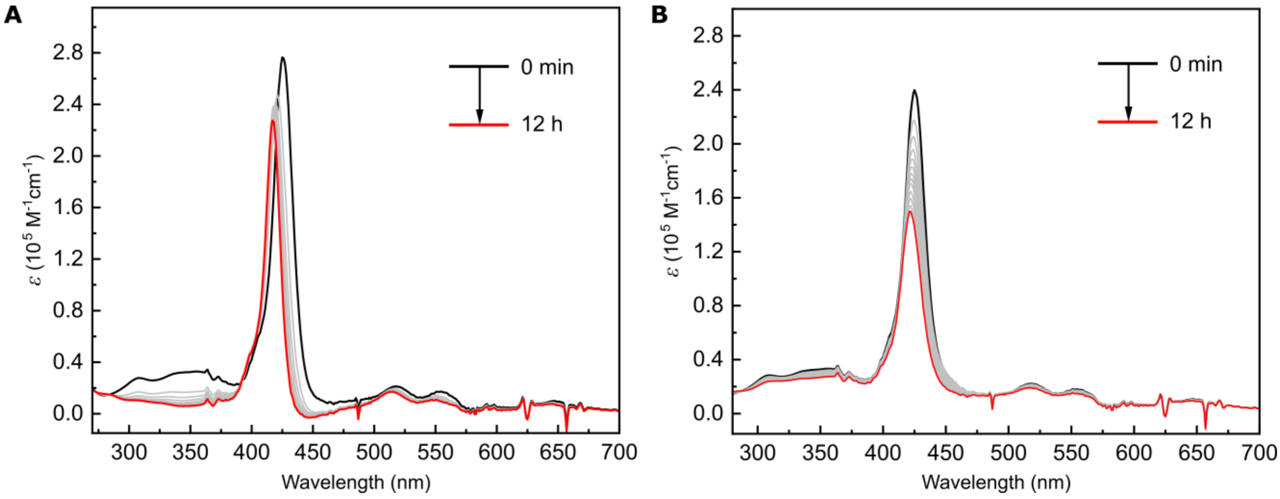

Figure S9. Irradiation of **3** (c = 5 µM) at 440 nm (~1 mW cm^{-2}) in (A) aerated, or (B) degassed methanol. UV-vis spectra were recorded every 60 min, and the spectra prior to (black line) and after (red line) 12 h irradiation are highlighted.

Kinetic of the CO Evolution from 3 in a Degassed Solution

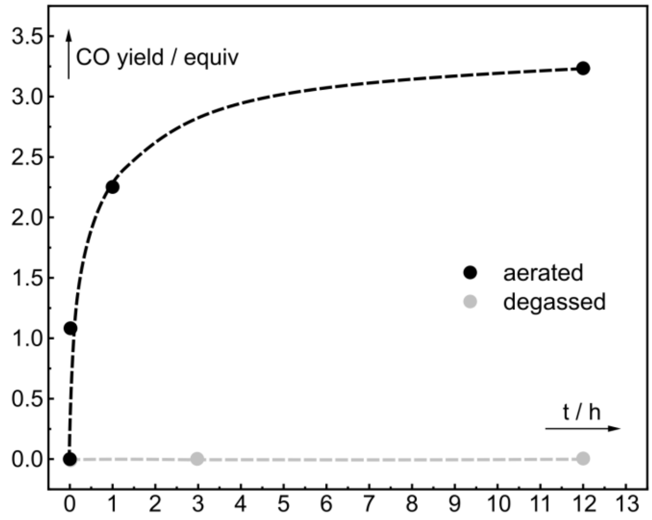

Figure S10. The kinetics of the CO evolution from **3** in an aerated (black) or degassed methanol/DMSO (4:1, v/v) solution (c = 5 μM) upon irradiation at 440 nm (~1 mW cm^{-2}). The CO released was determined by headspace GC-MS and is expressed as equivalents of CO released by 1 equiv of the photoCORM.

Photochemical Properties of 2, 2-Zn, and 2-Cu at Different Wavelengths

Table S6. Photochemical properties of compounds **2**, **2-Zn**, and **2-Cu**.

cmpd	λ_{abs} / nm (ε_{max} / 10^4 M^{-1} cm^{-1})[a]	CO yield[b] / equiv	Φ_{CO}[c]	$\Phi_{CO}\varepsilon_{max}$[d] / M^{-1} cm^{-1}
**2**	421 (31.3)	2.9 ± 0.1	0.018 ± 0.001	$(5.6 \pm 0.3) \times 10^3$
	514 (2.4)	2.9 ± 0.1	0.014 ± 0.001	$(3.4 \pm 0.2) \times 10^2$
	590 (0.9)	2.9 ± 0.1	0.016 ± 0.001	$(1.4 \pm 0.1 \times 10^2$
	645 (0.4)	2.6 ± 0.2	0.018 ± 0.001	$(7.0 \pm 0.2) \times 10^1$
**2-Zn**	431 (25.6)	2.1 ± 0.1	0.040 ± 0.002	$(1.0 \pm 0.1) \times 10^4$
	560 (2.6)	2.1 ± 0.1	0.035 ± 0.003	$(9.8 \pm 0.8) \times 10^2$
	601 (0.9)	2.1 ± 0.1	0.041 ± 0.006	$(3.8 \pm 0.5) \times 10^2$
**2-Cu**	421 (15.4)	0.8 ± 0.1	0.002 ± 0.001	$(2.9 \pm 0.8) \times 10^2$

All measurements were performed in methanol. [a] The molar absorption coefficient, ε_{max}. [b] The total chemical yield of released CO, monitored by GC headspace, obtained upon exhaustive irradiation at the corresponding absorption band, and expressed as equivalents of CO released by 1 equiv of the photoCORM. [c] Absolute quantum yield of CO release, determined using a Si-photodiode. [d] The CO uncaging cross-section at λ_{abs}: $\Phi_{CO}\varepsilon_{max}$.

Table S7. CO Production from **3** in the Presence of a $^1O_2$ Trap.

cmpd[a]	$^1O_2$ trap[b]	CO yield / equiv[c]
	-	2.3 ± 0.1
**3**	α-Terpinene	2.6 ± 0.1
	NaN_3	2.3 ± 0.1

[a] Methanol/DMSO (4:1, v/v) solution (c = 5 μM); irradiation at 440 nm (~1 mW cm^{-2}) for 2 h in the absence or presence of a $^1O_2$ trap. [b] c_{trap} ~ 5 mM. [c] The CO released was determined by headspace GC-MS and is expressed as equivalents of CO released by 1 equiv of the photoCORM.

Table S8. CO Production from **3** in the Presence of a Triplet Quencher.

cmpd[a]	triplet quencher[b]	CO yield / equiv[c]
	-	3.2 ± 0.3
**3**	NBA	2.6 ± 0.1
	COT	2.5 ± 0.1

[a] Methanol/DMSO (4:1, v/v) solution (c = 5 μM); irradiation at 440 nm (~1 mW cm^{-2}) for 2 h in the absence or presence of a triplet quencher. [b] NBA = 4-nitrobenzyl alcohol; COT = 1,3,5,7-cyclooctatetraene; c ~ 10 mM. [c] The CO released was determined by headspace GC-MS and is expressed as equivalents of CO released by 1 equiv of the photoCORM.

Emission Spectra of the Light Sources

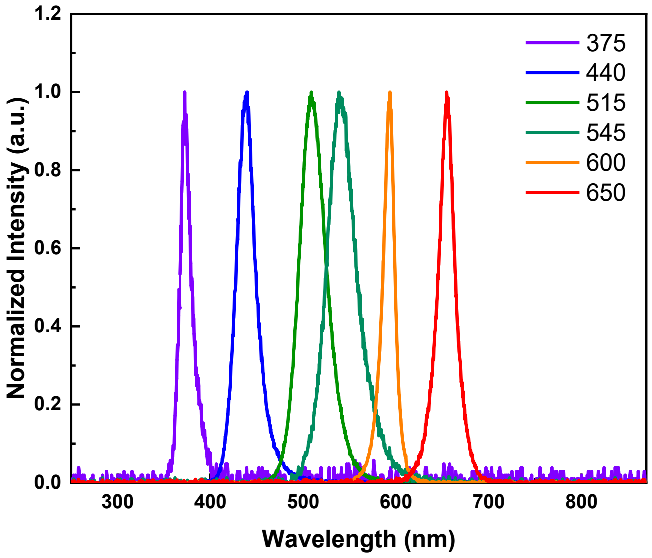

Figure S11. Normalized emission spectra of the LEDs used for irradiation. λ_{max} = 375 nm (~1 mW cm^{-2}); λ_{max} = 440 nm (~1 mW cm^{-2}); λ_{max} = 515 nm (~4 mW cm^{-2}); λ_{max} = 545 nm (~1.8 mW cm^{-2}); λ_{max} = 600 nm (~2.5 mW cm^{-2}); λ_{max} = 650 nm (~2 mW cm^{-2}).

Photoproducts Analyses

The products of flavonol moiety degradation formed upon irradiation of **3** at 440 nm (~1 mW cm^{-2}) detected by mass spectrometry (MALDI-TOF) were identified as the expected esters of the salicylic acid (Figure S12–13). There was an evident sequential photodecarbonylation in all four flavonol units, whereas the porphyrin core was found nearly intact even after prolonged irradiation, and no products of porphyrin photodegradation were detected.

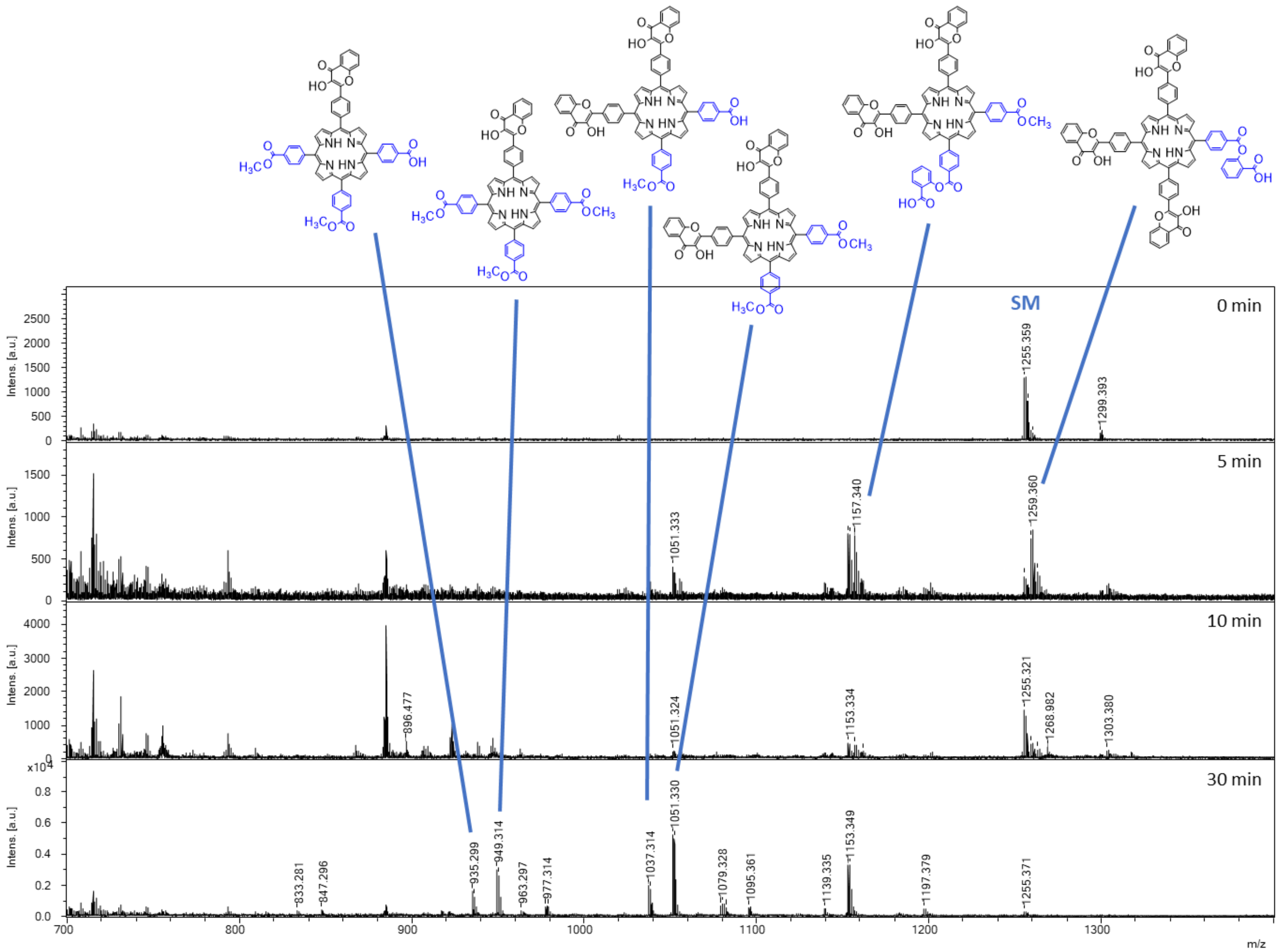

Figure S12. HRMS (MALDI+) of **3** in an aerated methanol/DMSO (4:1, v/v) solution (c ~ 5 μM) irradiated at 440 nm (~1 mW cm^{-2}) for 0–30 min.

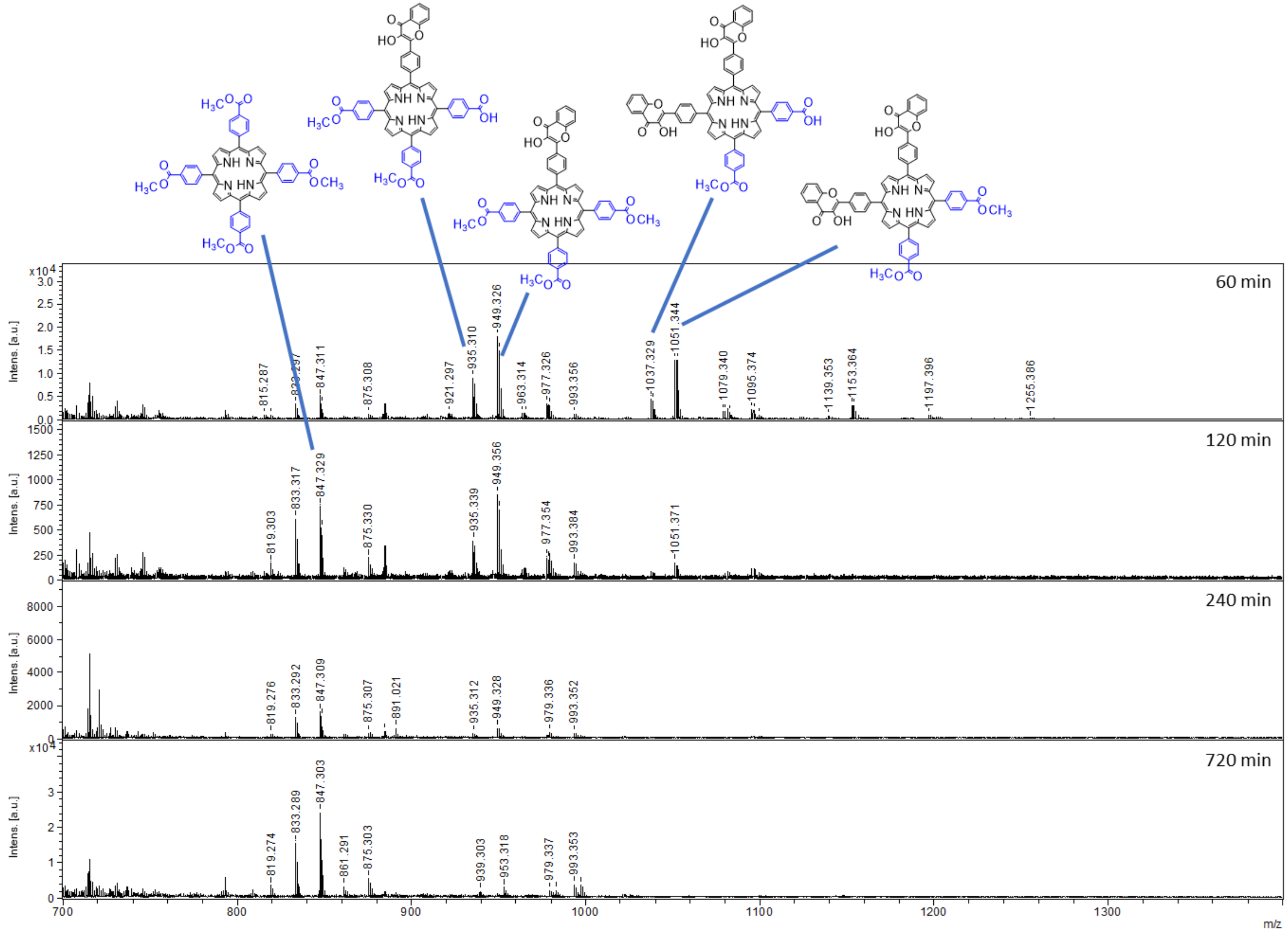

Figure S13. HRMS (MALDI+) of **3** in an aerated methanol/DMSO (4:1, v/v) solution (c ~ 5 μM) irradiated at 440 nm (~1 mW cm^{-2}) for 60–720 min.

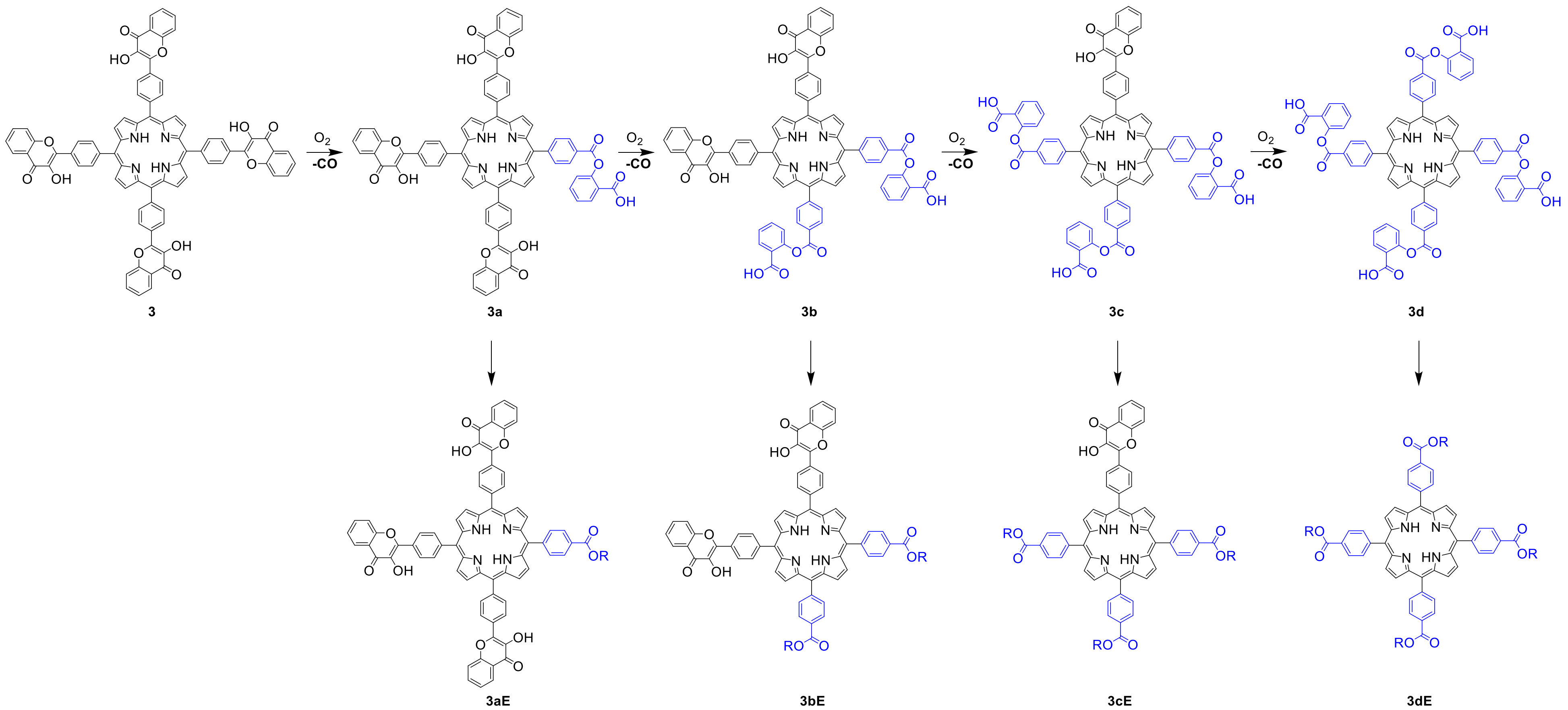

Scheme S6. Structures of the primary photoproducts obtained by irradiation of **3** in aerated methanol resulting in the release of CO (**3a-3d**). Benzoate ester hydrolysis (R = OH) or transesterification with the solvent (R = OCH_3) in the dark yielded products **3aE–3dE**. The structures with mixed functionalities or possible regioisomers are not shown.

Transient Absorption Spectroscopy

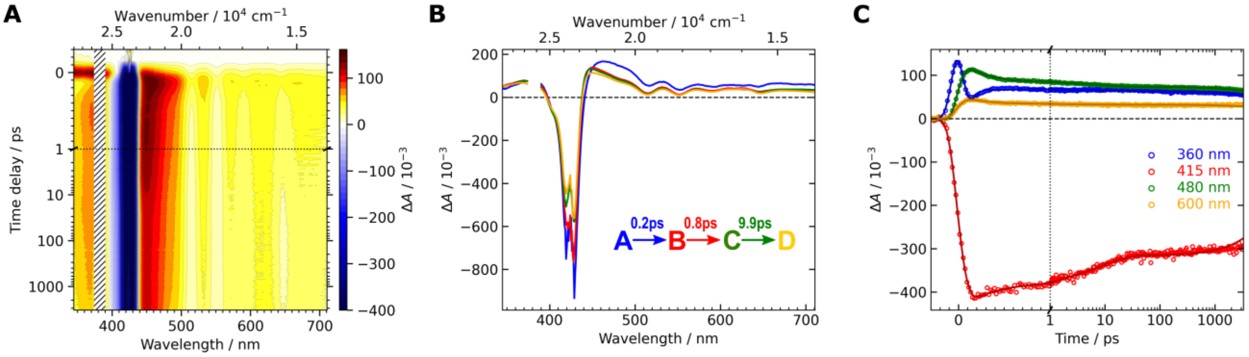

Figure S14. (A) Femtosecond (fs) transient absorption (TA) spectra (with chirp correction) in degassed DMSO (λ_{ex} = 387 nm), (B) evolution-associated spectra (EAS) resulting from global analysis, and (C) the fitted traces at the selected wavelengths for **3**.

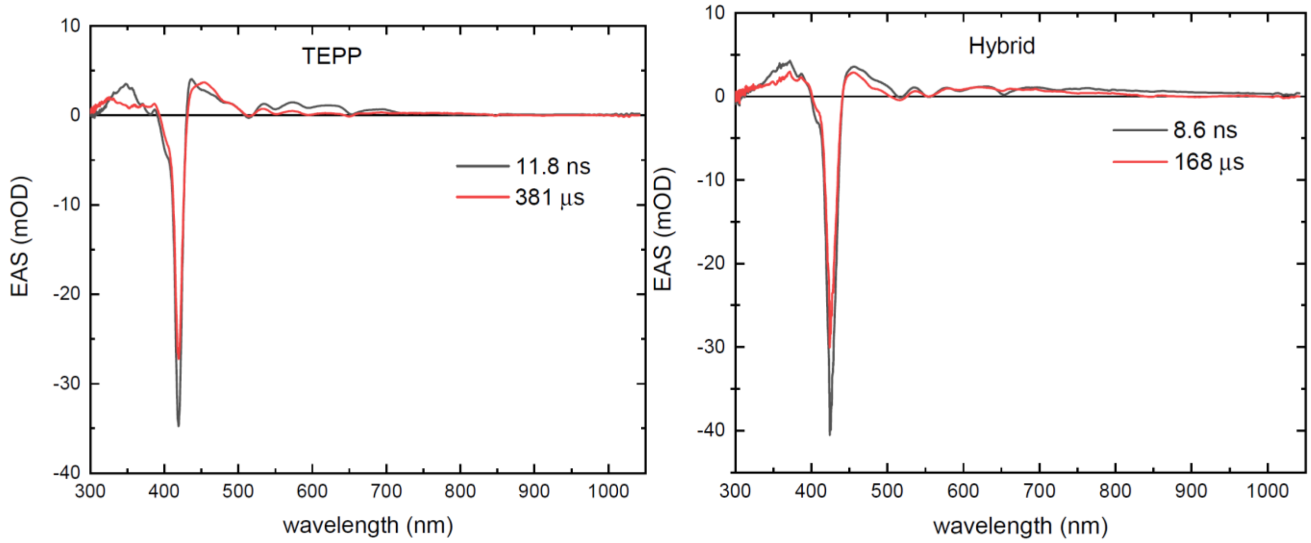

Figure S15: Evolution associated spectra of TEPP (left) and hybrid **3** (right) after excitation at 520 nm in a degassed THF solution. The faster components (11.8, and 8.6 ns, black) can be associated with intersystem crossing; the longer ones (381, and 168 µs, red) with the triplet state lifetimes.

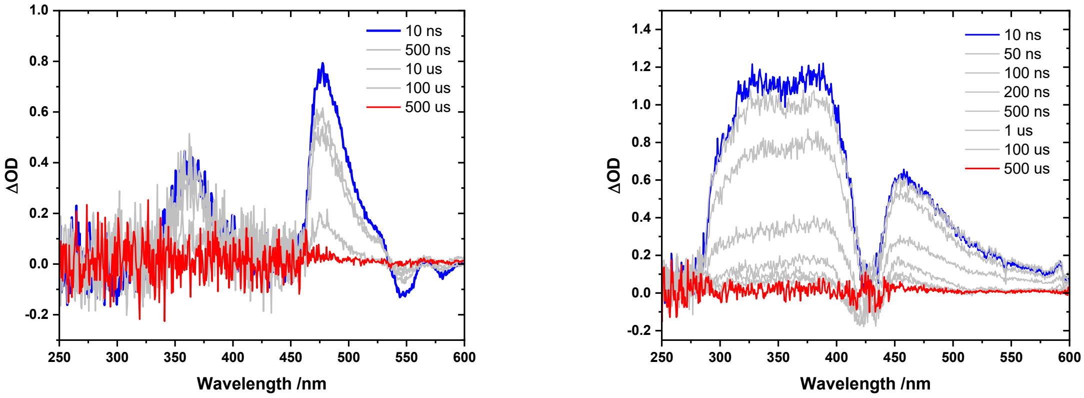

Figure S16. Nanosecond transient absorption spectra of tetraphenylporphyrin (TPP; left) and **3** (right; *c* ~20 µM) in degassed THF solution (λ_{ex} = 532 nm).

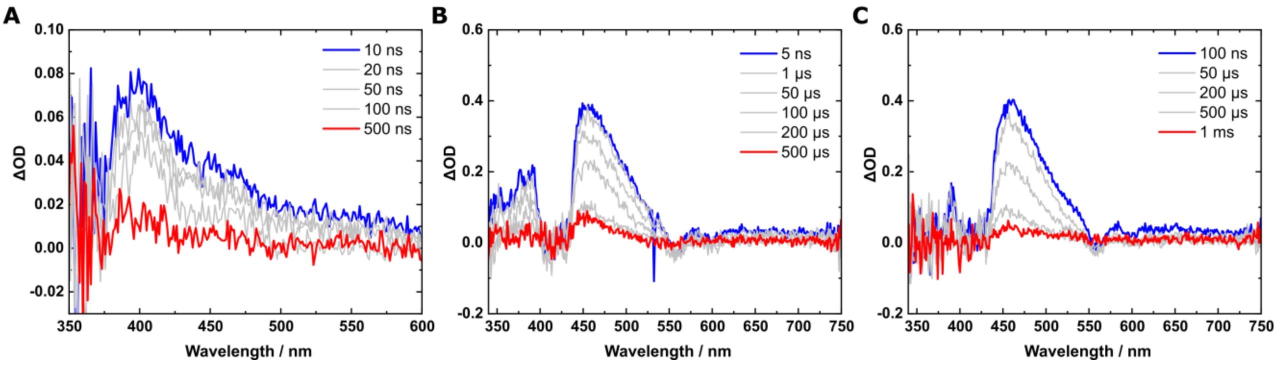

Figure S17. Nanosecond transient absorption spectra in degassed methanol of (A) 3-hydroxyflavone (**1**, 100 µM, λ_{ex} = 355 nm), (B) ZnTcPP (20 µM, λ_{ex} = 532 nm), and (C) a ZnTcPP and **1** mixture (c_{ZnTcPP} = 20 µM, c_1 = 500 µM; λ_{ex} = 532 nm).

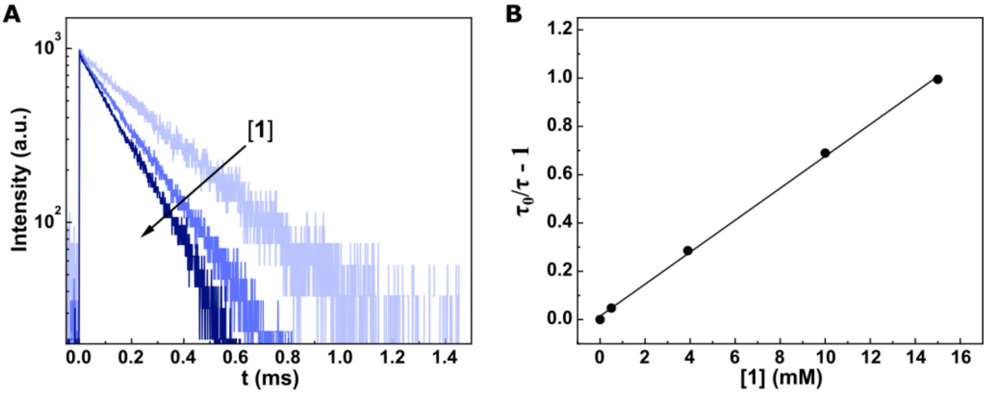

Figure S18. (A) Triplet decay kinetics of Zn-TcPP as a function of the **1** concentration (degassed methanol, c_{ZnTcPP} =10 µM, c_1 = 0–15 mM, λ_{ex} = 532 nm), and (B) the resulting Stern-Volmer plot in the 0–15 mM range.

$^1O_2$ Production of 2 After Irradiation

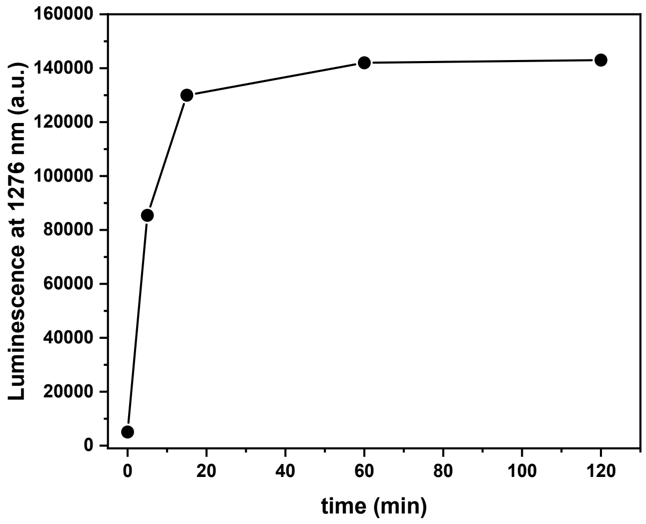

Figure S19. The effect of irradiation time on the singlet oxygen production by **2** sensitization. The $^1O_2$ luminescence signal (1276 nm) from a solution of **2** (c ~ 30 µM) in methanol was obtained after the irradiation of the sample with white light (~7 mW cm^{-2}, measured at 500 nm) for a different amount of time. The increasing signal shows that the photoproducts arising from **2** are better $^1O_2$ generators than **2**.

In Vitro Biological Studies

Cytotoxicity

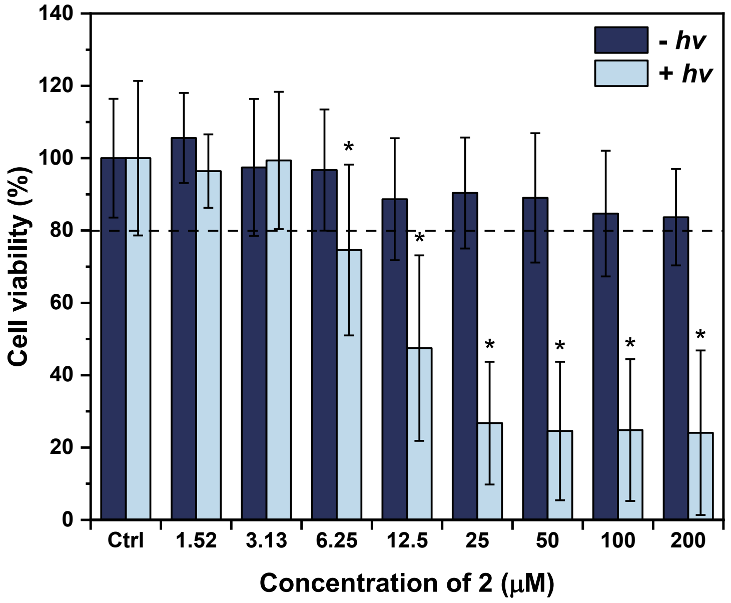

Figure S20. The effect of **2** on the viability of HepG2 cells. Cytotoxicity was assessed by an MTT test after treatment of HepG2 cells with solutions of **2** in MEM (1% DMSO) for 2 h without (- *hv*) or with (+ *hv*) white light irradiation (120 mW cm^{-2}). The values are expressed as % of untreated controls (Ctrl). * P-value ≤ 0.05 vs. untreated control; n ≥ 8.

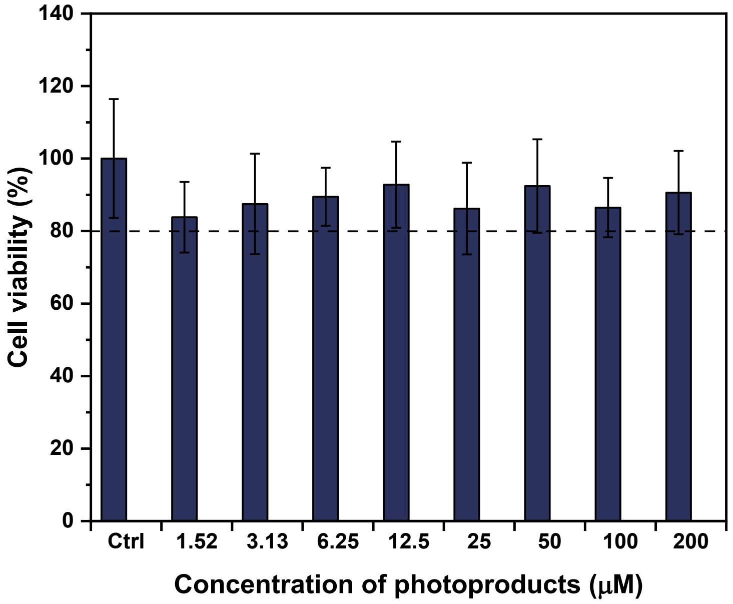

Figure S21. The effect of photoproducts formed from **2** on the viability of HepG2 cells. A solution of **2** was exhaustively irradiated with white light (990 mW cm^{-2}) for 48 h. Cytotoxicity was assessed by MTT test after treatment of HepG2 cells with solutions of the photoproducts in MEM (1% DMSO) for 24 h. The values are expressed as % of untreated control (Ctrl).

CO Release Kinetics

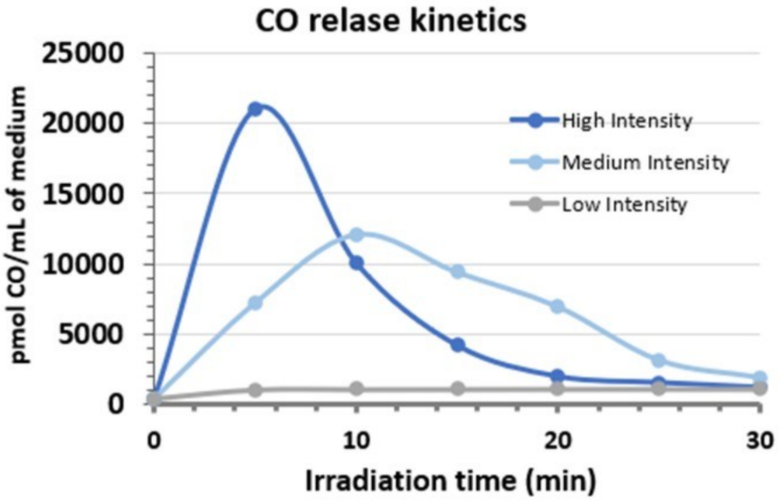

Figure S22. CO concentration and kinetics of CO release into the cell culture medium following irradiation with white light at high (990 mW cm^{-2}), medium (120 mW cm^{-2}), or low (8 mW cm^{-2}) intensity for 30 min. The concentration of compound **2** in these experiments was maintained at 25 µM.

Determination of CO Concentration in Cell Medium

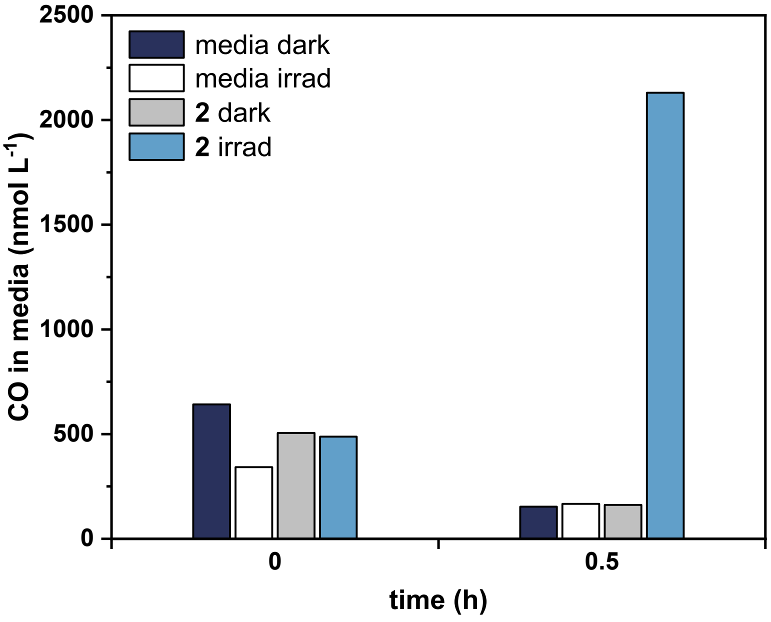

Figure S23. CO release from an irradiated sample of **2** into the cell culture medium. The CO concentration in a colorless MEM solution without **2** (media) or with **2** (25 µM) was measured following incubation in the dark (dark) or irradiation of HepG2 cells with white light (irrad.; 120 mW cm^{-2}) after 0 and 0.5 h using GC/RGA.

High-Resolution Respirometry

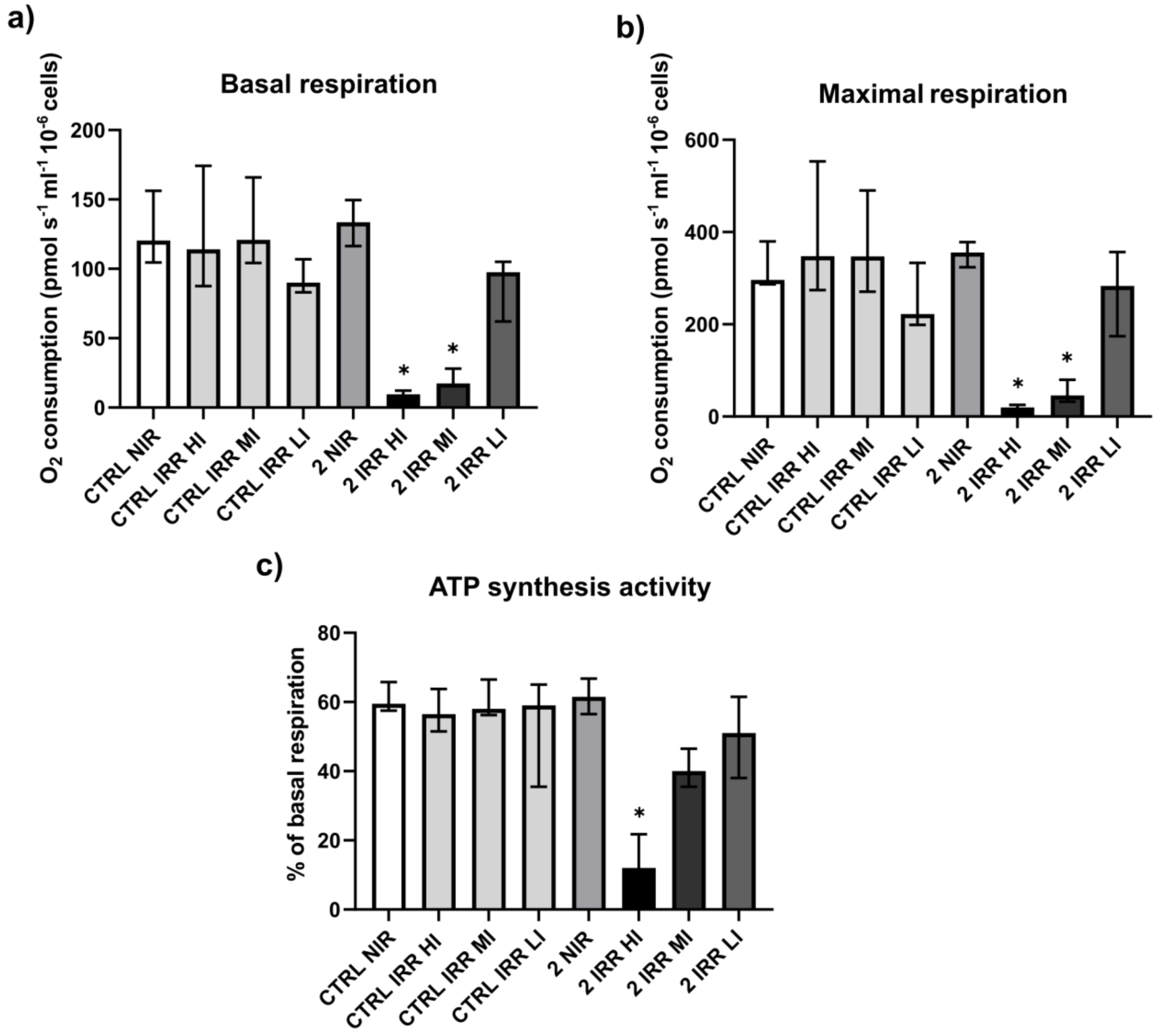

Figure S24. The effect of **2** on mitochondrial functions of HepG2 cells. Effect of irradiated (IRR) and non-irradiated (NIR) **2** solutions (25 µM) on the level of (a) basal and (b) maximum respiration, together with (c) ATP synthesis activity. Treated cells were irradiated with white light at high (HI), medium (MI), or low (LI) intensity (IRR; 990, 120, or 8 mW cm^{-2}, respectively) or kept in the dark (NIR) for 30 min and immediately analyzed; (a, b) the values are expressed as O_2 flux per mL of cell suspension and per 10^6 cells; (c) the ATP synthesis activity was calculated as the ratio of basal respiration to respiration after oligomycin inhibition and expressed as % of basal respiration level of untreated controls. * P-value ≤ 0.05; n ≥ 4).

Fluorescence Microscopy

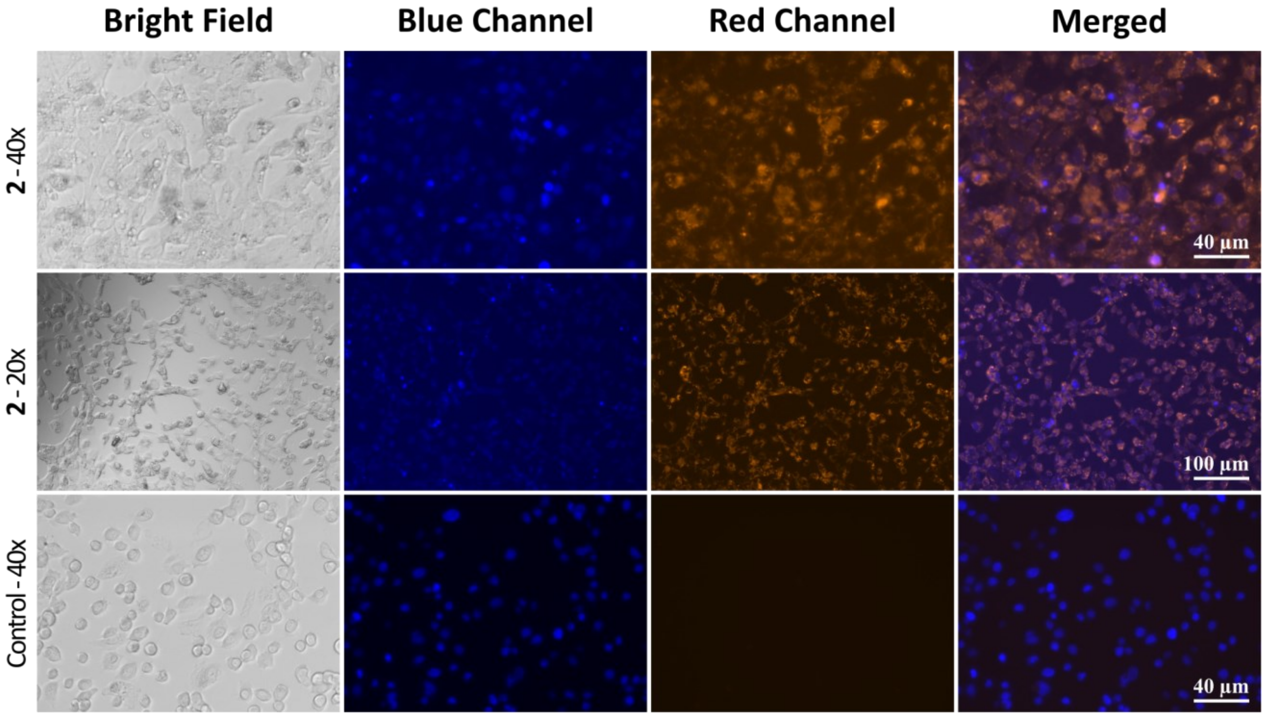

Figure S25. Fluorescence microscopy images of HepaRG cells treated with **2** (*c* ~ 100 µM) for 24 h. After incubation, the cells were washed with PBS and visualized using white light (bright field) and a WIG filter (red channel). Nuclei were counterstained with DAPI and observed under UV light (blue channel). Control cells were incubated in MEM without **2**.

NMR spectra

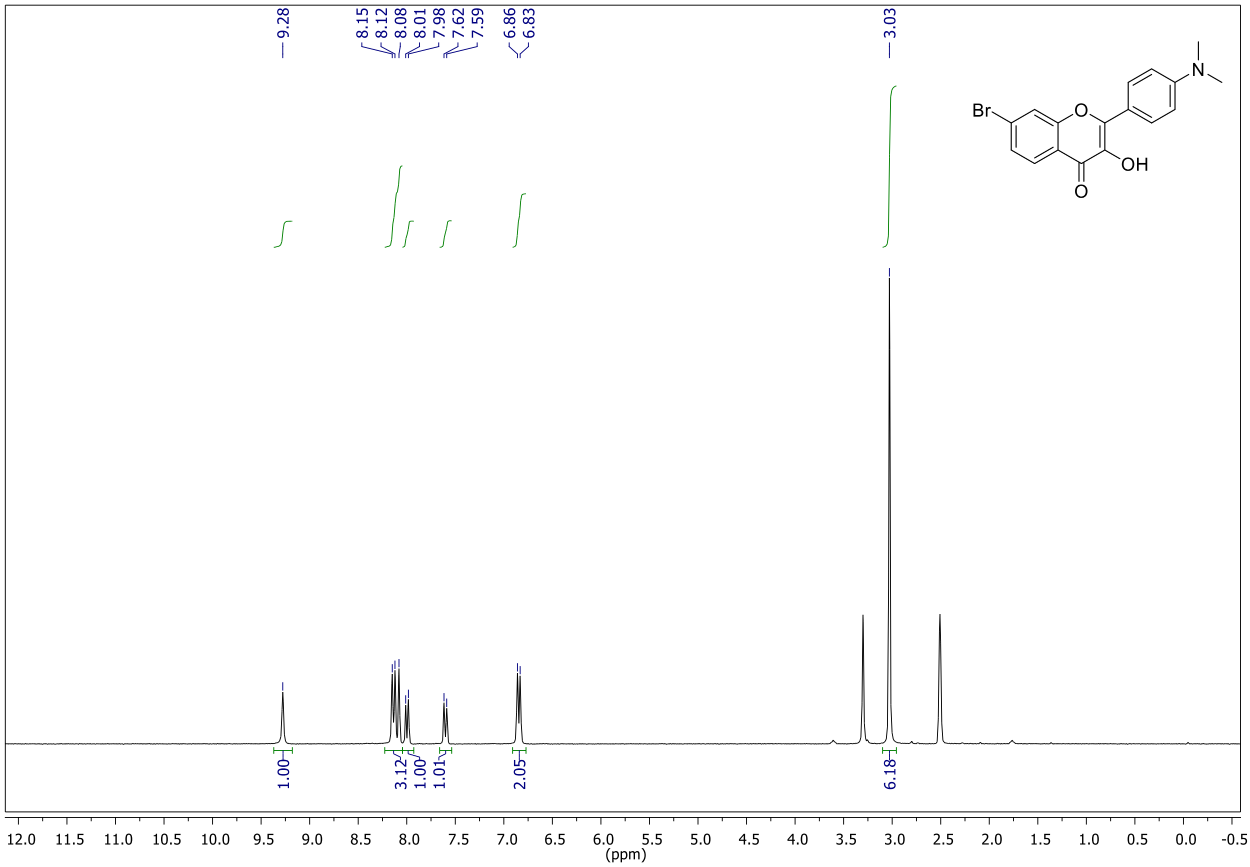

Figure S26. ^{1}H NMR (500 MHz, d_6-DMSO): **6**.

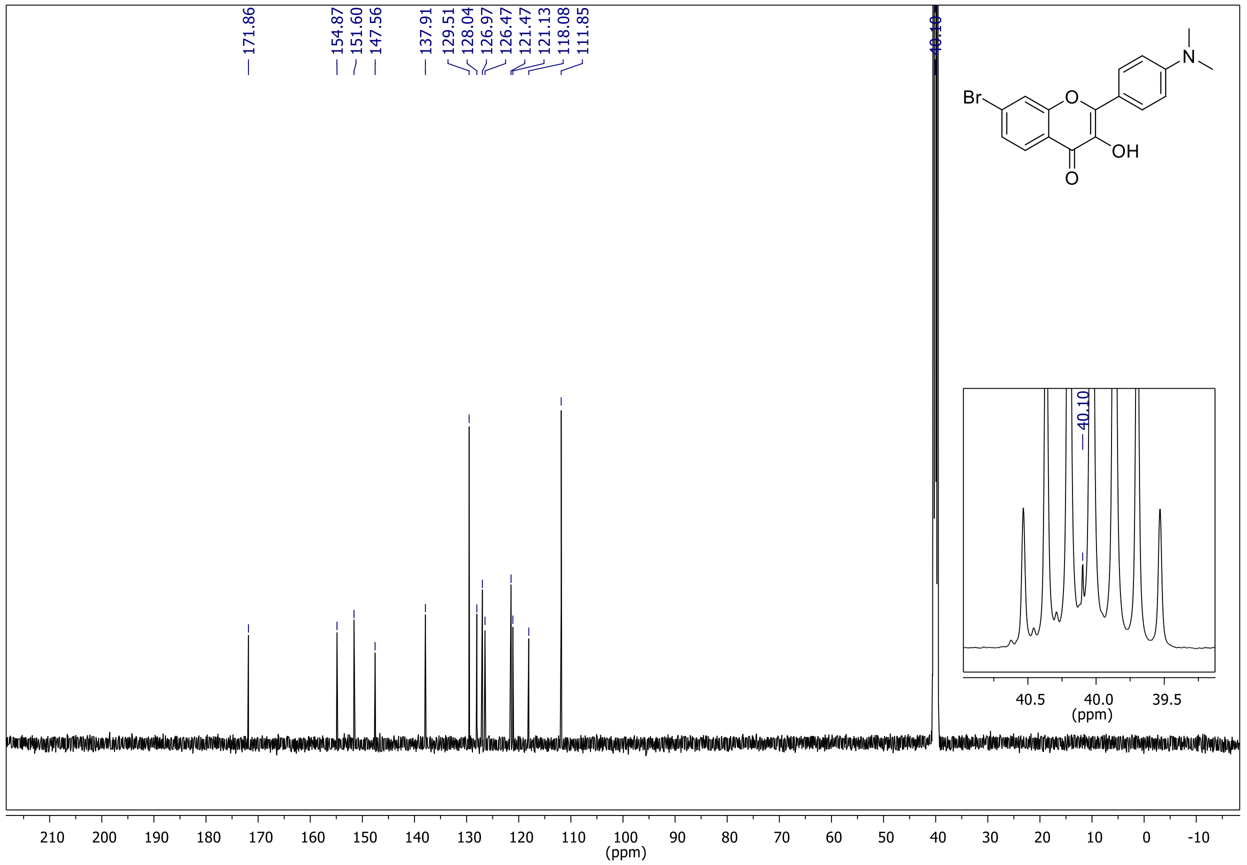

Figure S27. ^{13}C NMR (125 MHz, d_6-DMSO): **6**.

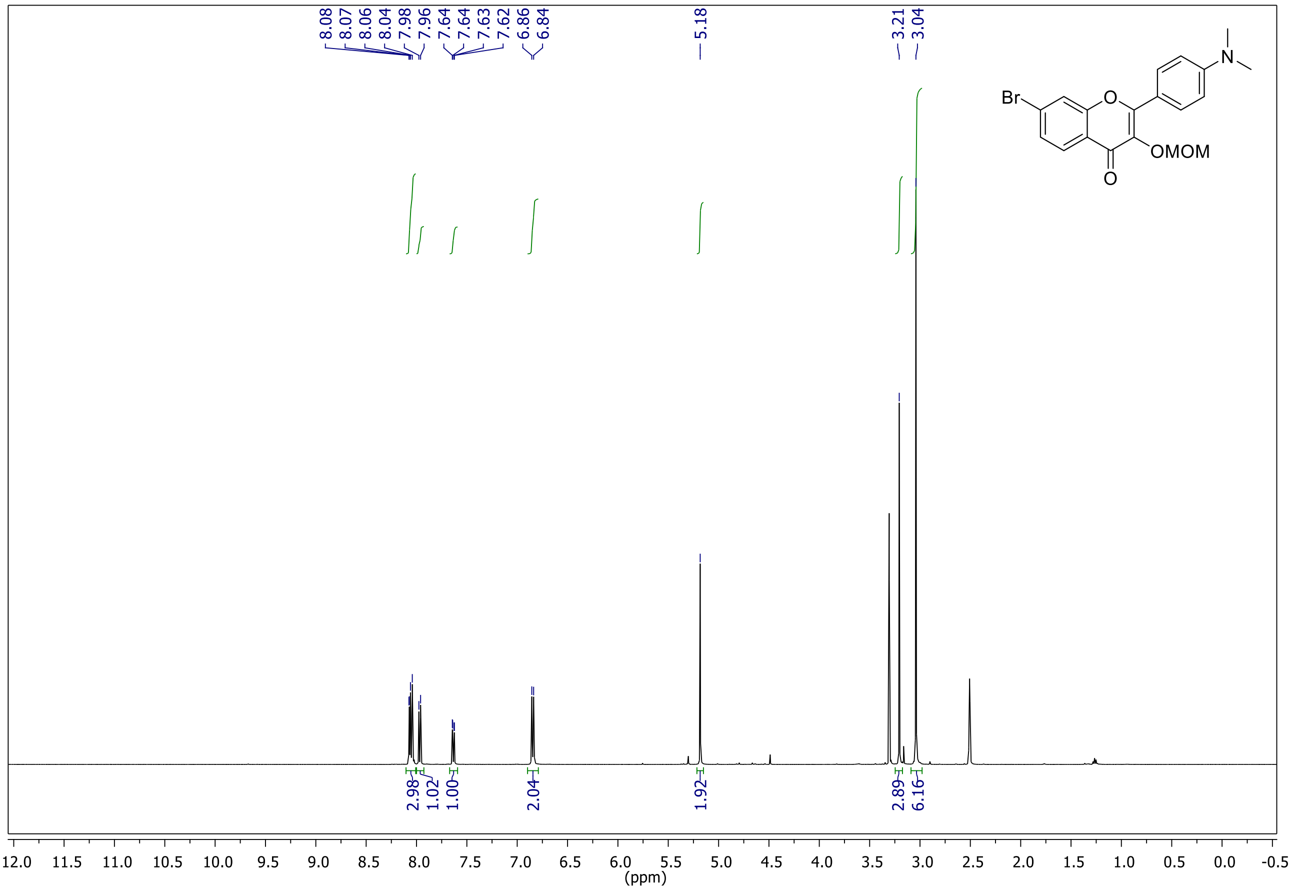

Figure S28. ^{1}H NMR (500 MHz, d_6-DMSO): **12**.

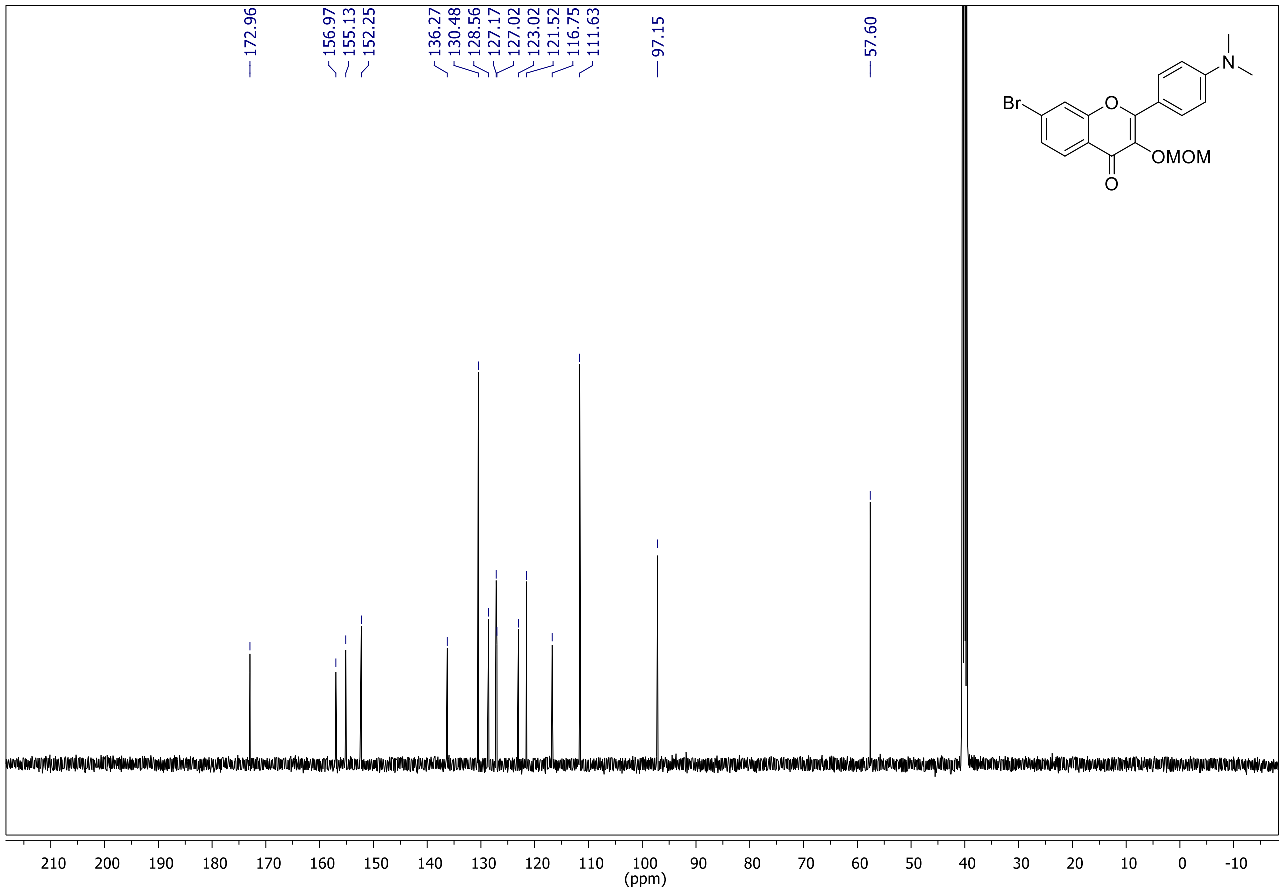

Figure S29. ^{13}C NMR (125 MHz, d_6-DMSO): **12**.

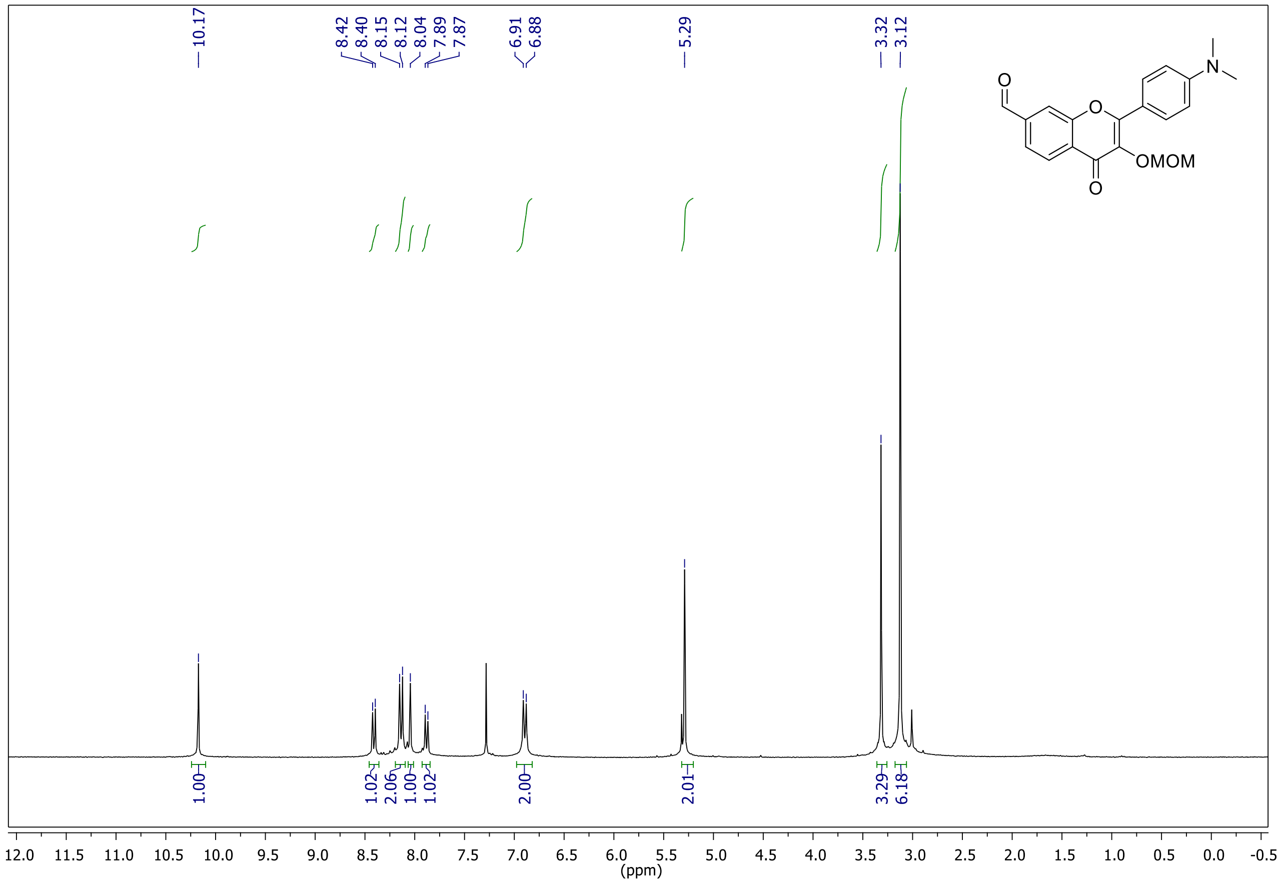

Figure S30. ^{1}H NMR (500 MHz, $CDCl_3$): **7**.

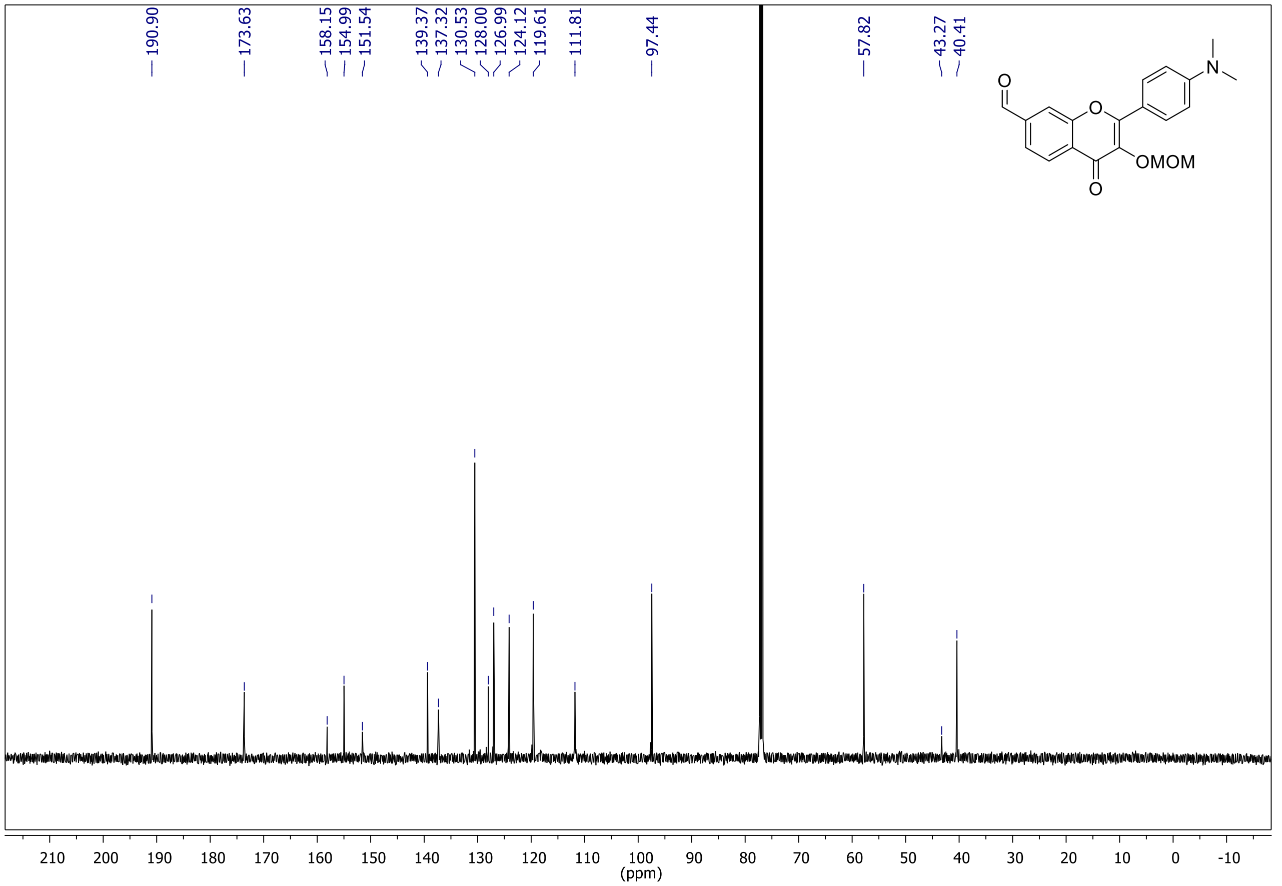

Figure S31. ^{13}C NMR (125 MHz, $CDCl_3$): **7**.

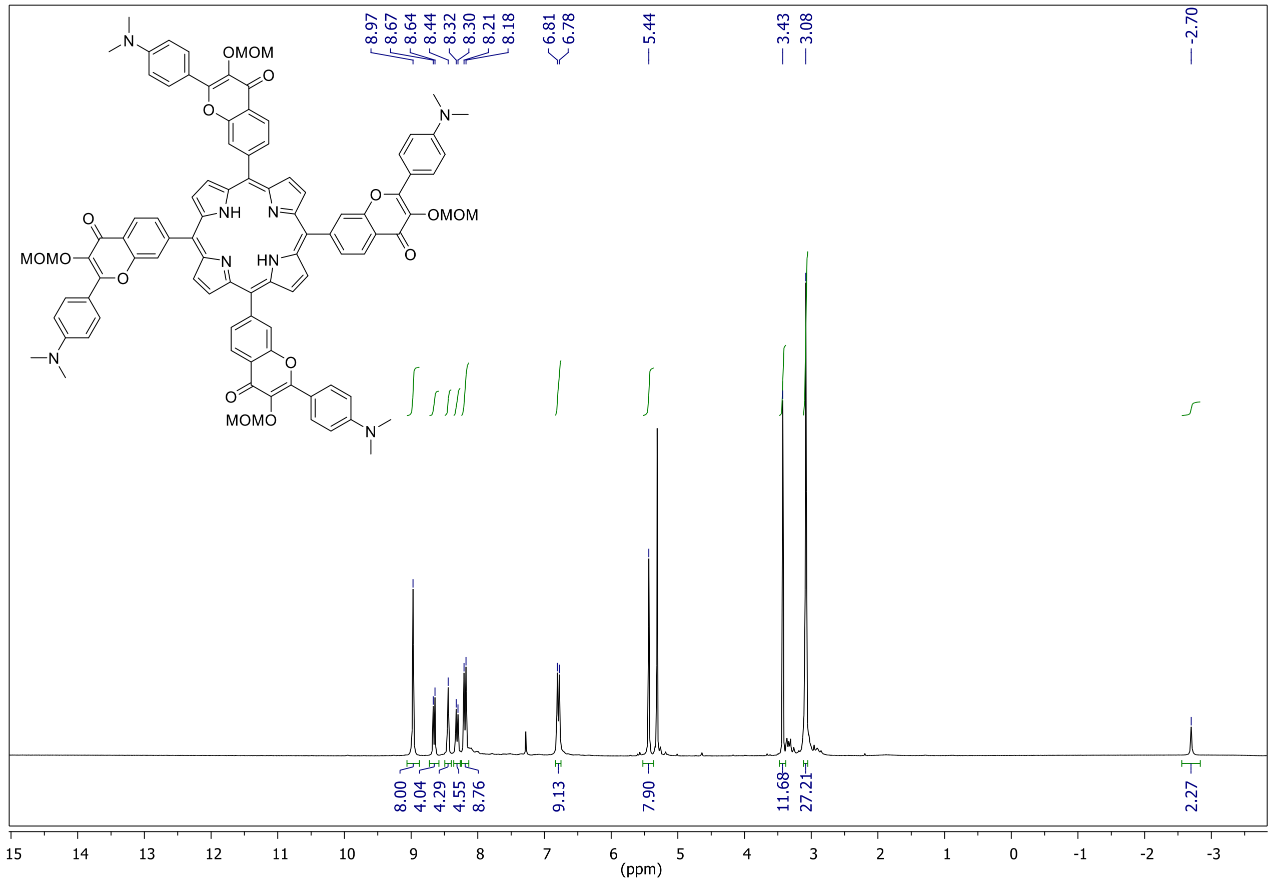

Figure S32. ^{1}H NMR (500 MHz, $CDCl_3$): **8**.

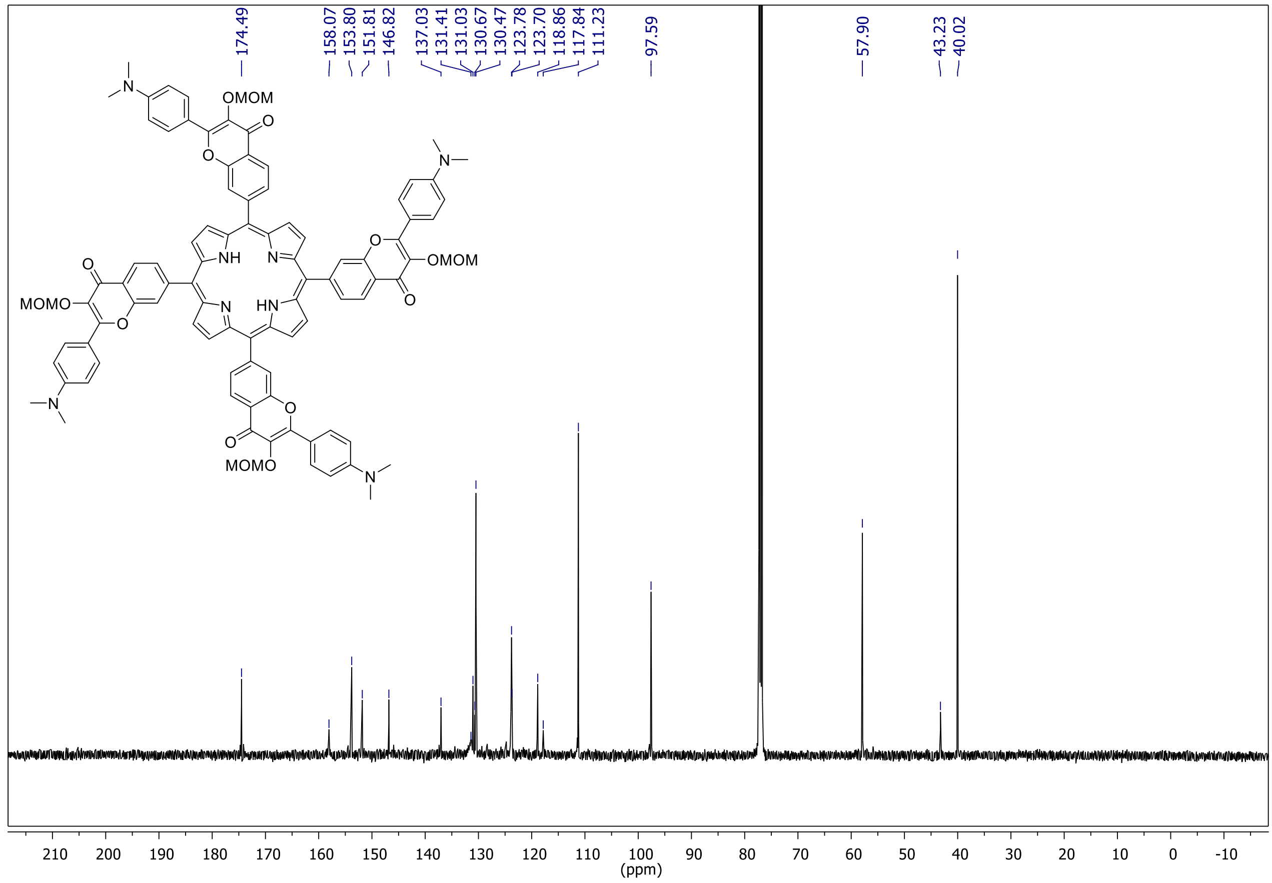

Figure S33. ^{13}C NMR (125 MHz, $CDCl_3$): **8**.

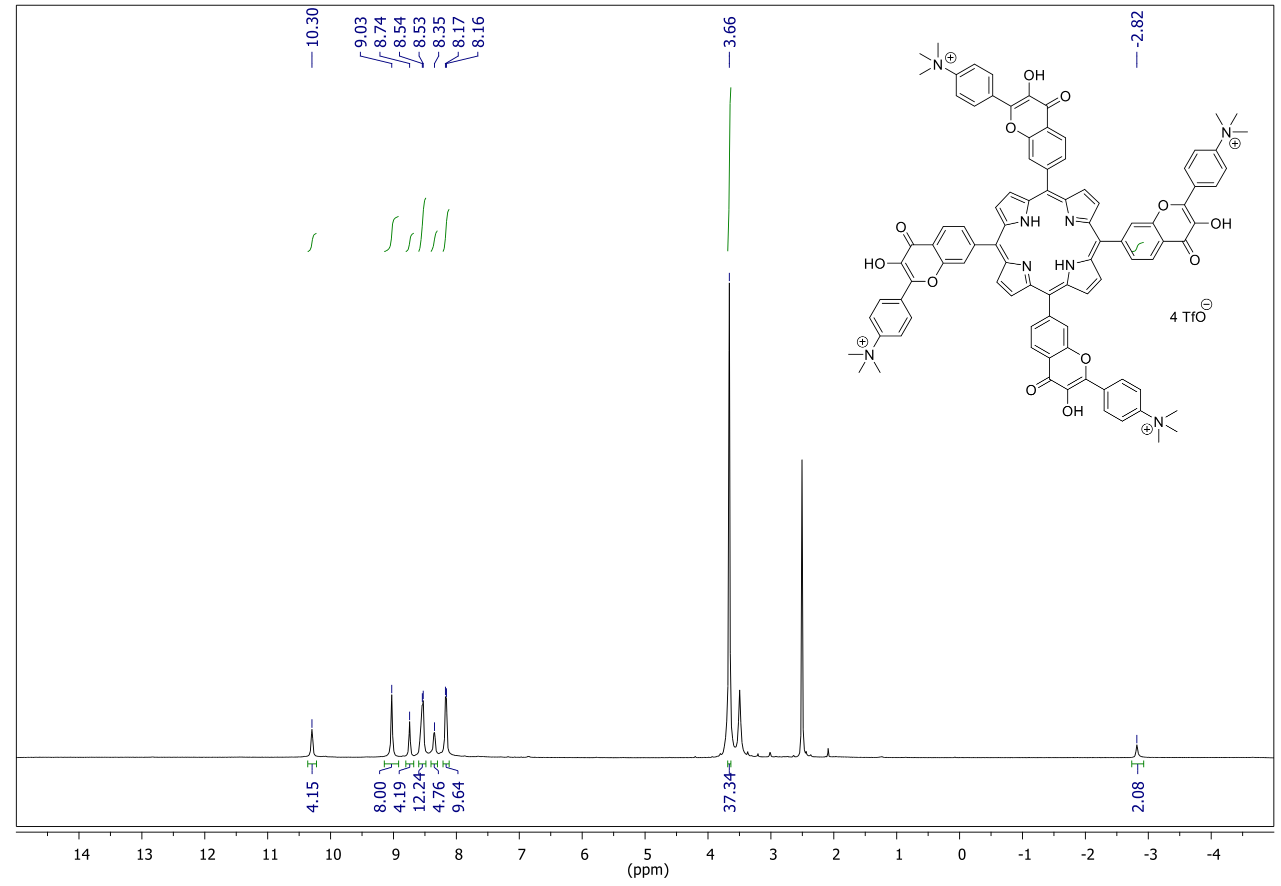

Figure S34. ^{1}H NMR (500 MHz, d_6-DMSO): **2**.

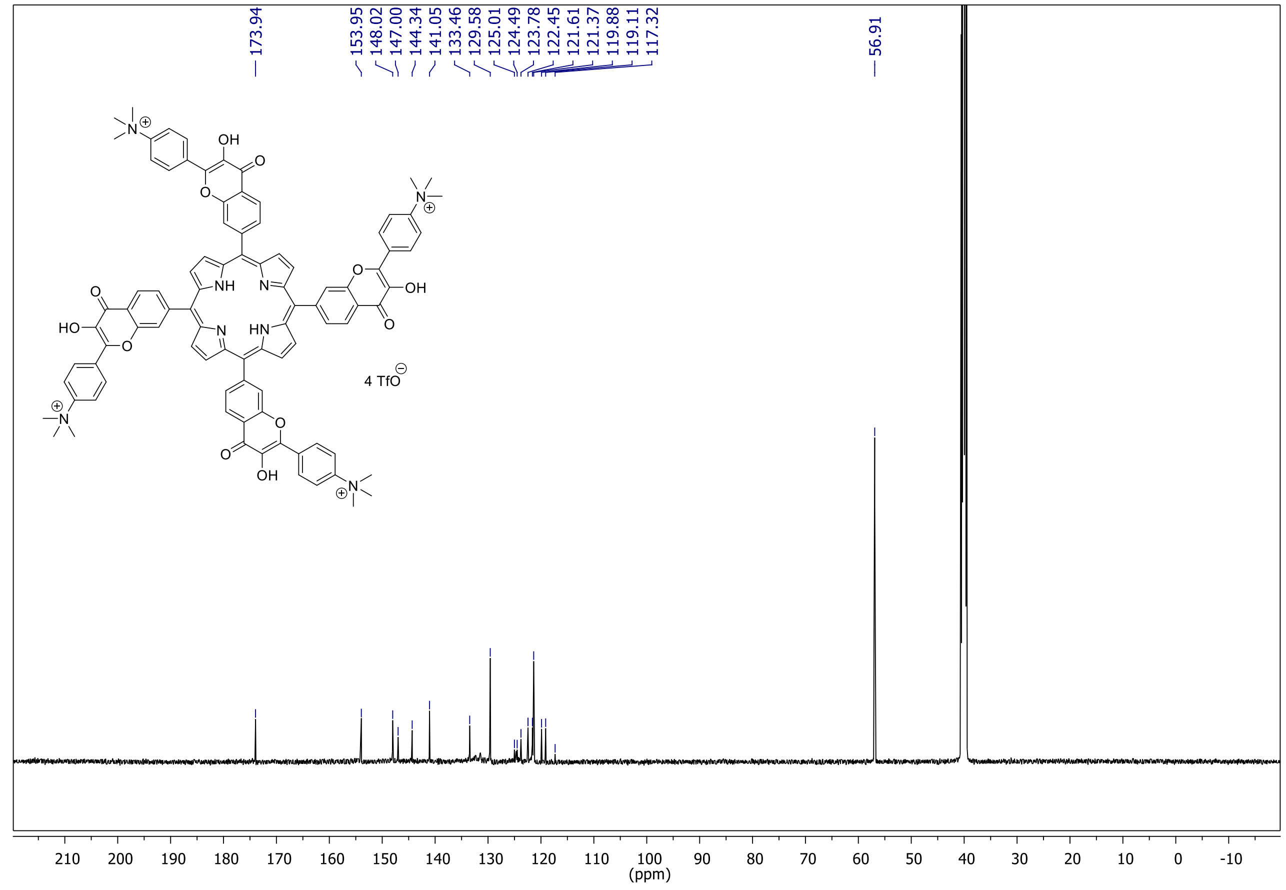

Figure S35. ^{13}C NMR (125 MHz, d_6-DMSO): **2**.

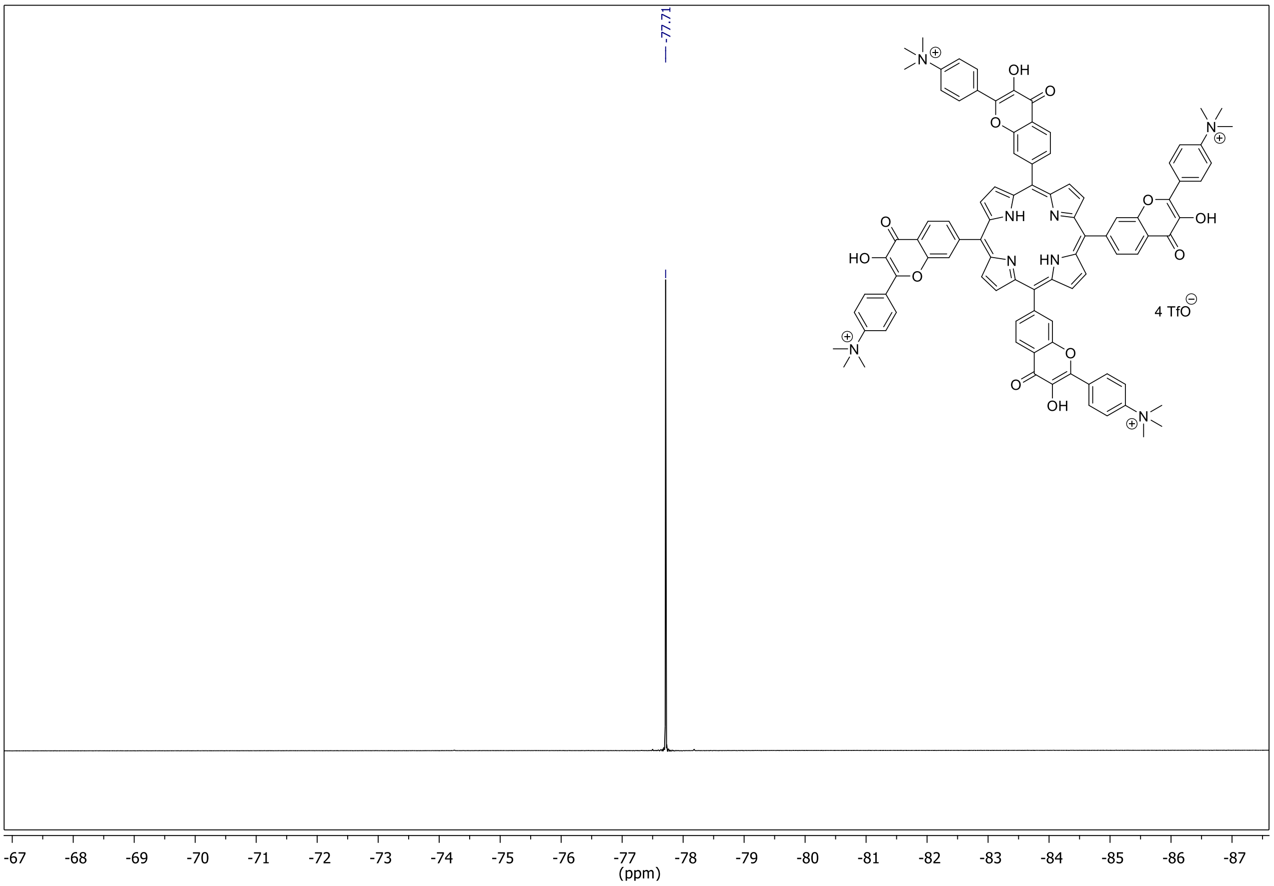

Figure S36. ^{19}F NMR (476 MHz, d_6-DMSO): **2**.

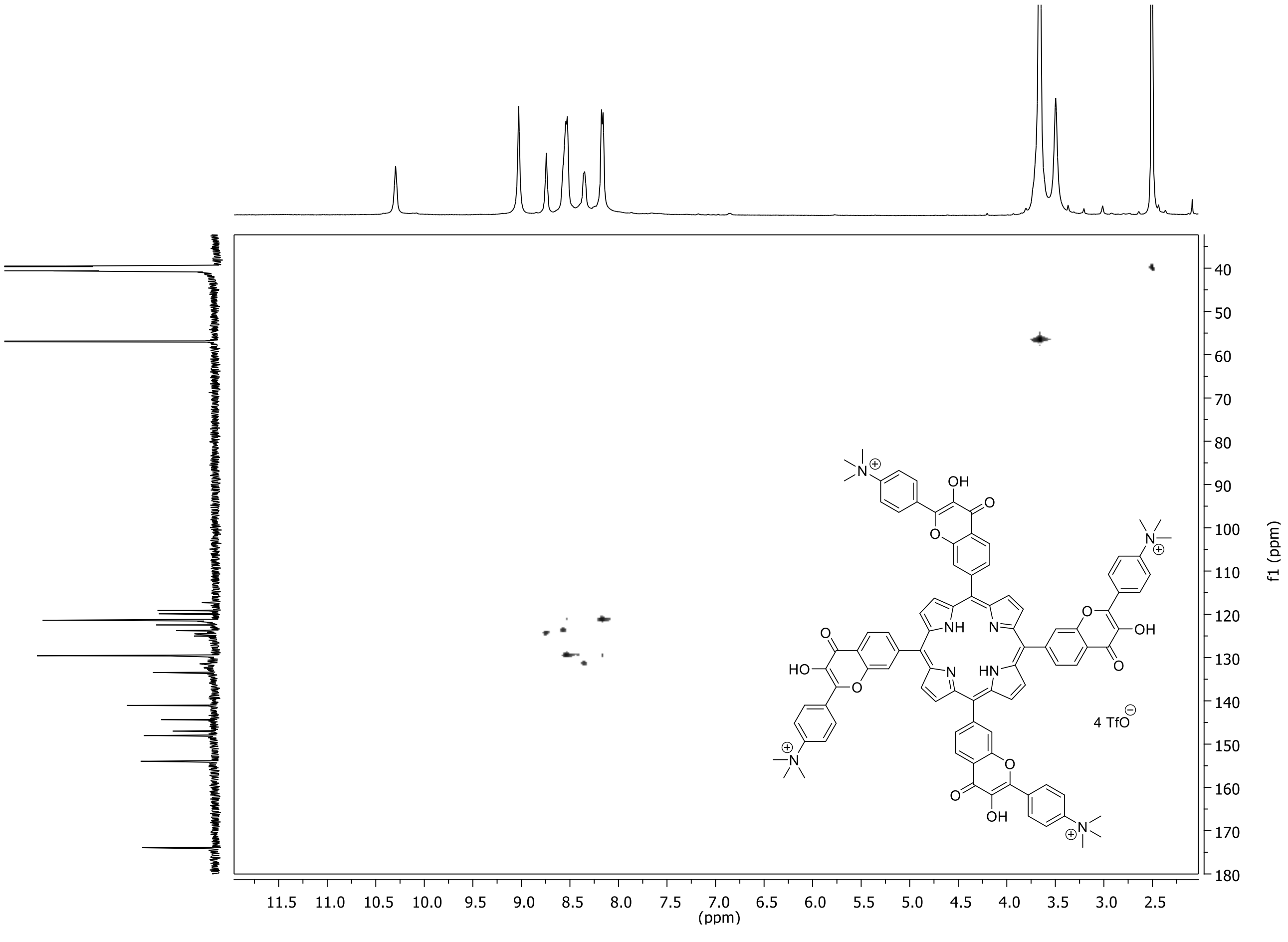

Figure S37. ^{1}H–^{13}C HSQC (500 MHz, d_6-DMSO): **2**.

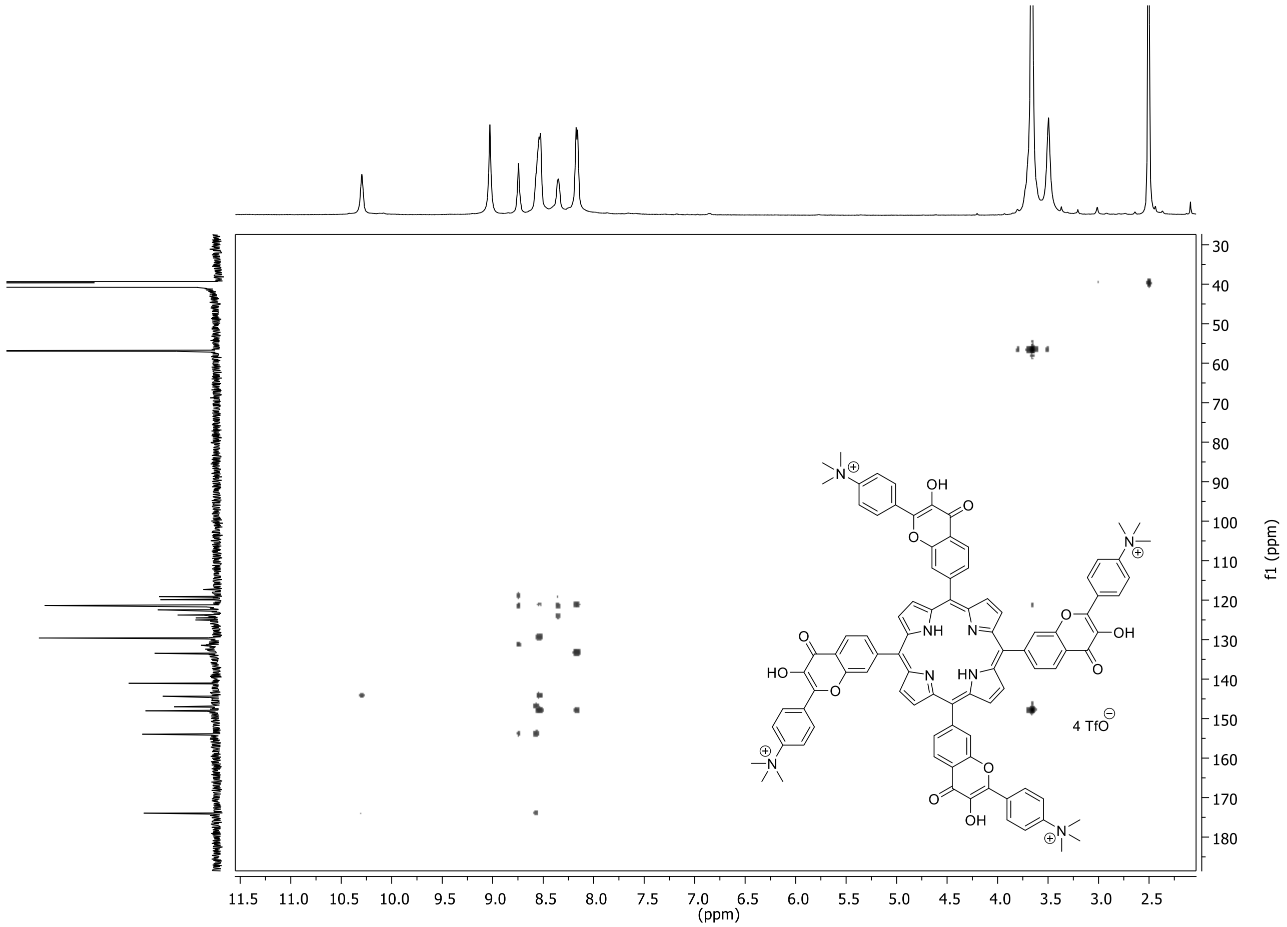

Figure S38. ^{1}H–^{13}C HMBC (500 MHz, d_6-DMSO): **2**.

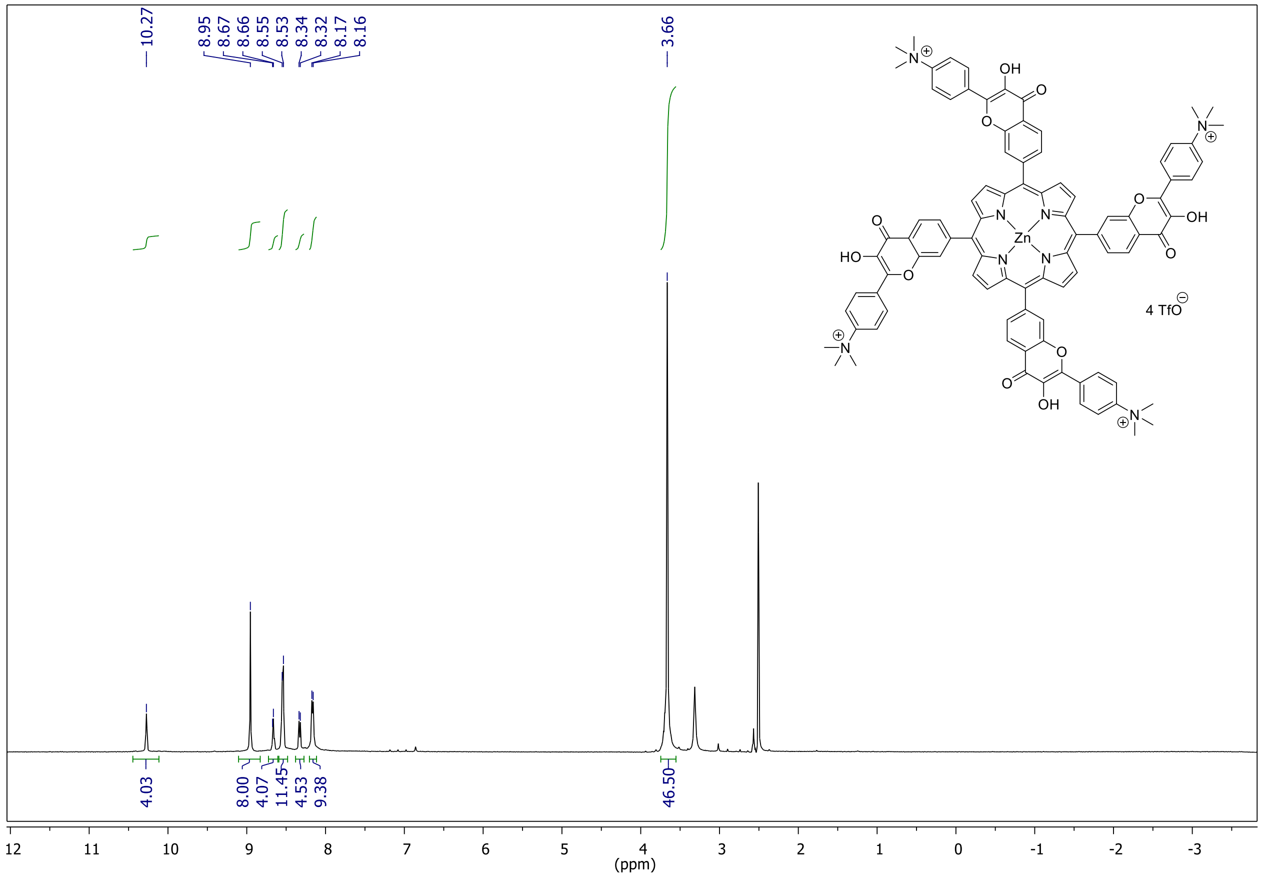

Figure S39. ^{1}H NMR (500 MHz, d_6-DMSO): **2-Zn**.

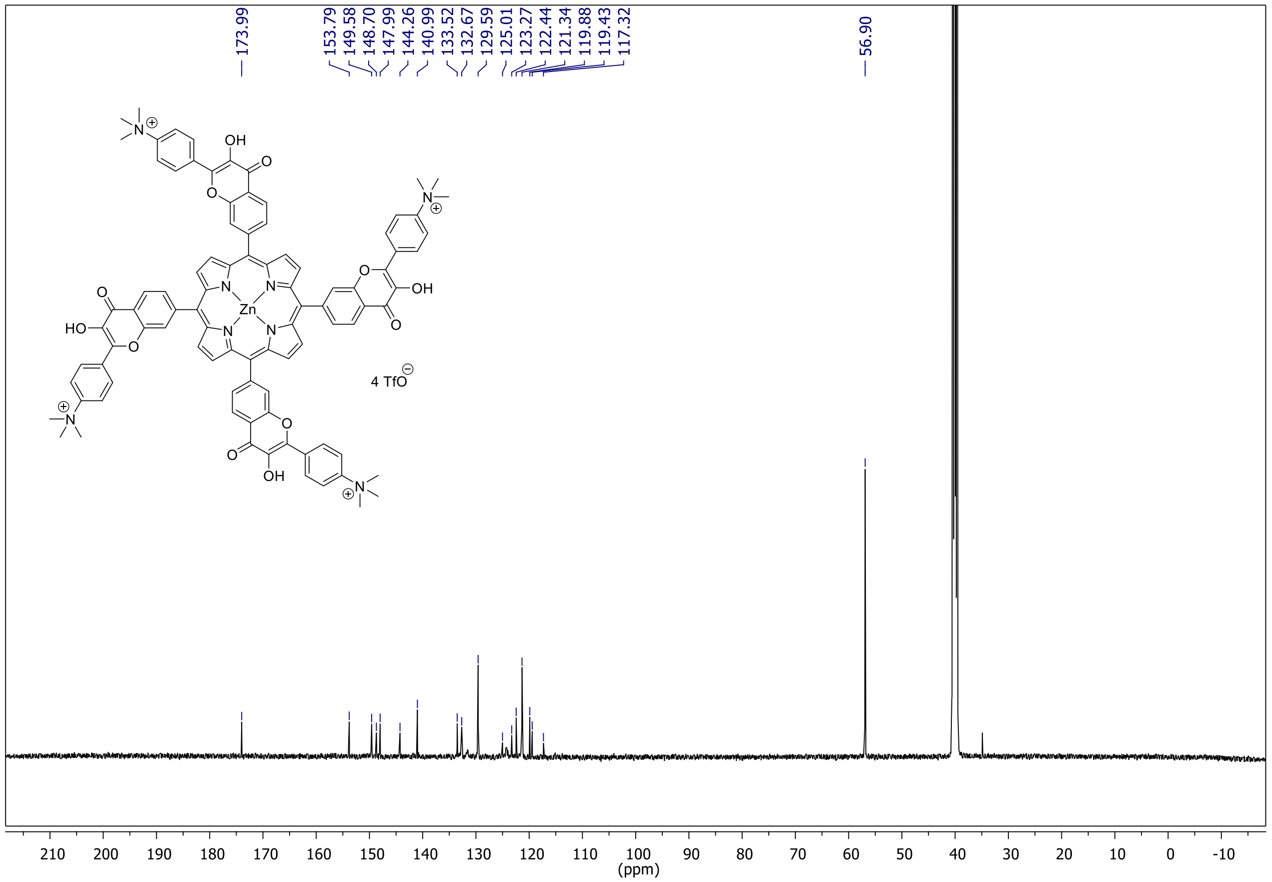

Figure S40. ^{13}C NMR (125 MHz, d_6-DMSO): **2-Zn**.

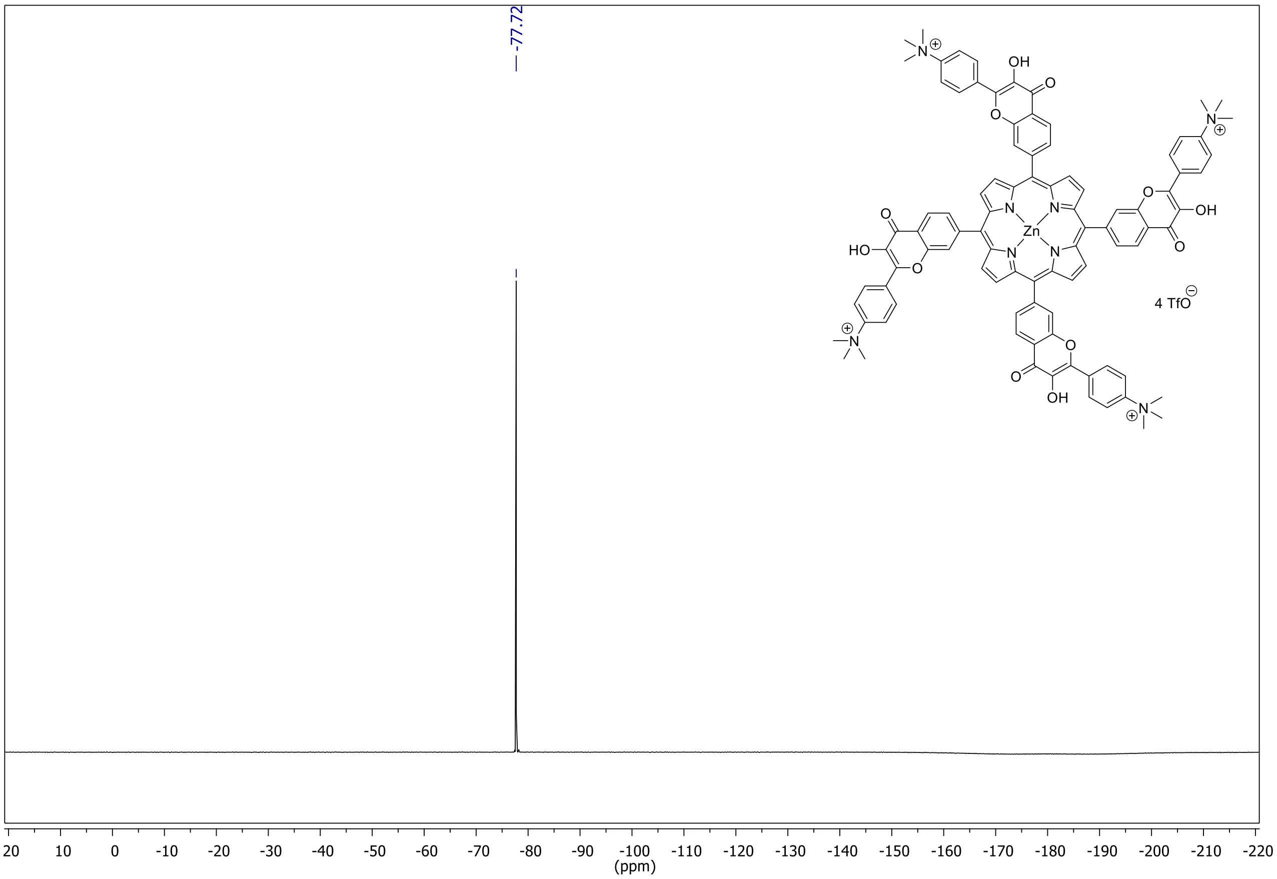

Figure S41. ^{19}F NMR (476 MHz, d_6-DMSO): **2-Zn**.

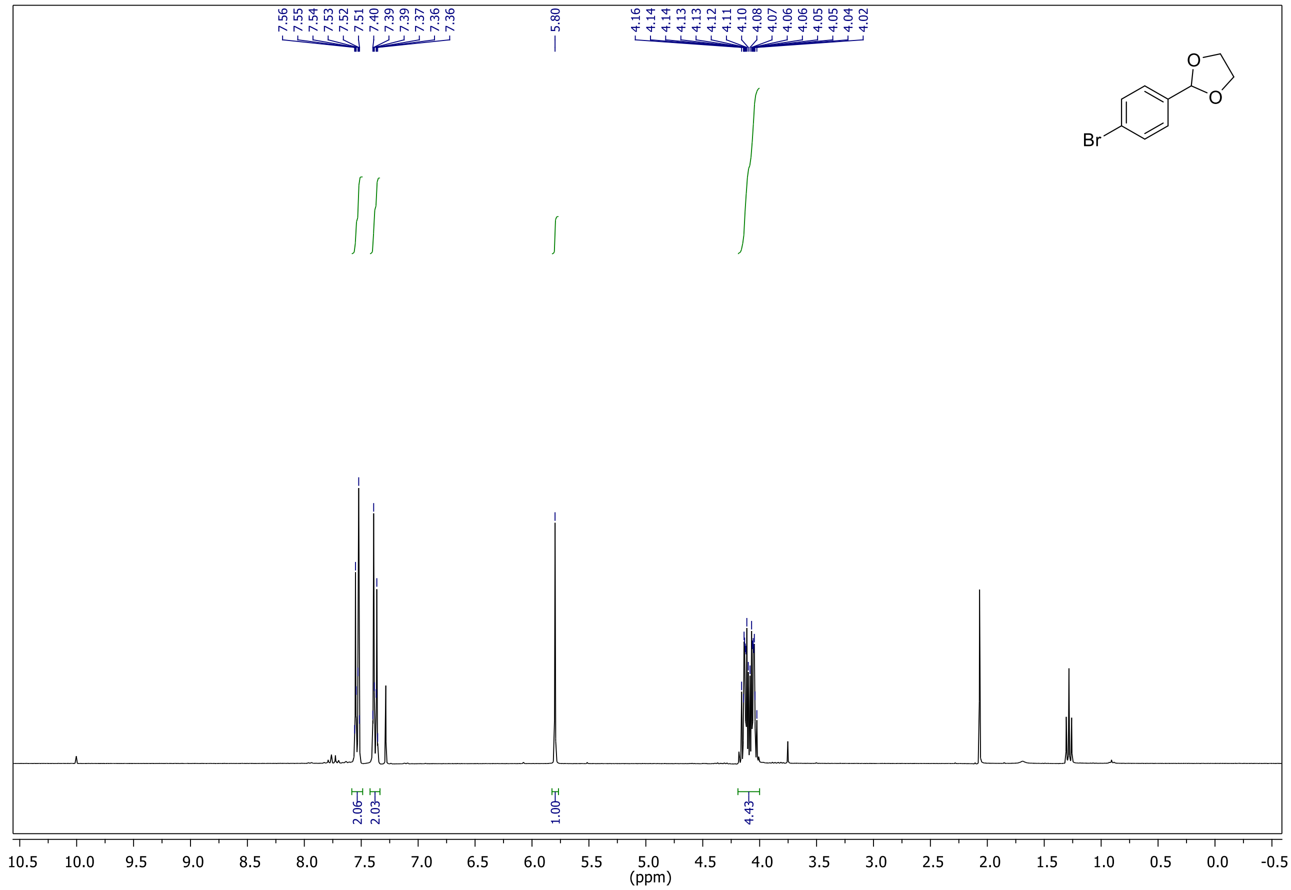

Figure S42. ^{1}H NMR (300 MHz, $CDCl_3$): **14**.

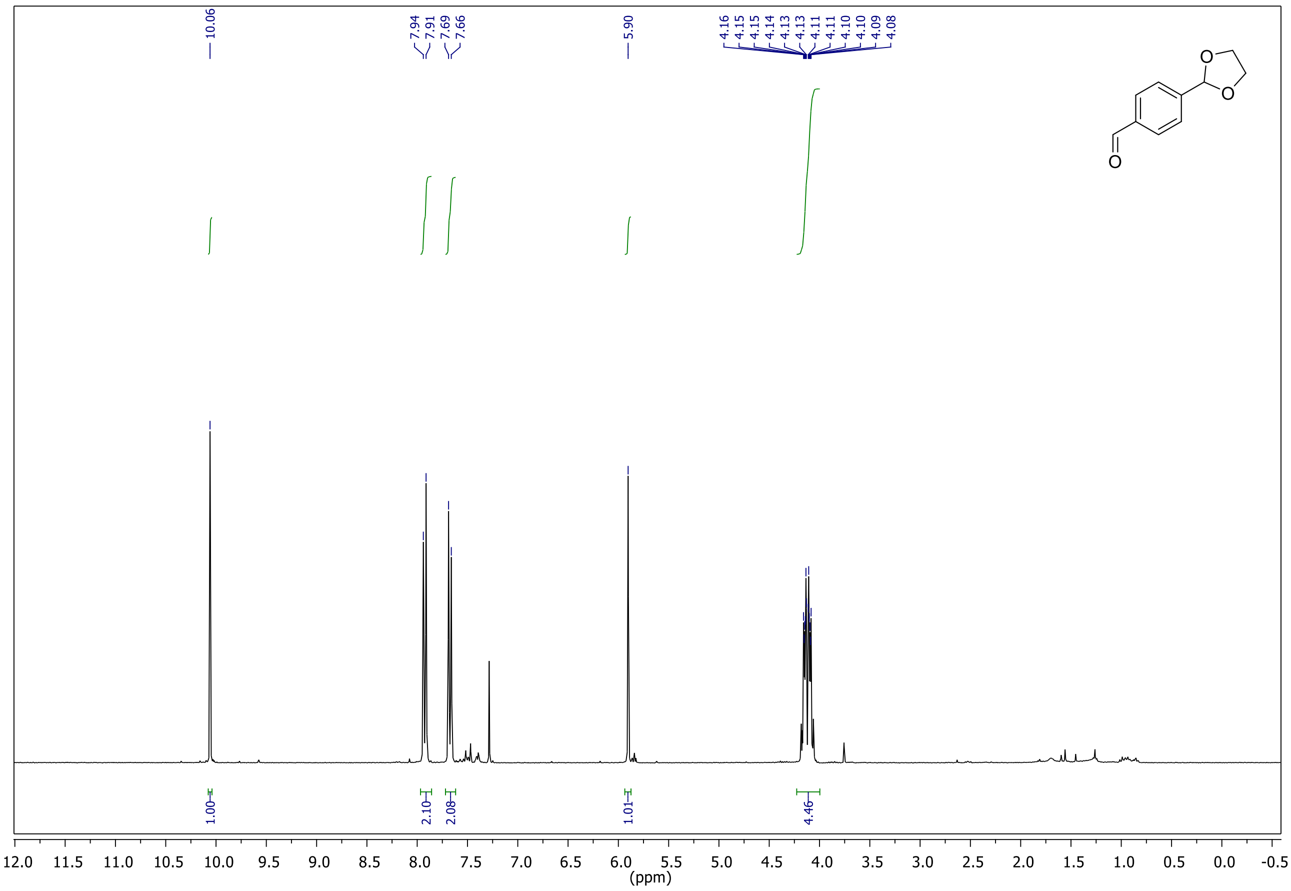

Figure S43. ^{1}H NMR (300 MHz, $CDCl_3$): **15**.

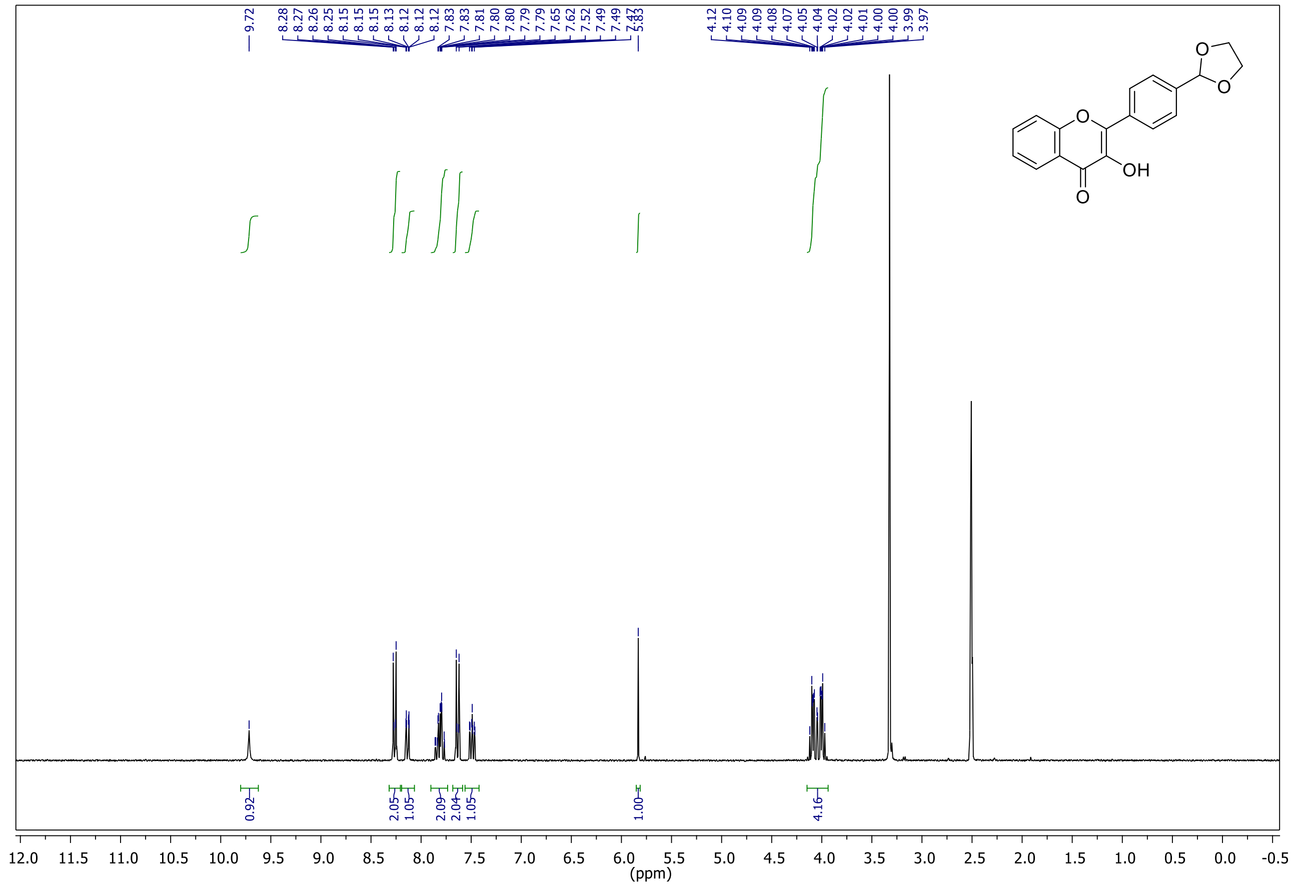

Figure S44. ^{1}H NMR (300 MHz, d_6-DMSO): **16**.

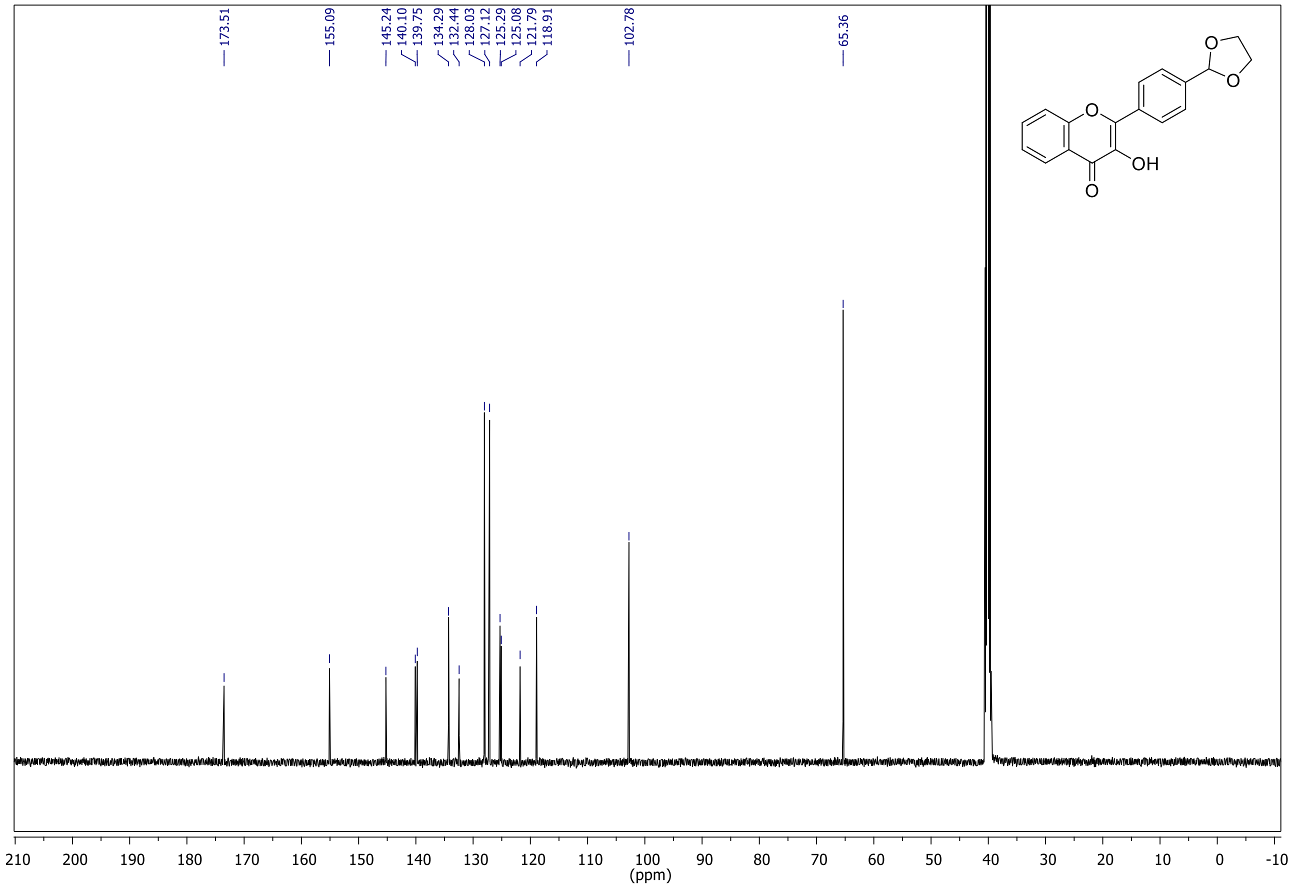

Figure S45. ^{13}C NMR (125 MHz, d_6-DMSO): **16**.

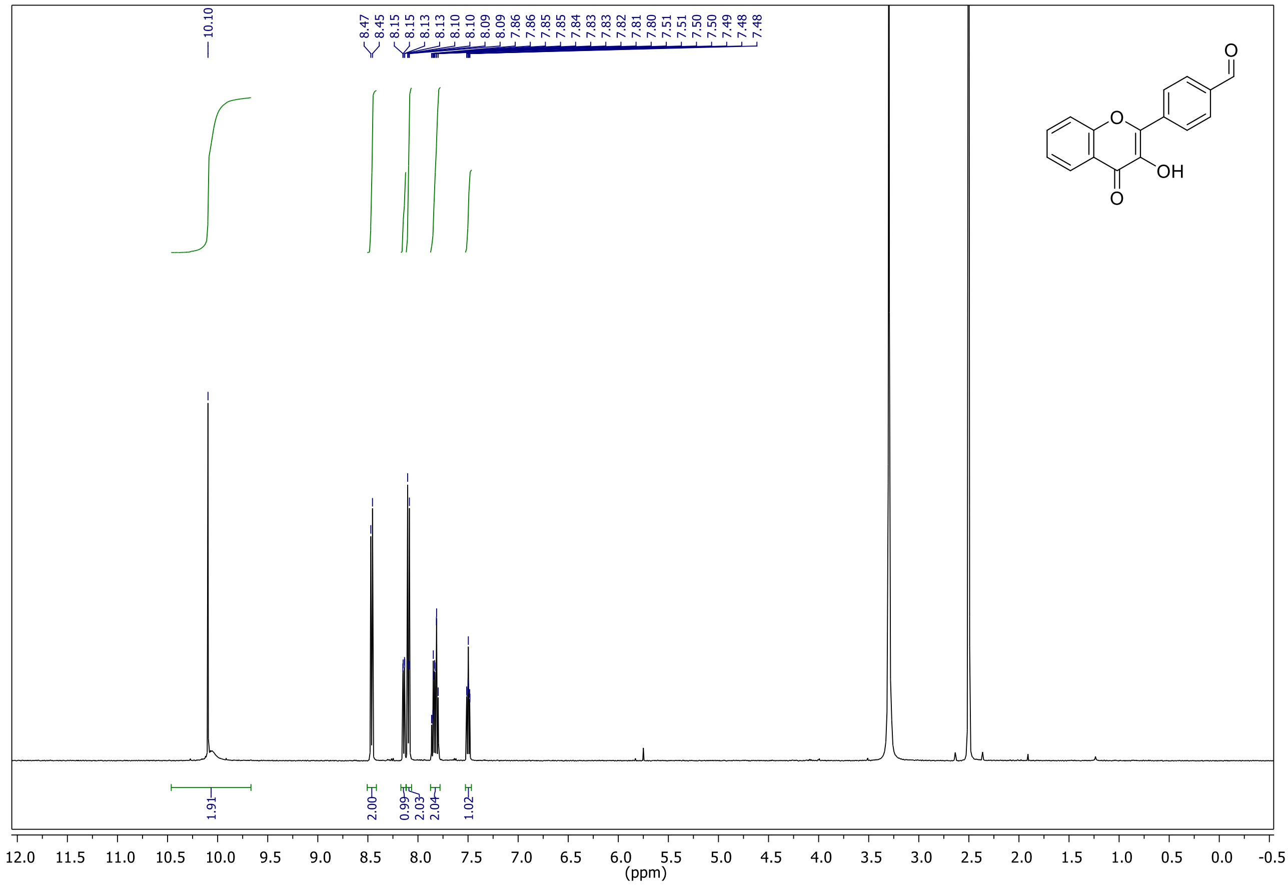

Figure S46. ^{1}H NMR (500 MHz, d_6-DMSO): **17**.

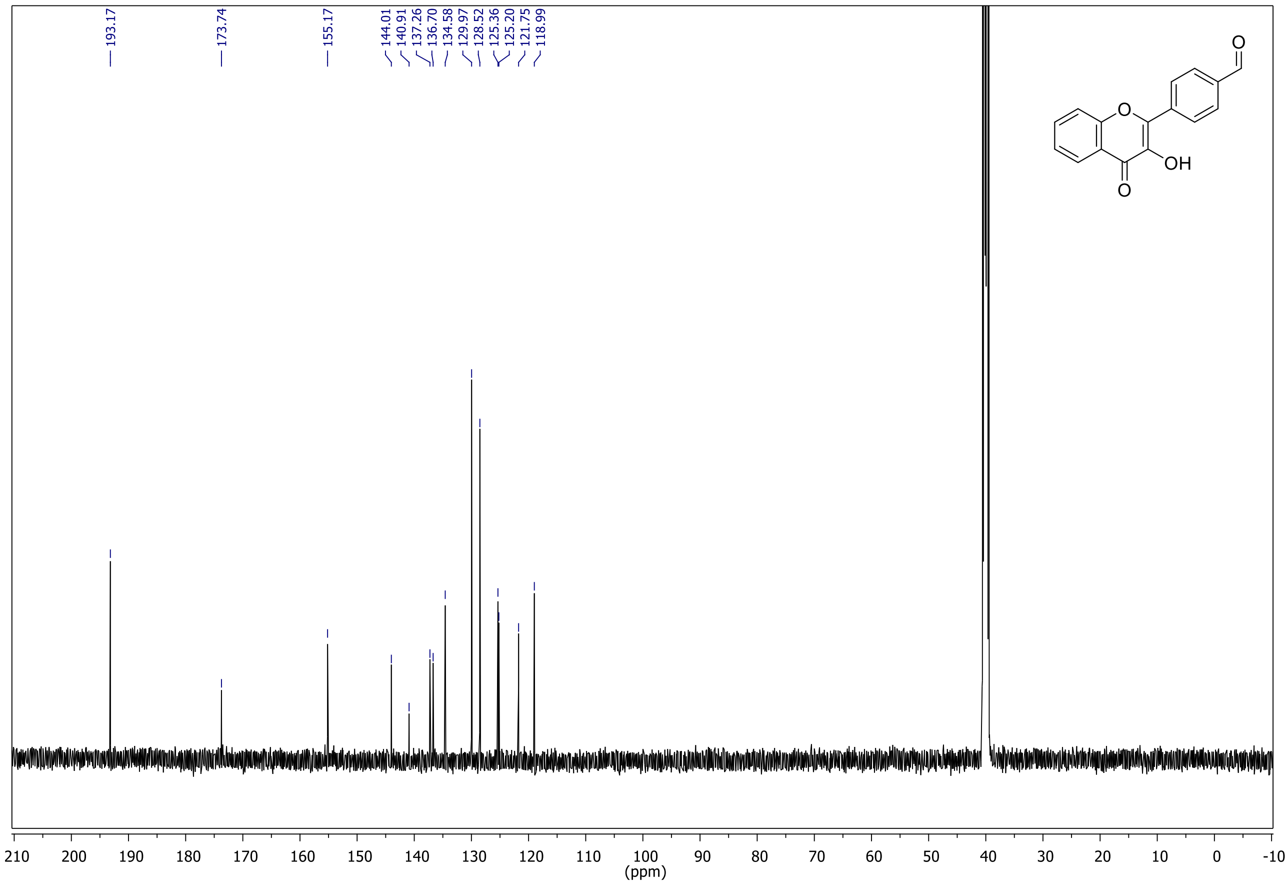

Figure S47. ^{13}C NMR (125 MHz, d_6-DMSO): **17**.

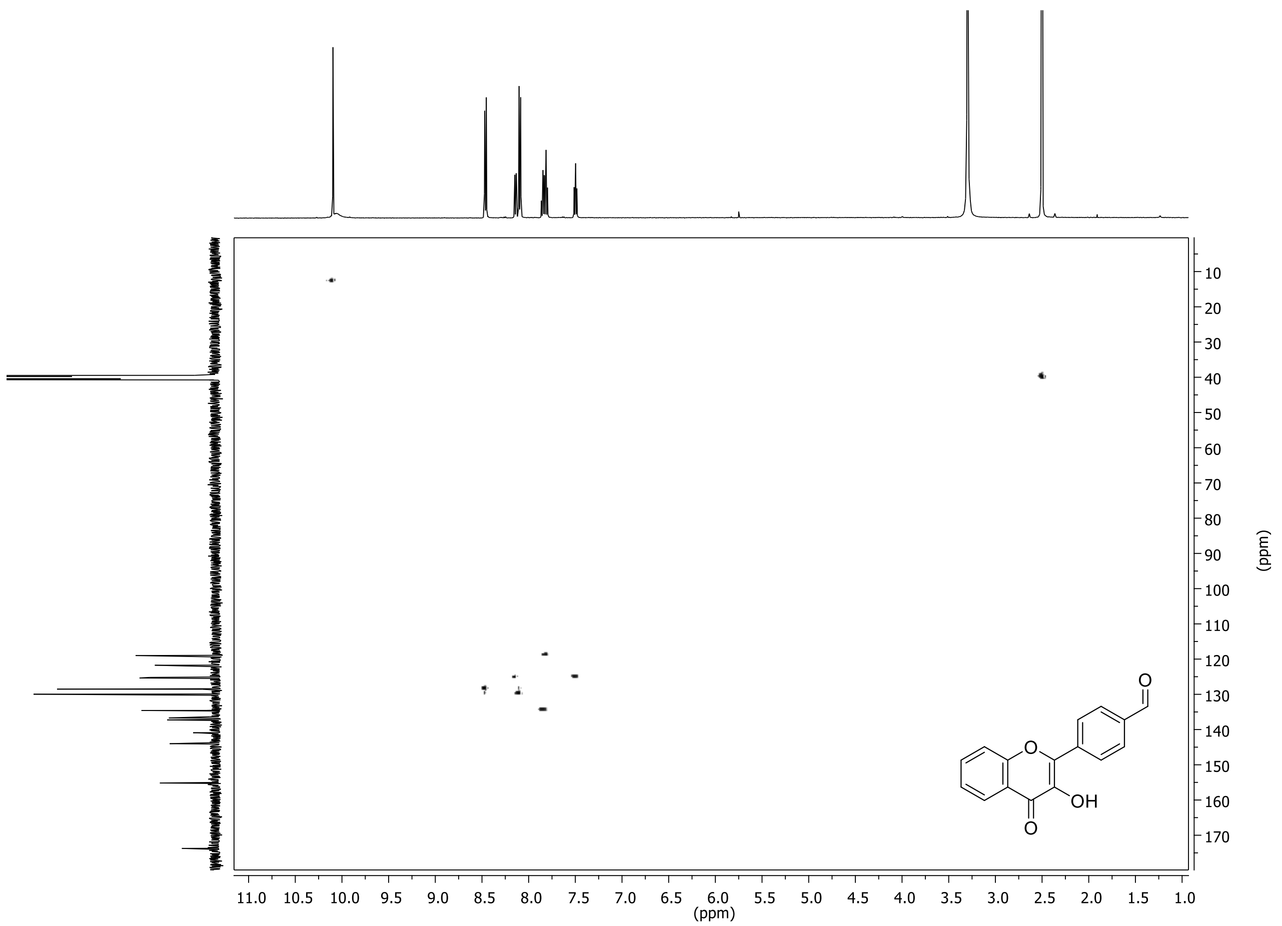

Figure S48. ^{1}H–^{13}C HSQC (500 MHz, d_6–DMSO): **17**.

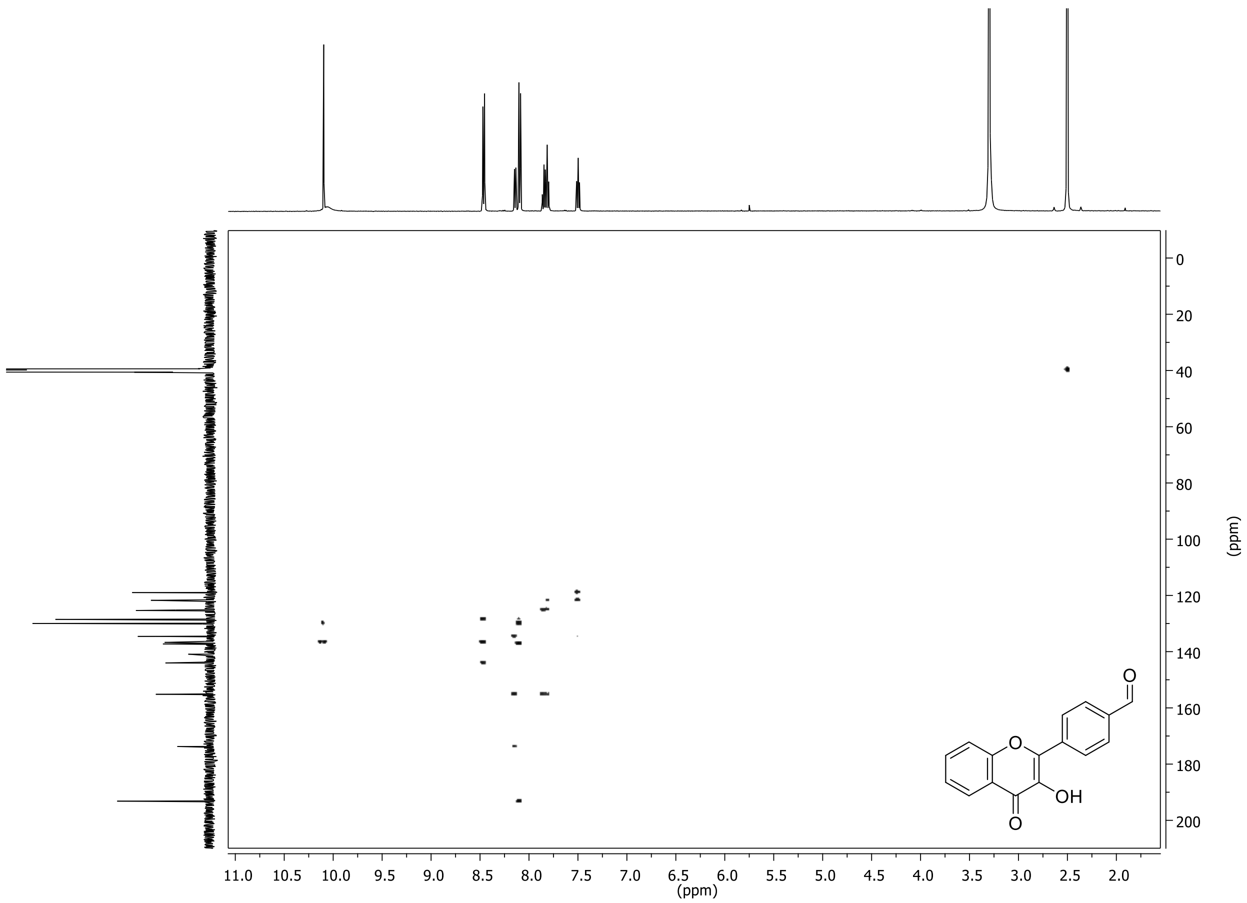

Figure S49. ^{1}H–^{13}C HMBC (500 MHz, d_6–DMSO): **17**.

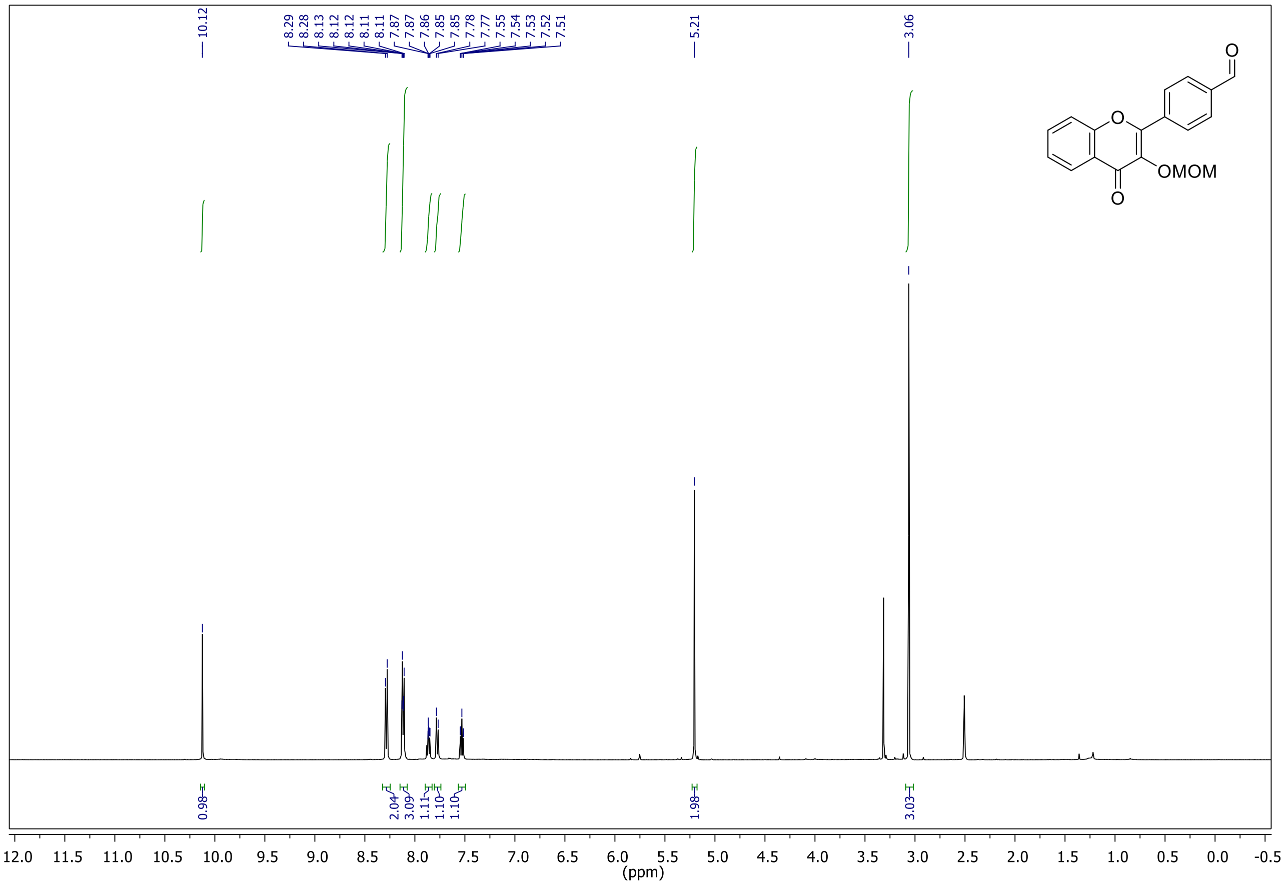

Figure S50. ^{1}H NMR (500 MHz, d_6-DMSO): **18**.

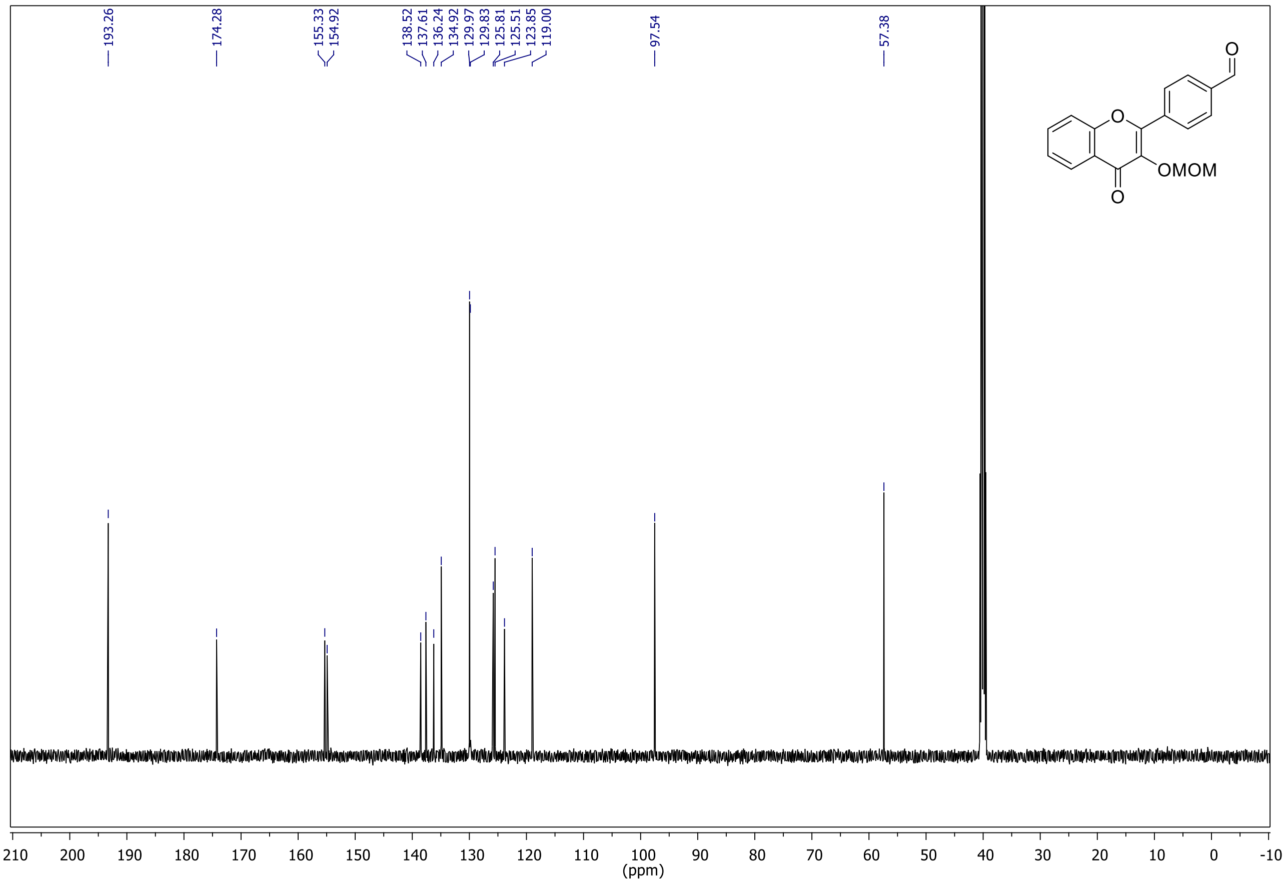

Figure S51. ^{13}C NMR (125 MHz, d_6-DMSO): **18**.

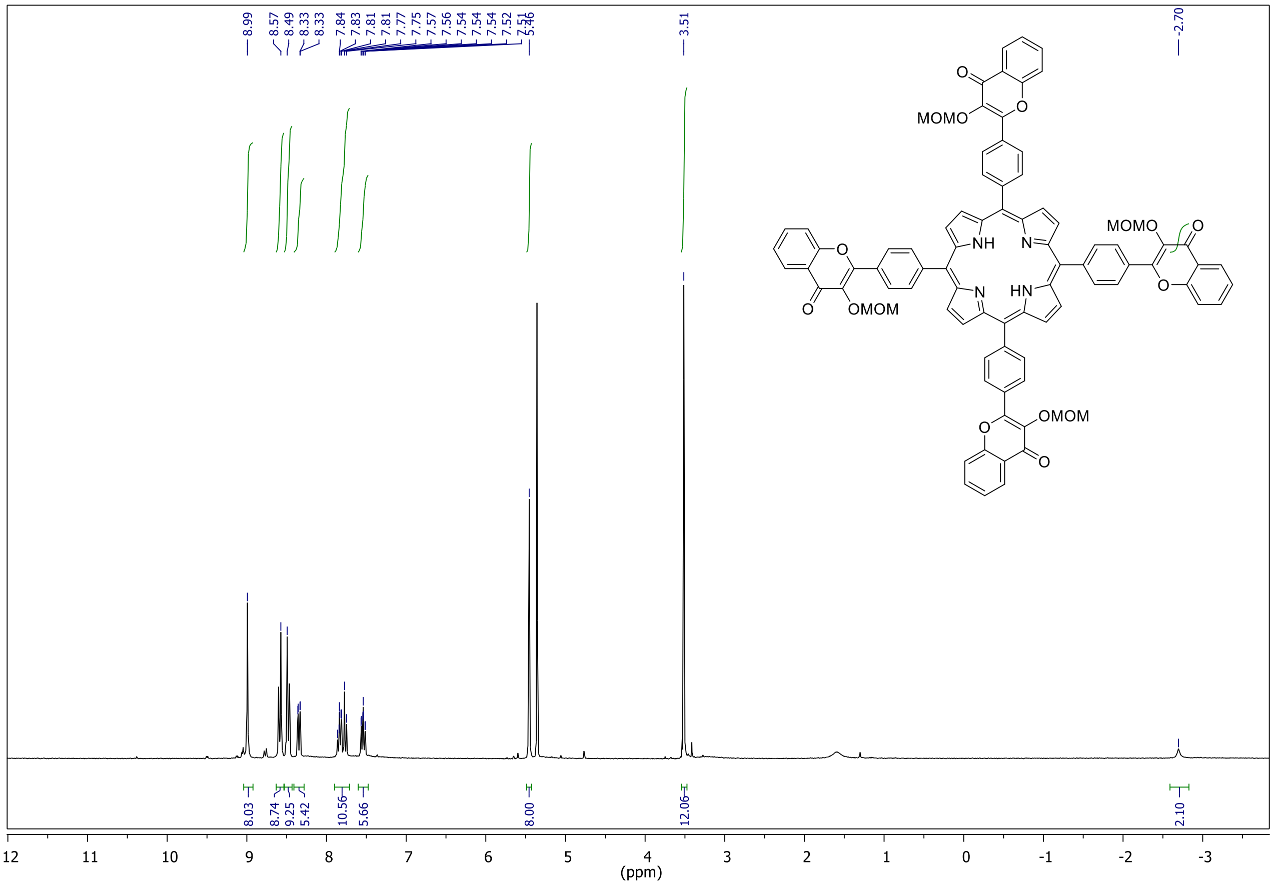

Figure S52. ^{1}H NMR (500 MHz, CD_2Cl_2): **19**.

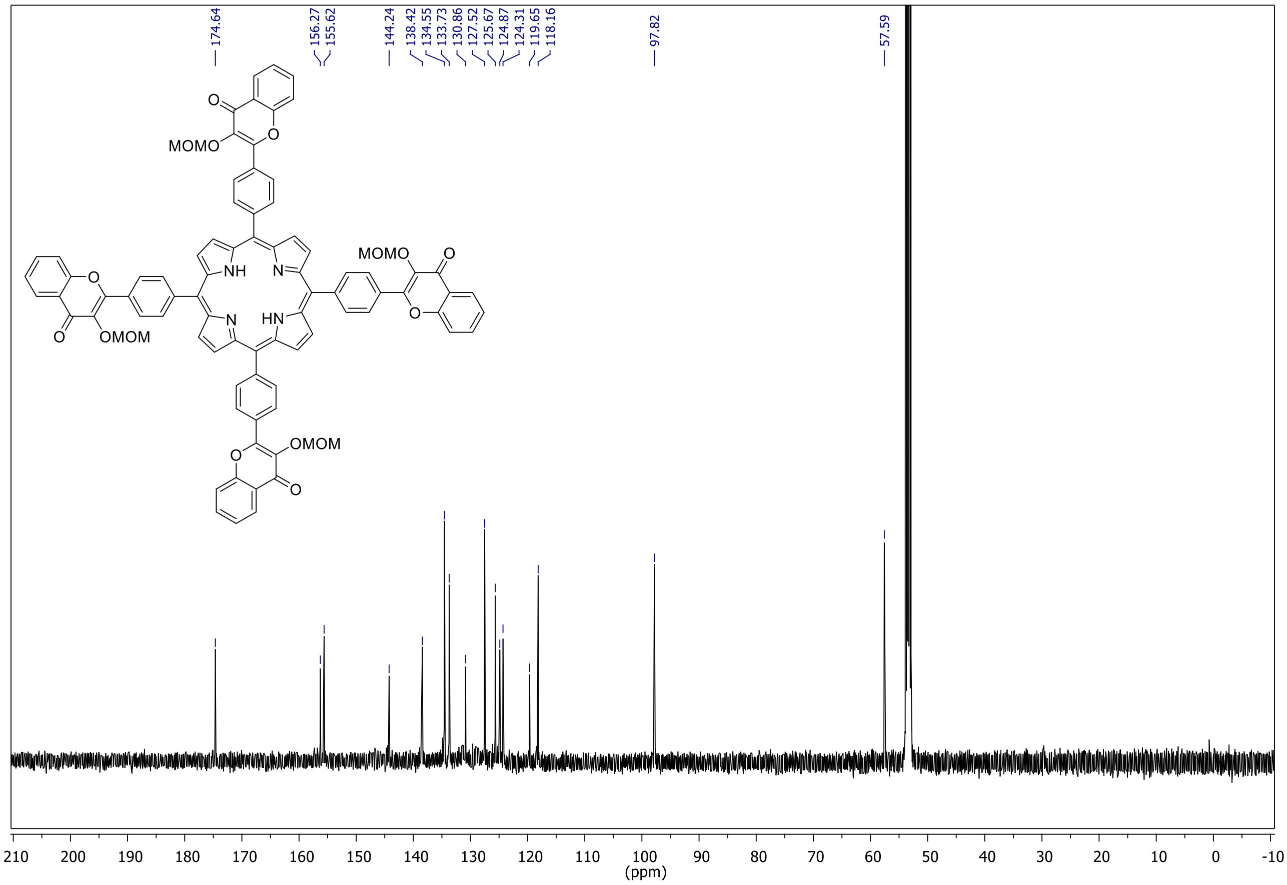

Figure S53. ^{13}C NMR (125 MHz, CD_2Cl_2): **19**.

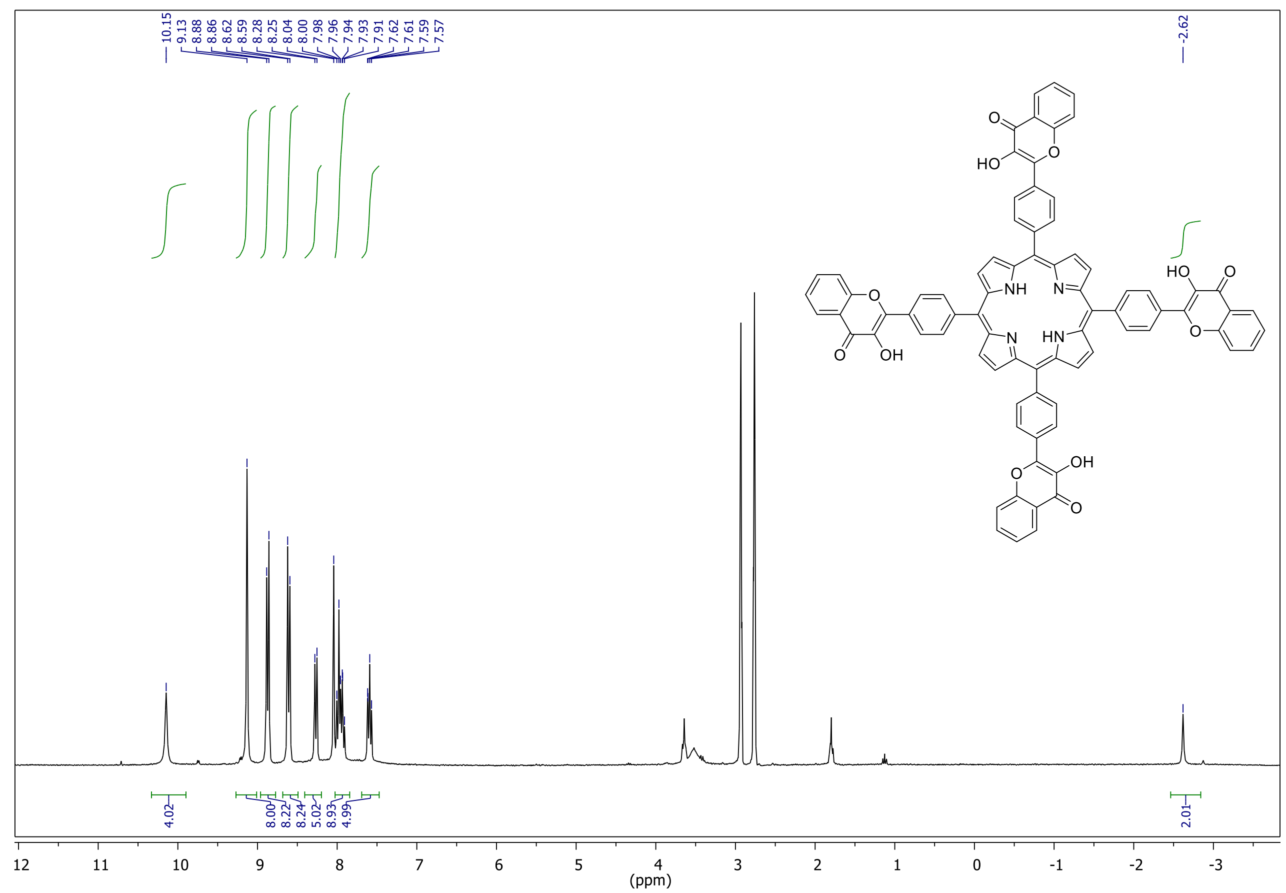

Figure S54. ^{1}H NMR (300 MHz, d_7-DMF): **3**.

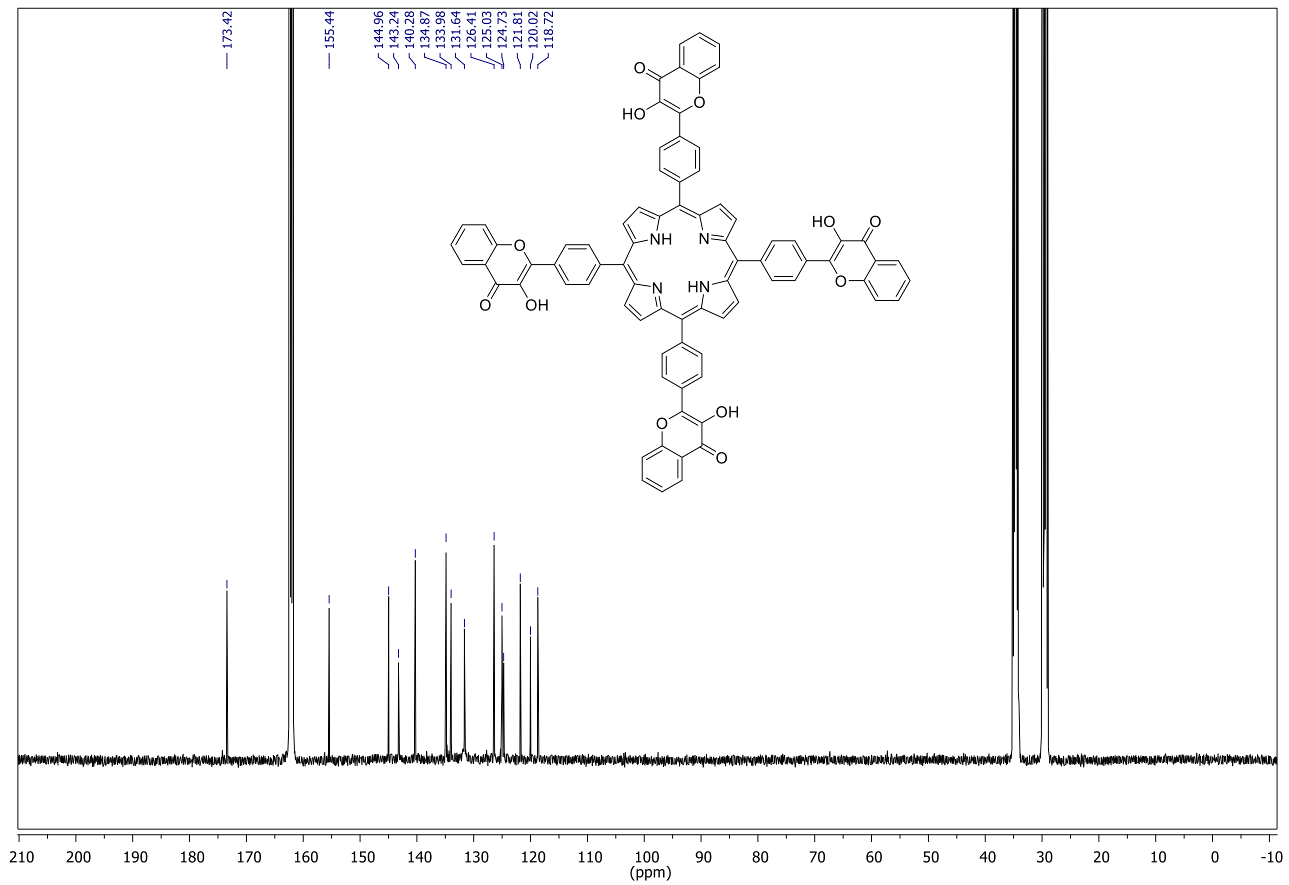

Figure S55. ^{13}C NMR (125 MHz, d_7-DMF): **3**.

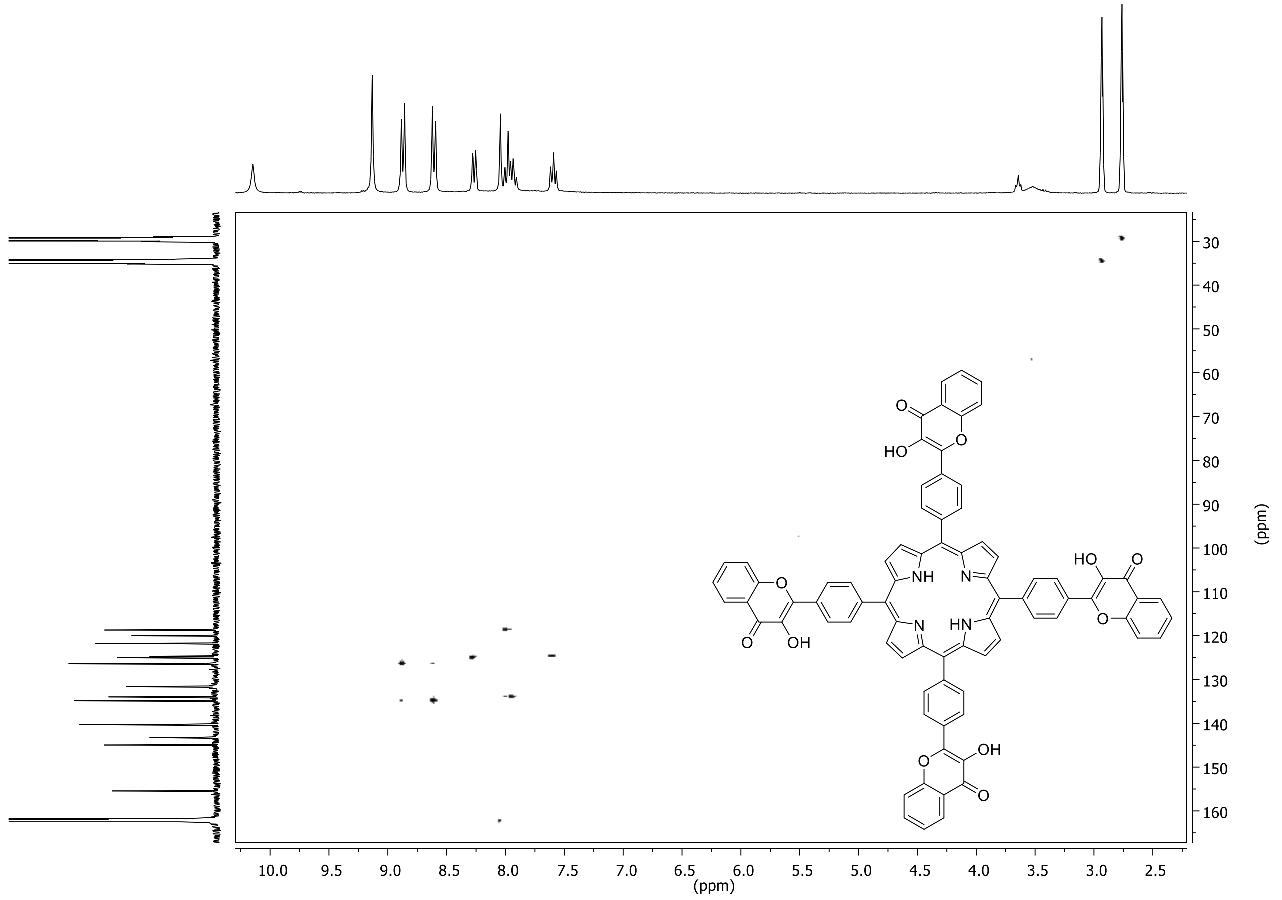

Figure S56. ^{1}H−^{13}C HSQC (500 MHz, d_7-DMF): **3**.

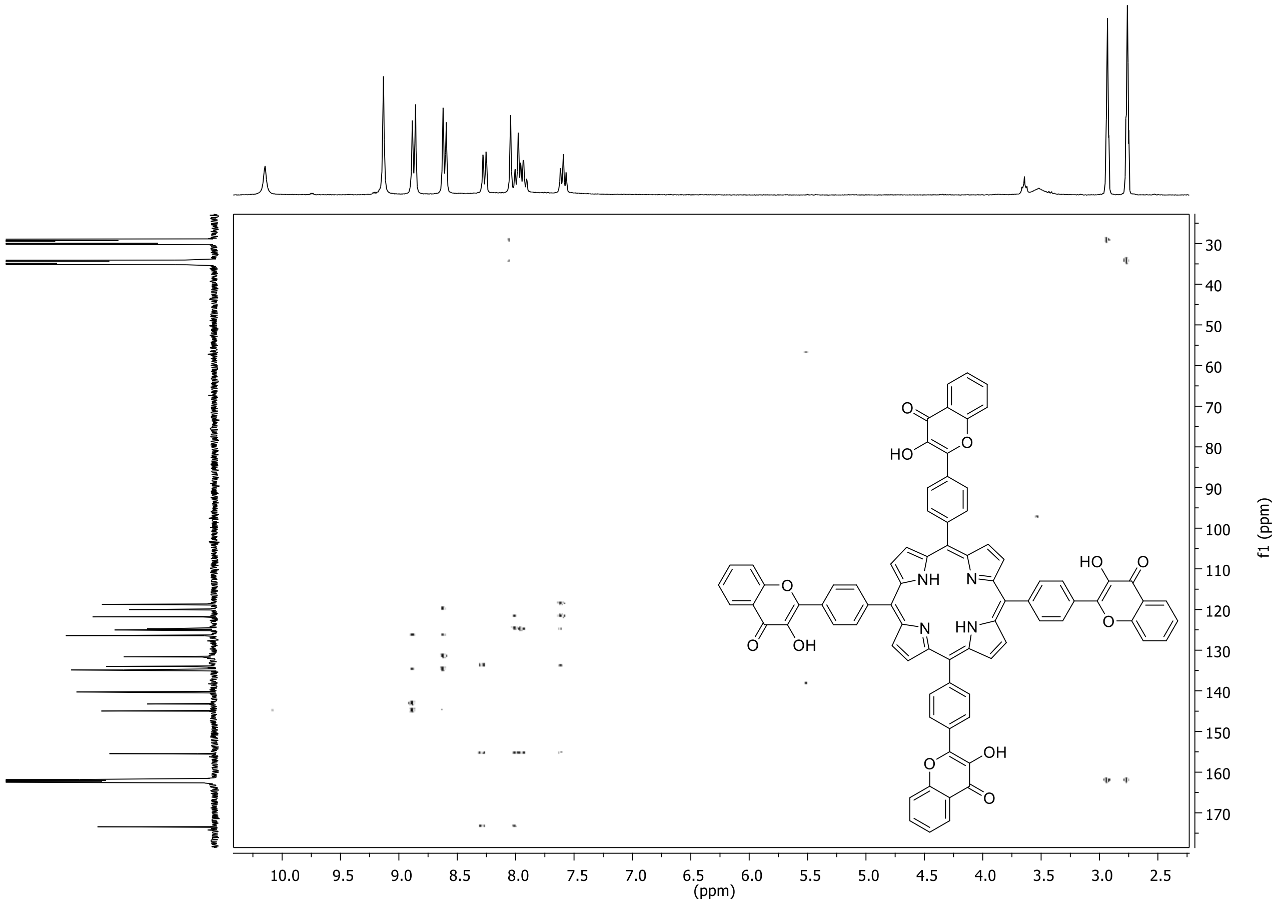

Figure S57. ^{1}H−^{13}C HMBC (500 MHz, d_7-DMF): **3**.

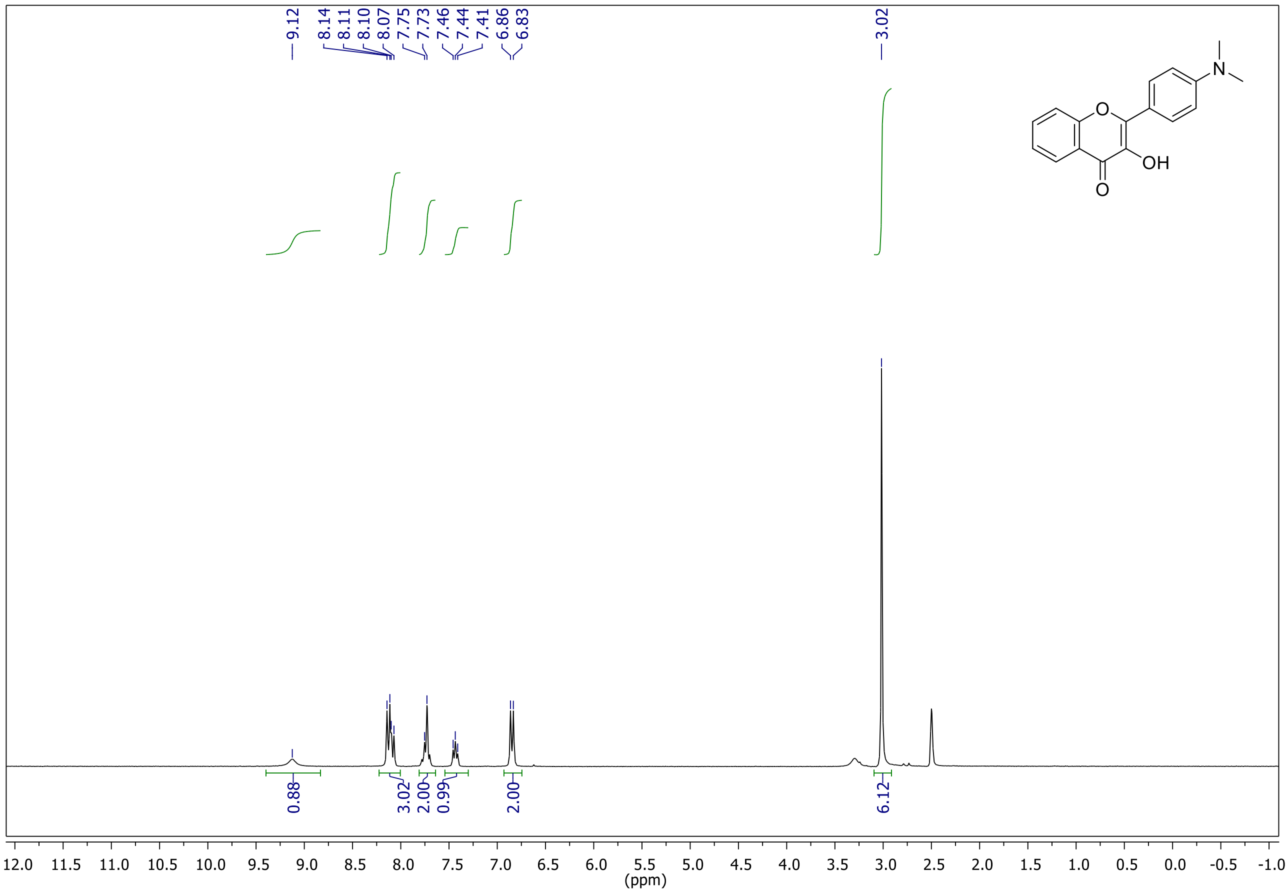

Figure S58. ^{1}H NMR (300 MHz, d_6-DMSO): **20**.

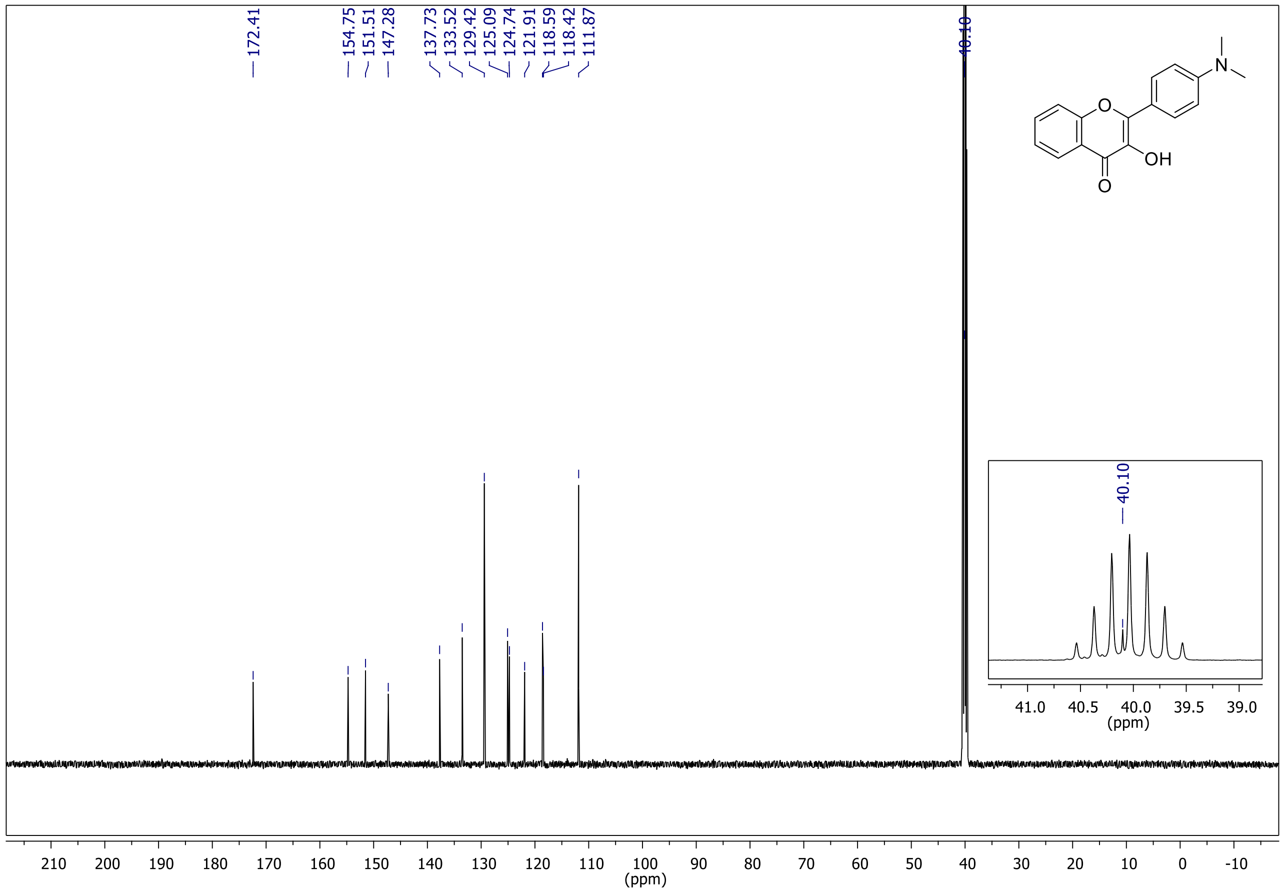

Figure S59. ^{13}C NMR (125 MHz, d_6-DMSO): **20**.

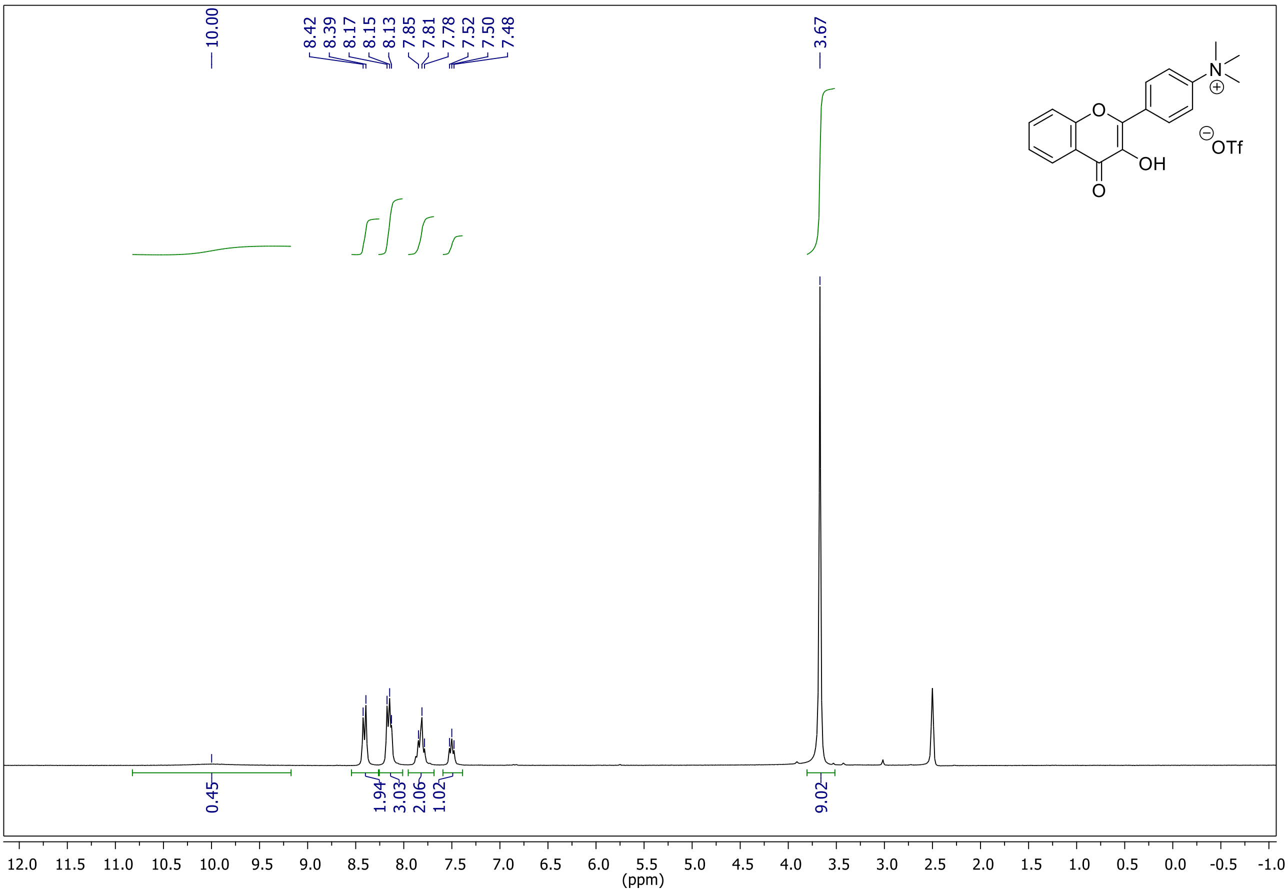

Figure S60. ^{1}H NMR (300 MHz, d_6-DMSO): **10**.

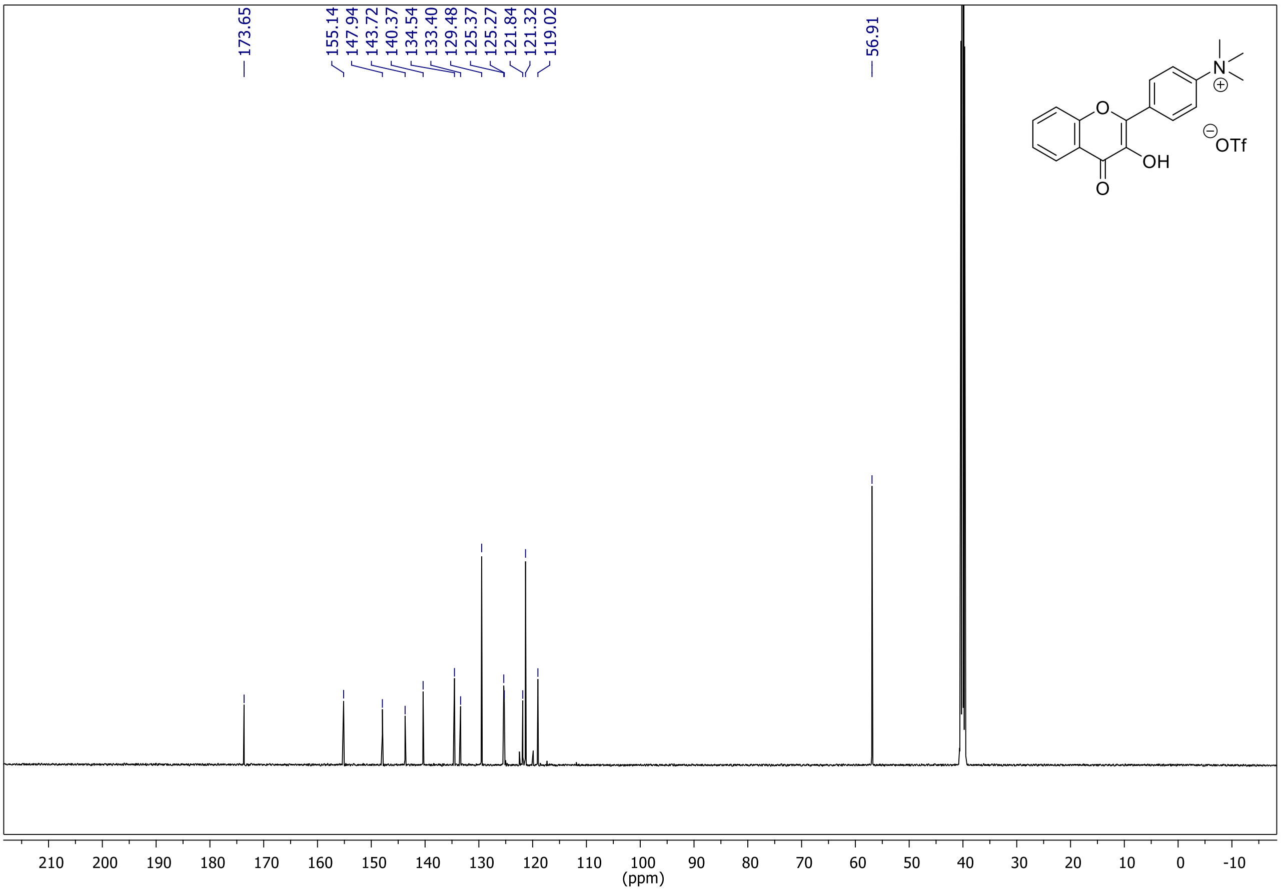

Figure S61. ^{13}C NMR (125 MHz, d_6-DMSO): **10**.

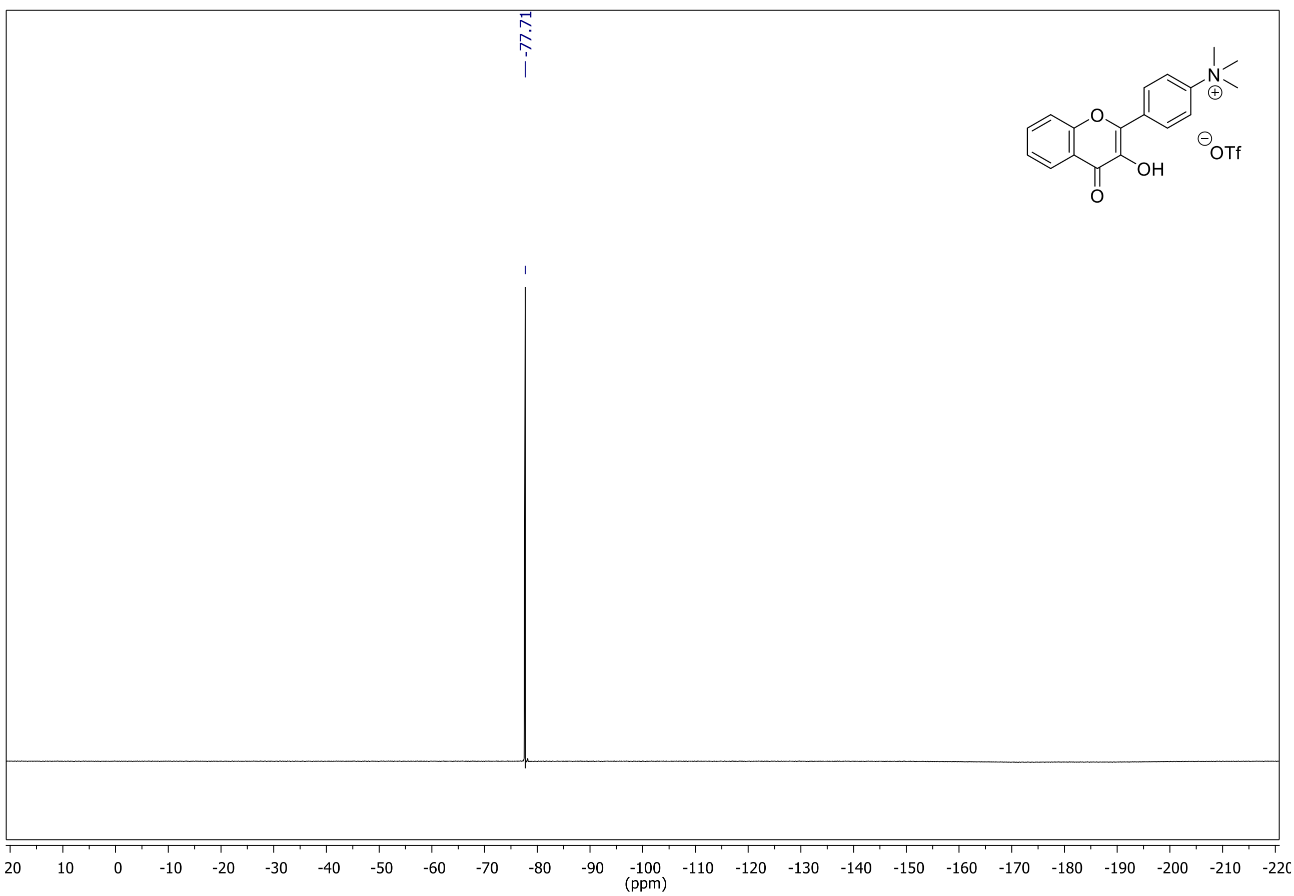

Figure S62. ^{19}F NMR (476 MHz, d_6-DMSO): **10**.

HRMS spectra

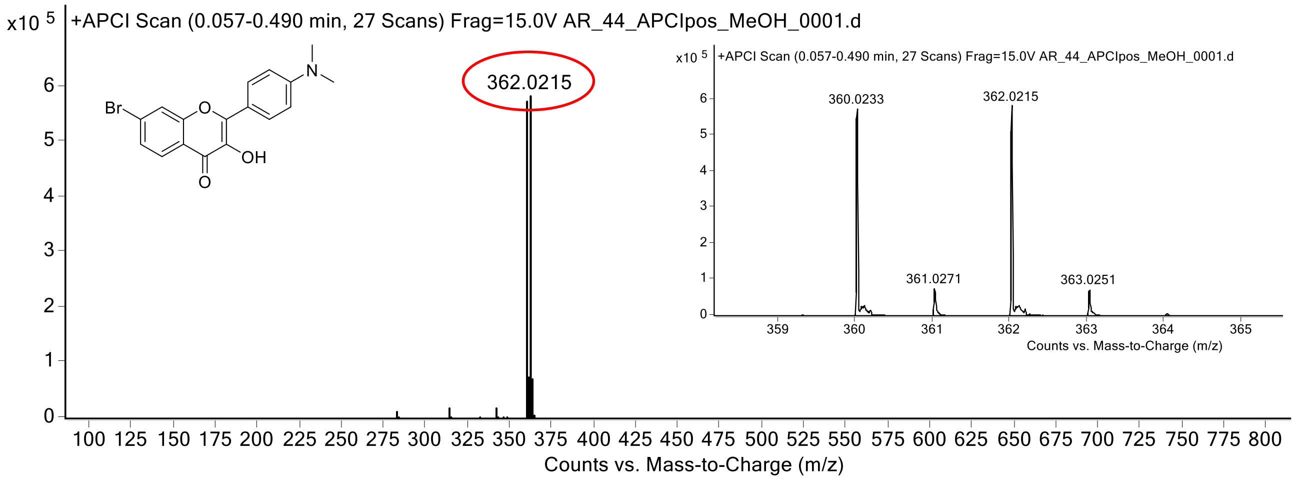

Figure S63. HRMS (APCI+): **6** (calcd. for $C_{17}H_{15}BrNO_3^+$ [M + H$^+$] 360.0230, found 360.0233).

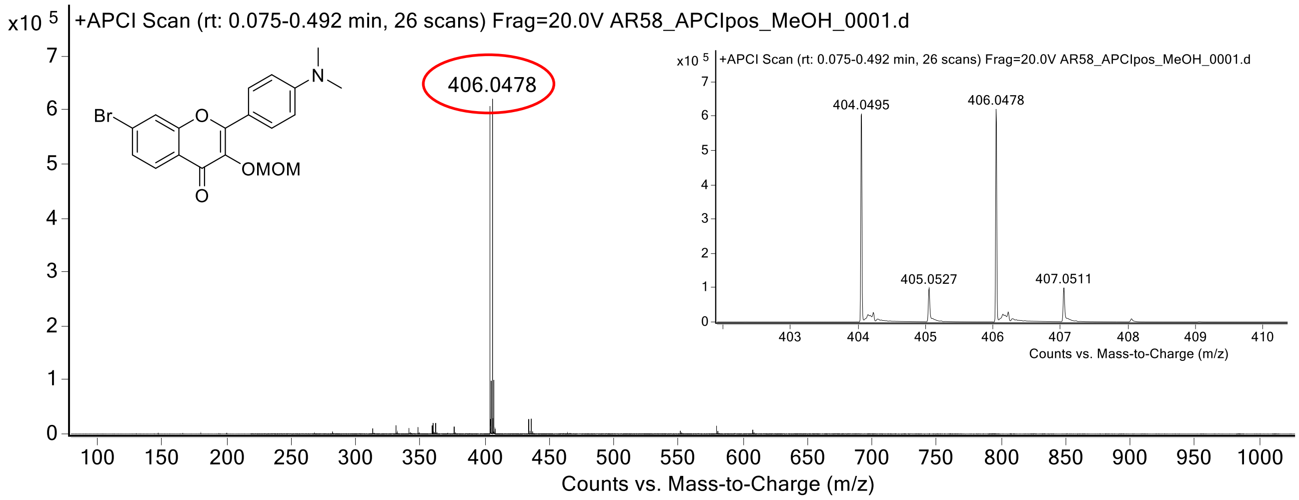

Figure S64. HRMS (APCI+): **12** (calcd. for $C_{19}H_{19}BrNO_4^+$ [M + H$^+$] 404.0497, found 404.0495).

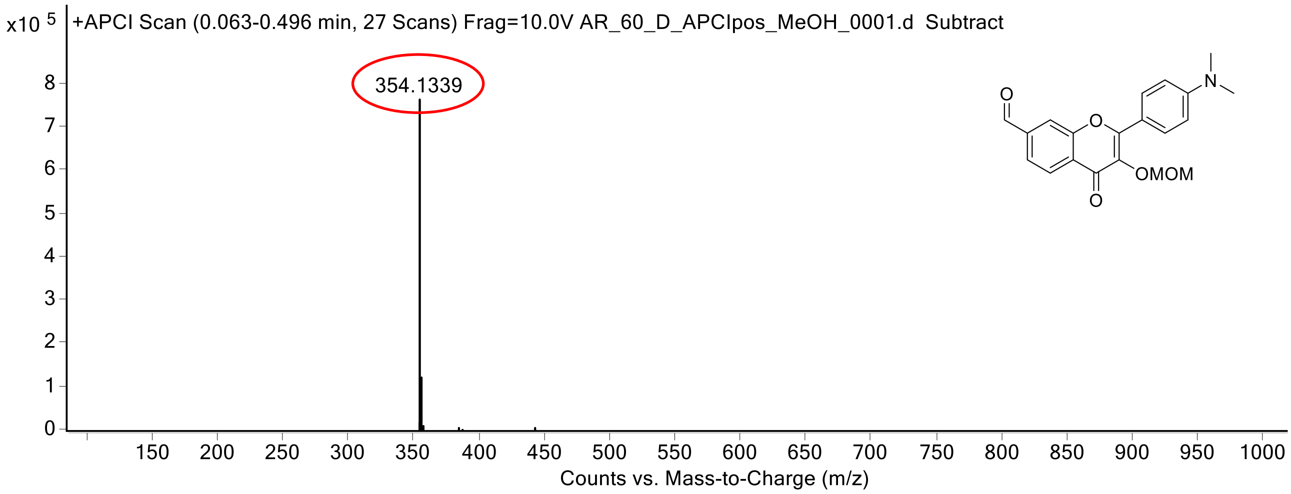

Figure S65. HRMS (APCI+): **7** (calcd. for $C_{20}H_{20}NO_5^+$ [M + H$^+$] 354.1336, found 354.1339).

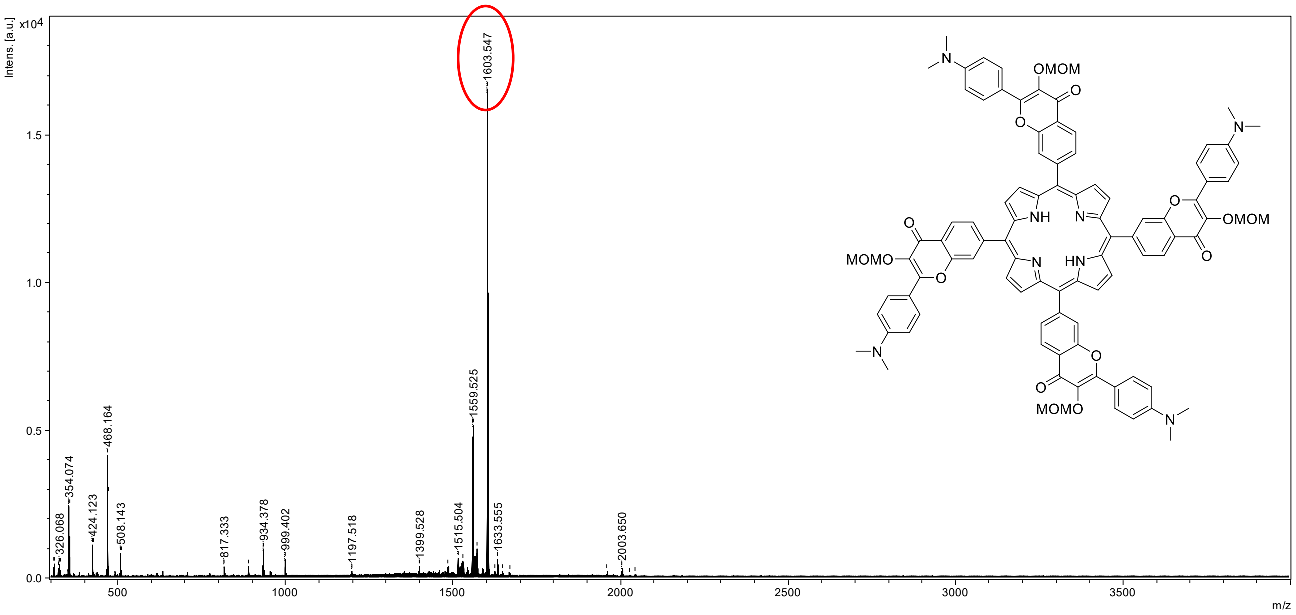

Figure S66. HRMS (MALDI+): **8** (calcd. for $C_{96}H_{83}N_8O_{16}^+$ [M + H$^+$] 1603.588, found 1603.547).

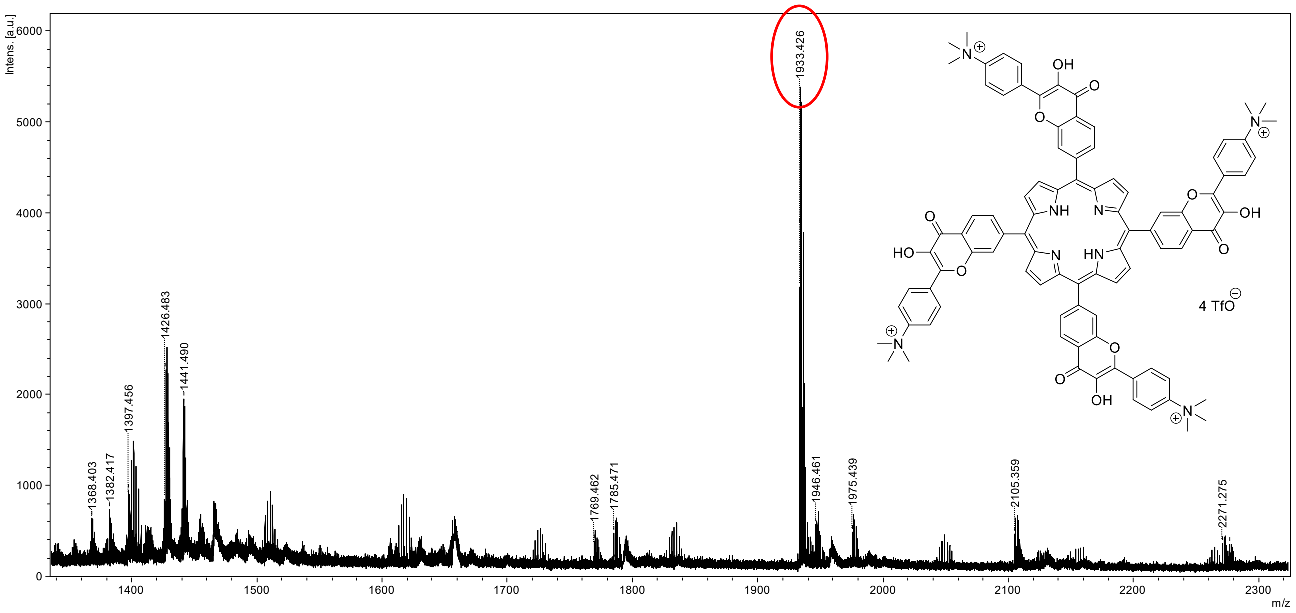

Figure S67. HRMS (MALDI+): **2** (calcd. for $C_{95}H_{78}F_9N_8O_{21}S_3^+$ [M – TfO$^-$] 1933.429, found 1933.426).

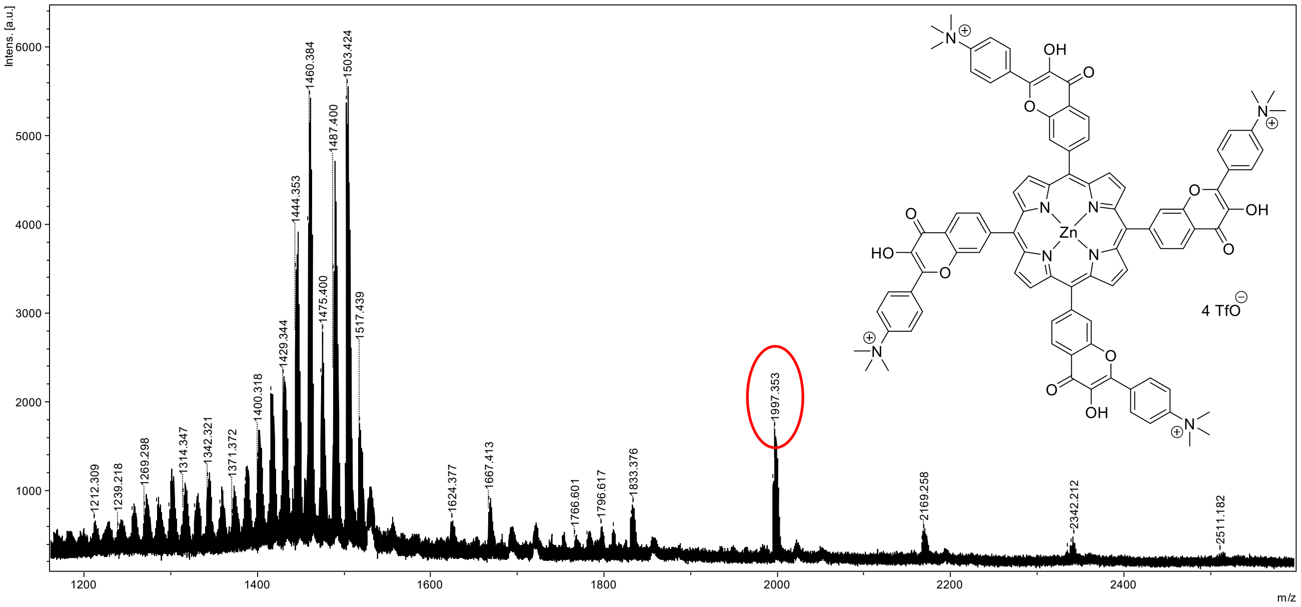

Figure S68. HRMS (MALDI+): **2-Zn** (calcd. for $C_{95}H_{76}F_9N_8O_{21}S_3Zn^+$ [M – TfO^-] 1995.343, found 1995.348).

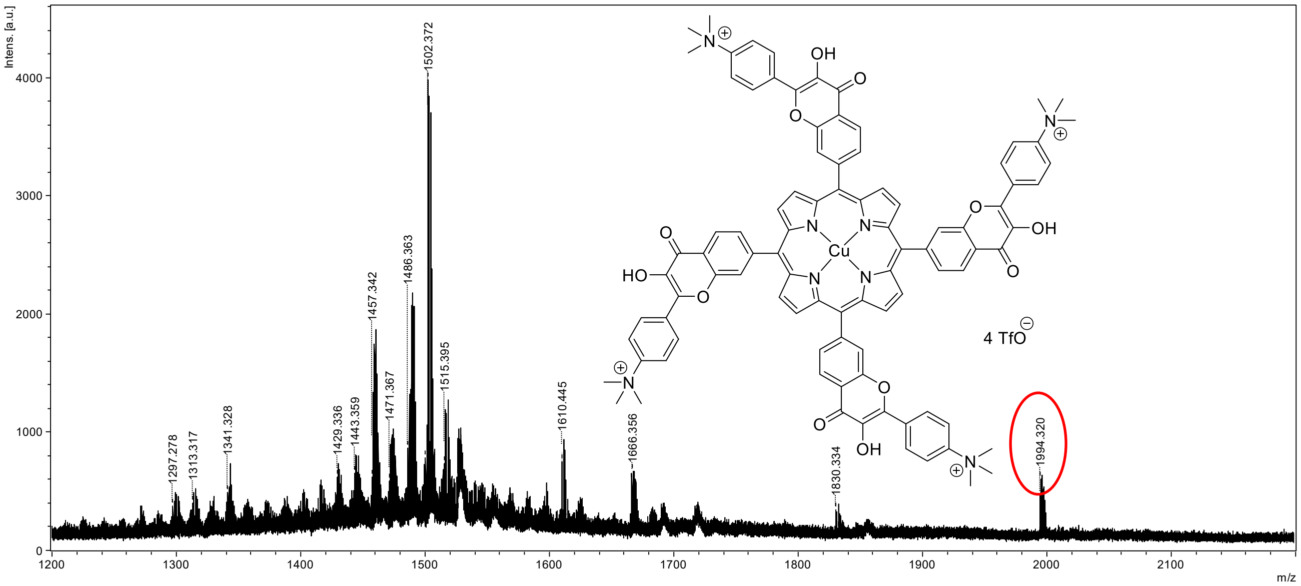

Figure S69. HRMS (MALDI+): **2-Cu** (calcd. for $C_{95}H_{76}CuF_9N_8O_{21}S_3^+$ [M – TfO^-] 1994.343, found 1994.32).

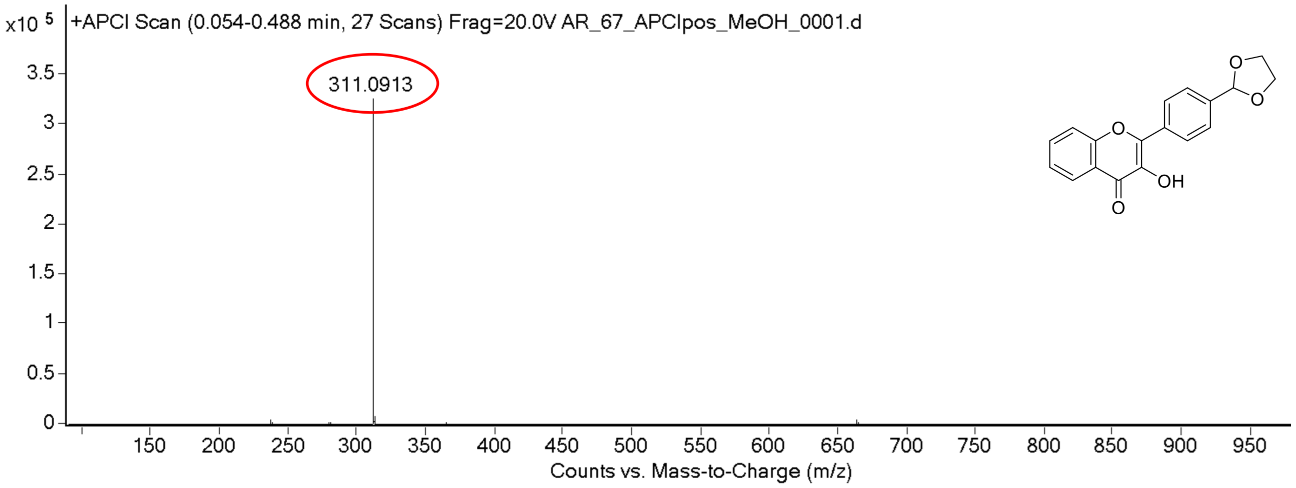

Figure S70. HRMS (APCI+): **16** (calcd. for $C_{18}H_{15}O_5^+$ [M + H$^+$] 311.0914, found 311.0913).

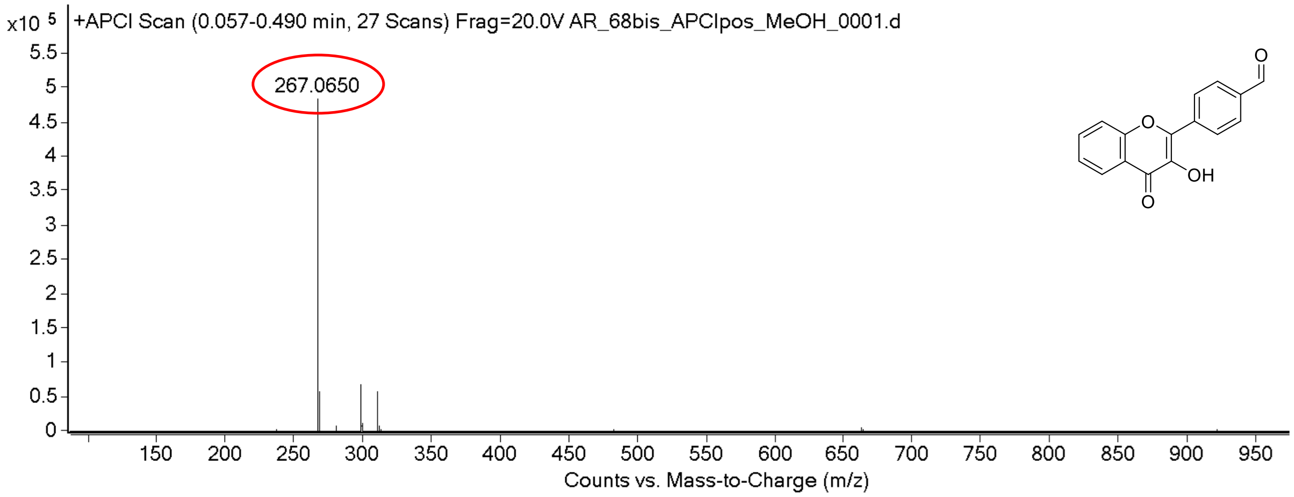

Figure S71. HRMS (APCI+): **17** (calcd. for $C_{16}H_{11}O_4^+$ [M + H$^+$] 267.0652, found 267.0650).

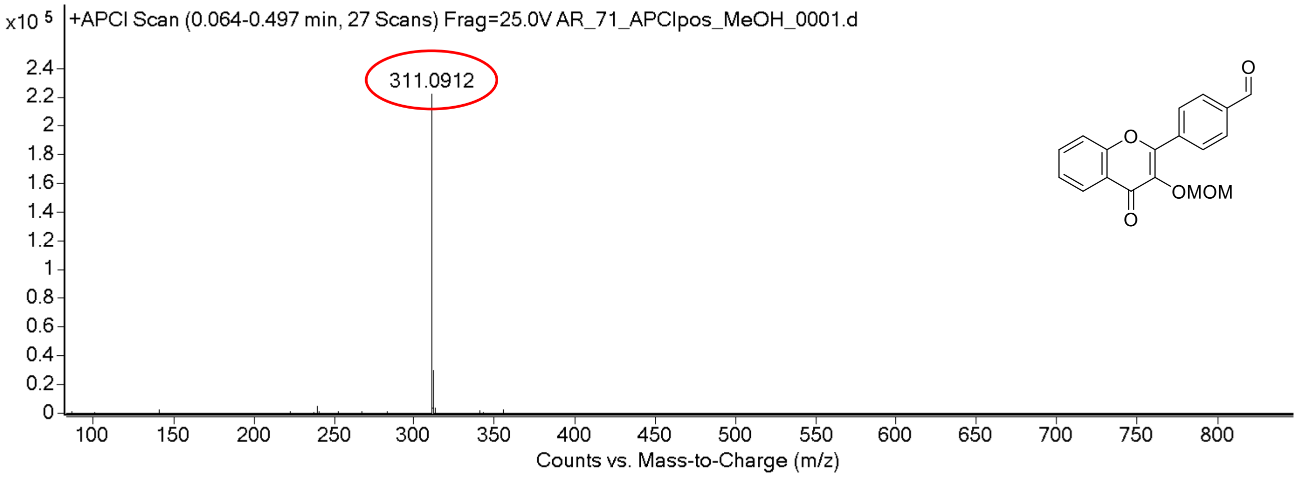

Figure S72. HRMS (APCI+): **18** (calcd. for $C_{18}H_{15}O_5^+$ [M + H$^+$] 311.0914, found 311.0912).

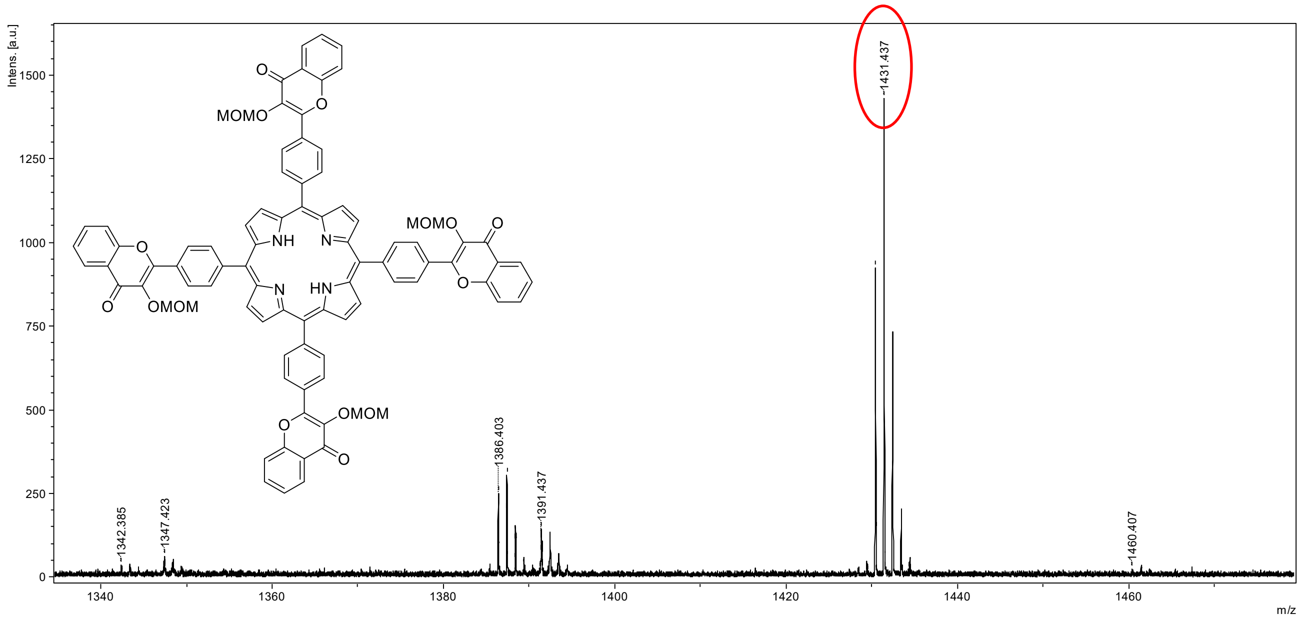

Figure S73. HRMS (MALDI–): **19** (calcd. for $C_{88}H_{61}N_4O_{16}^-$ [M – H+] 1429.408, found 1429.411).

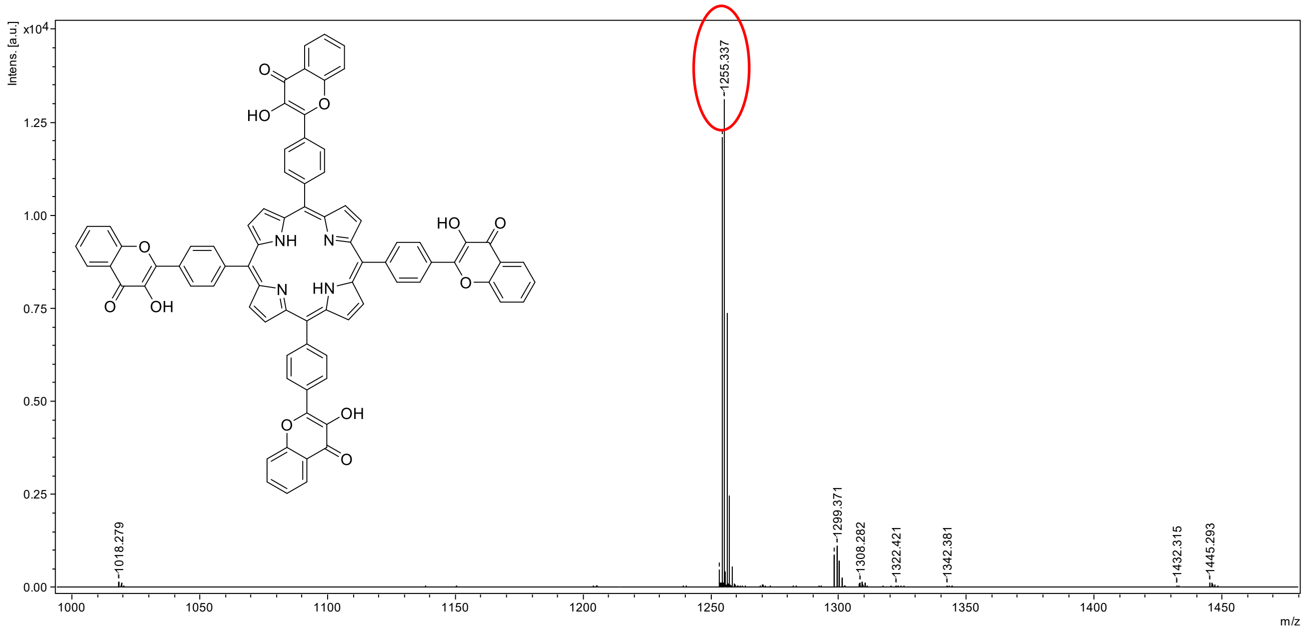

Figure S74. HRMS (MALDI–): **3** (calcd. for $C_{80}H_{45}N_4O_{12}^-$ [M – H+] 1253.303, found 1253.33).

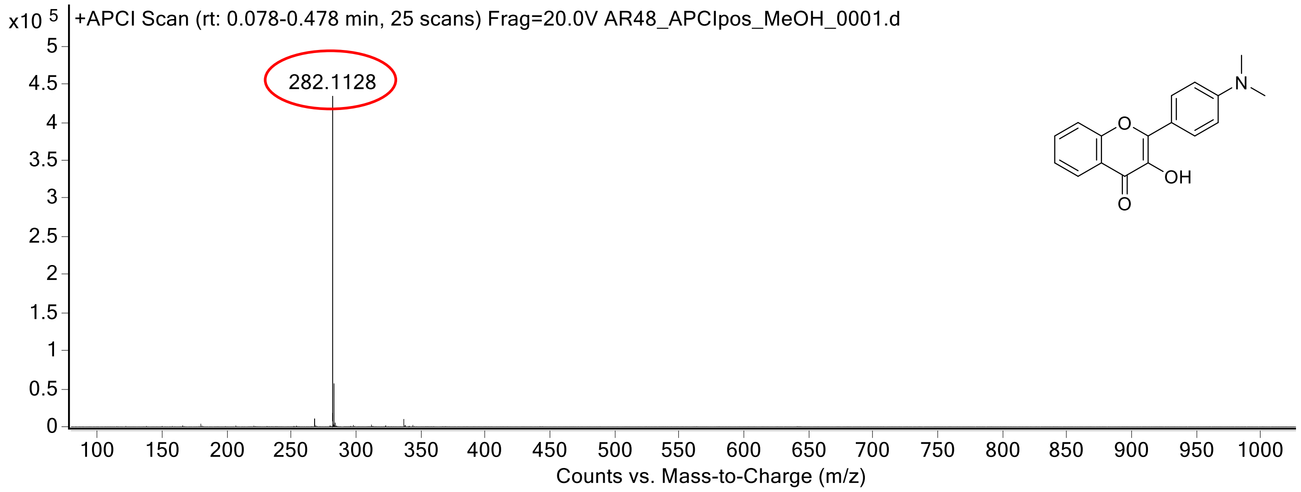

Figure S75. HRMS (APCI+): **20** (calcd. for $C_{17}H_{16}NO_3^+$ [M + H$^+$] 282.1125, found 282.1128).

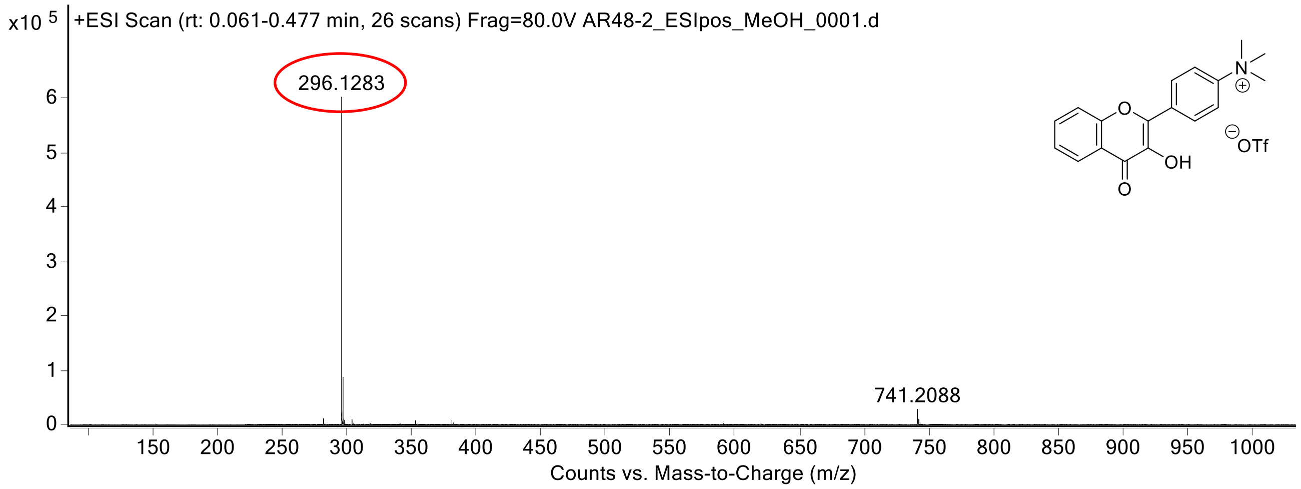

Figure S76. HRMS (ESI+): **10** (calcd. for $C_{18}H_{18}NO_3^+$ [M$^+$] 296.1281, found 296.1283).

References

1. Martinek, M.; Filipova, L.; Galeta, J.; Ludvíkova, L.; Klan, P., Photochemical formation of dibenzosilacyclohept-4-yne for Cu-free click chemistry with azides and 1, 2, 4, 5-Tetrazines. *Org. Lett.* **2016,** *18*, 4892-4895.
2. Prieto-Montero, R.; Sola-Llano, R.; Montero, R.; Longarte, A.; Arbeloa, T.; López-Arbeloa, I.; Martínez-Martínez, V.; Lacombe, S., Methylthio BODIPY as a standard triplet photosensitizer for singlet oxygen production: a photophysical study. *Phys. Chem. Chem. Phys.* **2019,** *21*, 20403-20414.
3. Tanielian, C.; Wolff, C., Porphyrin-sensitized generation of singlet molecular oxygen: comparison of steady-state and time-resolved methods. *J. Phys. Chem.* **1995,** *99*, 9825-9830.
4. Feng, Y.; Vinogradov, I.; Ge, N.-H., General noise suppression scheme with reference detection in heterodyne nonlinear spectroscopy. *Opt. Express* **2017,** *25*, 26262-26279.
5. Vaňková, K.; Marková, I.; Jašprová, J.; Dvořák, A.; Subhanová, I.; Zelenka, J.; Novosádová, I.; Rasl, J.; Vomastek, T.; Sobotka, R.; Muchová, L.; Vítek, L., Chlorophyll-Mediated Changes in the Redox Status of Pancreatic Cancer Cells Are Associated with Its Anticancer Effects. *Oxid. Med. Cell Longev.* **2018,** *2018*, 4069167.
6. Yim, S.-K.; Yun, C.-H.; Ahn, T.; Jung, H.; Pan, J.-G., A Continuous Spectrophotometric Assay for NADPH-cytochrome P450 Reductase Activity Using 3-(4,5-Dimethylthiazol-2-yl)-2,5-diphenyltetrazolium Bromide. *Biochem. Mol. Biol.* **2005,** *38*, 366-9.
7. Vreman, H. J.; Stevenson, D. K., Heme oxygenase activity as measured by carbon monoxide production. *Anal. Biochem.* **1988,** *168*, 31-38.
8. Hutter, E.; Renner, K.; Pfister, G.; Stöckl, P.; Jansen-Dürr, P.; Gnaiger, E., Senescence-associated changes in respiration and oxidative phosphorylation in primary human fibroblasts. *Biochem. J.* **2004,** *380*, 919-28.
9. Smolková, K.; Dvořák, A.; Zelenka, J.; Vítek, L.; Ježek, P., Reductive carboxylation and 2-hydroxyglutarate formation by wild-type IDH2 in breast carcinoma cells. *Int. J. Biochem. Cell Biol.* **2015,** *65*, 125-133.
10. Frisch, M. J.; Trucks, G. W.; Schlegel, H. B.; Scuseria, G. E.; Robb, M. A.; Cheeseman, J. R.; Scalmani, G.; Barone, V.; Petersson, G. A.; Nakatsuji, H.; Li, X.; Caricato, M.; Marenich, A. V.; Bloino, J.; Janesko, B. G.; Gomperts, R.; Mennucci, B.; Hratchian, H. P.; Ortiz, J. V.; Izmaylov, A. F.; Sonnenberg, J. L.; Williams; Ding, F.; Lipparini, F.; Egidi, F.; Goings, J.; Peng, B.; Petrone, A.; Henderson, T.; Ranasinghe, D.; Zakrzewski, V. G.; Gao, J.; Rega, N.; Zheng, G.; Liang, W.; Hada, M.; Ehara, M.; Toyota, K.; Fukuda, R.; Hasegawa, J.; Ishida, M.; Nakajima, T.; Honda, Y.; Kitao, O.; Nakai, H.; Vreven, T.; Throssell, K.; Montgomery Jr., J. A.; Peralta, J. E.; Ogliaro, F.; Bearpark, M. J.; Heyd, J. J.; Brothers, E. N.; Kudin, K. N.; Staroverov, V. N.; Keith, T. A.; Kobayashi, R.; Normand, J.; Raghavachari, K.; Rendell, A. P.; Burant, J. C.; Iyengar, S. S.; Tomasi, J.; Cossi, M.; Millam, J. M.; Klene, M.; Adamo, C.; Cammi, R.; Ochterski, J. W.; Martin, R. L.; Morokuma, K.; Farkas, O.; Foresman, J. B.; Fox, D. J. *Gaussian 16 Rev. D.01*, Wallingford, CT, 2016.
11. Malloum, A.; Fifen, J. J.; Conradie, J., Solvation energies of the proton in methanol revisited and temperature effects. *Phys. Chem. Chem. Phys.* **2018,** *20*, 29184-29206.
12. Kucherak, O. A.; Shvadchak, V. V.; Kyriukha, Y. A.; Yushchenko, D. A., Synthesis of a fluorescent probe for sensing multiple protein states. *Eur. J. Org. Chem.* **2018,** *2018*, 5155-5162.
13. Ueda, T.; Konishi, H.; Manabe, K., Palladium-Catalyzed Reductive Carbonylation of Aryl Halides with N-Formylsaccharin as a CO Source. *Angew. Chem. Int. Ed.* **2013,** *52*, 8611-8615.
14. Xu, G.; Lei, H.; Zhou, G.; Zhang, C.; Xie, L.; Zhang, W.; Cao, R., Boosting hydrogen evolution by using covalent frameworks of fluorinated cobalt porphyrins supported on carbon nanotubes. *Chem. Commun.* **2019,** *55*, 12647-12650.
15. Gunduz, S.; Goren, A. C.; Ozturk, T., Facile syntheses of 3-hydroxyflavones. *Org. Lett.* **2012,** *14*, 1576-1579.
